# *Sim1*-expressing cells illuminate the origin and course of migration of the nucleus of the lateral olfactory tract in the mouse amygdala

**DOI:** 10.1007/s00429-020-02197-1

**Published:** 2021-01-25

**Authors:** Elena Garcia-Calero, Lara López-González, Margaret Martínez-de-la-Torre, Chen-Ming Fan, Luis Puelles

**Affiliations:** 1 University of Murcia, IMIB-Arrixaca Institute of Biomedical Research, 30120 El Palmar, Murcia Spain; 2 Department of Embryology, Carnegie Institution for Science, 3520 San Martin Drive, Baltimore, MD 21218 USA

**Keywords:** Hypothalamo-amygdalar corridor, Hypothalamus, Pallial amygdala, Subpallial amygdala, Paraventricular nucleus, Pallium models

## Abstract

We focus this report on the nucleus of the lateral olfactory tract (NLOT), a superficial amygdalar nucleus receiving olfactory input. Mixed with its *Tbr1*-expressing layer 2 pyramidal cell population (NLOT2), there are *Sim1*-expressing cells whose embryonic origin and mode of arrival remain unclear. We examined this population with *Sim1*-ISH and a *Sim1*-tauLacZ mouse line. An alar hypothalamic origin is apparent at the paraventricular area, which expresses *Sim1* precociously. This progenitor area shows at E10.5 a *Sim1*-expressing dorsal prolongation that crosses the telencephalic stalk and follows the terminal sulcus, reaching the caudomedial end of the pallial amygdala. We conceive this *Sim1*-expressing *hypothalamo-amygdalar corridor* (HyA) as an evaginated part of the hypothalamic paraventricular area, which participates in the production of *Sim1*-expressing cells. From E13.5 onwards, *Sim1-*expressing cells migrated via the HyA penetrate the *posterior* pallial amygdalar radial unit and associate therein to the incipient *Tbr1*-expressing migration stream which swings medially past the amygdalar anterior basolateral nucleus (E15.5), crosses the pallio-subpallial boundary (E16.5), and forms the NLOT2 within the anterior amygdala by E17.5. We conclude that the *Tbr1*-expressing NLOT2 cells arise strictly within the *posterior* pallial amygdalar unit, involving a variety of required gene functions we discuss. Our results are consistent with the experimental data on NLOT2 origin reported by Remedios et al. (Nat Neurosci 10:1141–1150, 2007), but we disagree on their implication in this process of the dorsal pallium, observed to be distant from the amygdala.

## Introduction

The mammalian amygdalar pallial complex consists of a heterogeneous group of nuclei located in the telencephalic temporal pole, rostrally to the caudo ventral hippocampus. They are implicated in reward evaluation of stimuli and emotional learning (Burdach 1819–1822; Johnston 1923; Loo 1930; Weiskrantz 1956; Price et al. 1987; De Olmos et al. 1985, 2004; Alheid et al. 1995; Gloor 1997; Swanson and Petrovich 1998; Amaral et al. 2003; Sah et al. 2003; Phelps and Ledoux 2005; Ledoux 2007; Whalen and Phelps 2009; Rolls 2014, 2015; Olucha-Bordonau et al. 2015; Medina et al. 2017). The nucleus of the lateral olfactory tract (NLOT), the bed nucleus of the accessory olfactory tract (BAOT) and the posteromedial cortical nucleus receive olfactory bulb input (Scalia and Winans 1975; Martinez-Marcos and Halpern 2006; Pro-Sistiaga et al. 2007; Igarashi et al. 2012).

We examine in this work the developing *Sim1*- and *Tbr1*-expressing populations of NLOT layer 2, whose postulated neocortical origin (Remedios et al. 2007) seems inconsistent with our results and standard rodent brain atlas data (Puelles et al. 2019a; see “Discussion”). The NLOT is an isolated tri-laminar ovoid cell mass, which lies embedded (after its migration) within the subpallial anterior amygdala, just rostrally to the anterior cortical nucleus or ACo (Krettek and Price 1978; De Olmos et al. 1985, 2004; Martinez-Garcia et al. 2012; Igarashi et al. 2012; Olucha-Bordonau et al. 2015). Nissl staining subdivides the NLOT nucleus in three cell layers. Layer 1 (NLOT1) is a subpial molecular zone with scattered neurons, which receives mitral cell input from the main olfactory bulb (Igarashi et al. 2012). Layer 2 (NLOT2) is a thick and dense corticoid aggregate of pyramidal neurons of medium size with apical dendrites entering NLOT1. Layer 3 (NLOT3) is a multiform layer disposed deep to layer 2. It contains a mixture of small and large neurons (De Olmos et al. 1985, 2004), some of them possibly representing inhibitory interneurons of subpallial origin (i.e., they express *Dlx5* and *Lhx6*; Marín and Rubenstein 2001; García-López et al. 2008). Recently Garcia-Calero et al. (2020) described in addition a cell-sparse NLOT *shell formation* of genoarchitecturally distinct neurons (*Azin2*-, *Er81*, and *Cyp26*-positive) contributing likewise to layer 3. This shell is continuous with a tail of similar neurons leading backwards along the former migration trail of the NLOT2 up to the medial horn of the basolateral nucleus (BLA). We named the new entity the ‘*amygdalo-olfactory stream*’ (Garcia-Calero et al. 2020).

The adult NLOT is located as a whole superficially within the subpallial anterior amygdala (AA) domain. Its heterogeneous neuronal composition suggests different neuroepithelial origins of the cells that populate its three layers (Garcia-Lopez et al. 2008; Medina et al. 2017). NLOT2 was hypothesized to represent dorsopallial (neocortical) cells migrated via a characteristic caudal amygdalar migratory stream (CAS), forming ‘a link between neocortex and the amygdala’ (Remedios et al. 2007; Deussing and Wurst 2007; Murillo et al. 2015; Ruiz-Reig et al. 2017). Puelles et al. (2019a) recently expressed doubt about this interpretation of NLOT2, due to the distant position of the molecularly defined dorsal pallium (neocortex) from the amygdala, with large interposed portions of mesocortex and allocortex. The majority of NLOT2 cells express the pallial mantle marker *Tbr1*, consistently with a pallial origin, whereas other cells express *Sim1,* a marker otherwise mainly present in the hypothalamus (Fan et al. 1996; Michaud et al. 1998; Balthasar et al. 2005). The transient NLOT2 migratory stream (NLOT2ms), or CAS, appears labelled by the markers *Neurod1*, *Neurod2*, *Tbr1*, *Math2 (Neurod6), SCIP* and *Zic2* (Remedios et al. 2007; Murillo et al. 2015; see also *Dach1* signal at the NLOT2ms in the Allen Developing Mouse Brain Atlas). This stream appears in sagittal sections of E14.5–E17.5 mouse embryos, and the definitive nucleus forms within the subpallial anterior amygdala (AA) between E17.5 and E18.5 (Remedios et al. 2007).

Mutant mice lacking *Emx1/Emx2*, *Lhx2, Pax6* or *Zic2* functions do not develop the NLOT2 (Remedios et al. 2004; Tole et al. 2005; Murillo et al. 2015). There is so far no rationale indicating how these diverse determinants interact to produce the definitive NLOT structure. We will present a tentative synthesis on how these diverse genes control NLOT2 development, including also the presently studied *Sim1* case, since we observed that loss of *Sim1* signal likewise impedes the final formation of the NLOT.

The transcription factor *Sim1* is expressed in several separate regions of the central nervous system apart the NLOT2, e.g., in the basomedial amygdala, as well as in alar and basal hypothalamic regions (Fan et al. 1996; Wang and Lufkin 2000; Balthasar et al. 2005; Puelles et al. 2012). This gene is necessary for normal development and terminal differentiation of diverse cell types within the paraventricular and supraoptic nuclei in the alar hypothalamic region (Fan et al. 1996; Michaud et al. 1998). *Sim1-*positive neurons derived from the paraventricular area (Pa) reportedly regulate food intake and energy expenditure, and mice heterozygous for *Sim1* are obese (Michaud et al. 2001; Holder et al. 2004; Tolson et al. 2010). The melanocortin-4-receptor pathway (MC4R) apparently mediates the function of *Sim1-*expressing cells within hypothalamic Pa and amygdalar NLOT (Balthasar et al. 2005). MC4R expression appears only after E18.5 at the post-migratory NLOT2, in parallel with some mesocortical areas and the subiculum (Allen Developing Mouse Brain Atlas). In addition, the Rett syndrome, a pathology belonging to autism spectrum disorders, relates to loss of *Mecp2* signal in *Sim1-*expressing cells at the medial amygdala and the NLOT (Fyffe et al. 2008).

The most probable origin of NLOT2 *Sim1*-expressing cells is the hypothalamic paraventricular area or Pa (Fan et al. 1996; Puelles et al. 2012). It is already known that *Otp-*expressing cells of Pa origin migrate into the medial amygdala (Wang and Lufkin 2000; Garcia-Moreno et al. 2010; Morales-Delgado et al. 2011). However, disruption of the *Otp* gene in mice does not affect the expression of *Sim1* in the normally migrated NLOT2 (Wang and Lufkin 2000).

In the present report, we first examined the presumptive extratelencephalic Pa source of *Sim1-*positive cells, and their migration path across the hemispheric stalk, until they reach the caudomedial pallial amygdala. To this end, we investigated in detail the developmental progression of the *Sim1 *in situ expression pattern and *Sim1*-tauLacZ-labeled progeny in correlation with other regional markers. We identified the pathway followed by the *Sim1* cells as the *hypothalamo-amygdalar corridor* (HyA). Next, we explored how such cells, once arrived at the amygdala, incorporate into the NLOT2 migration stream to reach the NLOT2 target.

In the second phase of this study, we related the amygdalar population of *Sim1*-expressing cells to the rostrally migrating stream of *Tbr1*-expressing pallial cells which constitute what we call the *NLOT2 migratory stream* (NLOT2ms), also known as *caudal amygdalar stream*, or CAS (first described by Remedios et al. 2007). We found that this pallial stream starts at the *posterior* pallial amygdalar unit we recently defined (Garcia-Calero et al. 2020), which is the anlage of the amygdalo-hippocampal area and the posteromedial cortical nucleus (AHi/PMCo complex). This locus is precisely where the migrating hypothalamic *Sim1* cells arrive via the HyA. The NLOT2ms thus carries from its origin pallial *Tbr1*-expressing and hypothalamic *Sim1*-expressing cells (or *Sim1*-tauLacZ labeled ones). This mixed cellular stream advances first within pallial amygdala next to the pallio-subpallial limit, until it reaches the medial aspect of the BLA and BMA nuclei. The stream then crosses the pallio-subpallial border roughly at E15.5–E16.5, and the migrating mass approaches radially its superficial target site within the subpallial anterior amygdala area (AA), rounding up thereafter as the NLOT2. This mechanism thus translocates *Sim1-* and *Tbr1-*positive cells from the *posterior* pallial amygdalar unit (AHi/PMCo primordium) into the superficial NLOT2.

While our results on the NLOT2 migration itself corroborate the relevant data of Remedios et al. (2007), we disagree regarding their characterization of the amygdalar locus of origin as a dorsopallial extension, as well as on their implicit parallel conclusion that neocortex reaches caudally the posterior amygdala (being ‘linked to it’, as is affirmed in their title). We consider in the Discussion the doubtful assumptions held for years in this field of research that possibly caused the cited disagreements. We conclude that hypothalamic *Sim1* cells incorporate into the NLOT2 pallial migratory stream at its true *amygdalar* origin, i.e., the *posterior* pallial amygdalar radial unit, after arriving there at E12.5–E13.5 via the *hypothalamo-amygdalar corridor* (HyA). We also observed that homozygous *Sim1* loss-of-function (obtained from a *Sim1*-tauLacZ mouse line) does not alter the initial phase of migration of *Sim1*-expressing cells into the NLOT2ms, but these cells stop their advance at E16.5 and many of them presumably die without advancing into the final subpallial phase of migration. This also impedes the final steps of migration of the accompanying pallial *Tbr1*-positive components of the NLOT2, revealing a functional interaction between the mixed *Tbr1* and *Sim1* cell populations. This pattern (cell death at the time of final differentiation) already emerged at the hypothalamic derivatives of the paraventricular area, using the same mouse line (Michaud et al. 1998).

## Results

### *Sim1* expression during amygdalar development at early embryonic stages

**Fig. 1:**
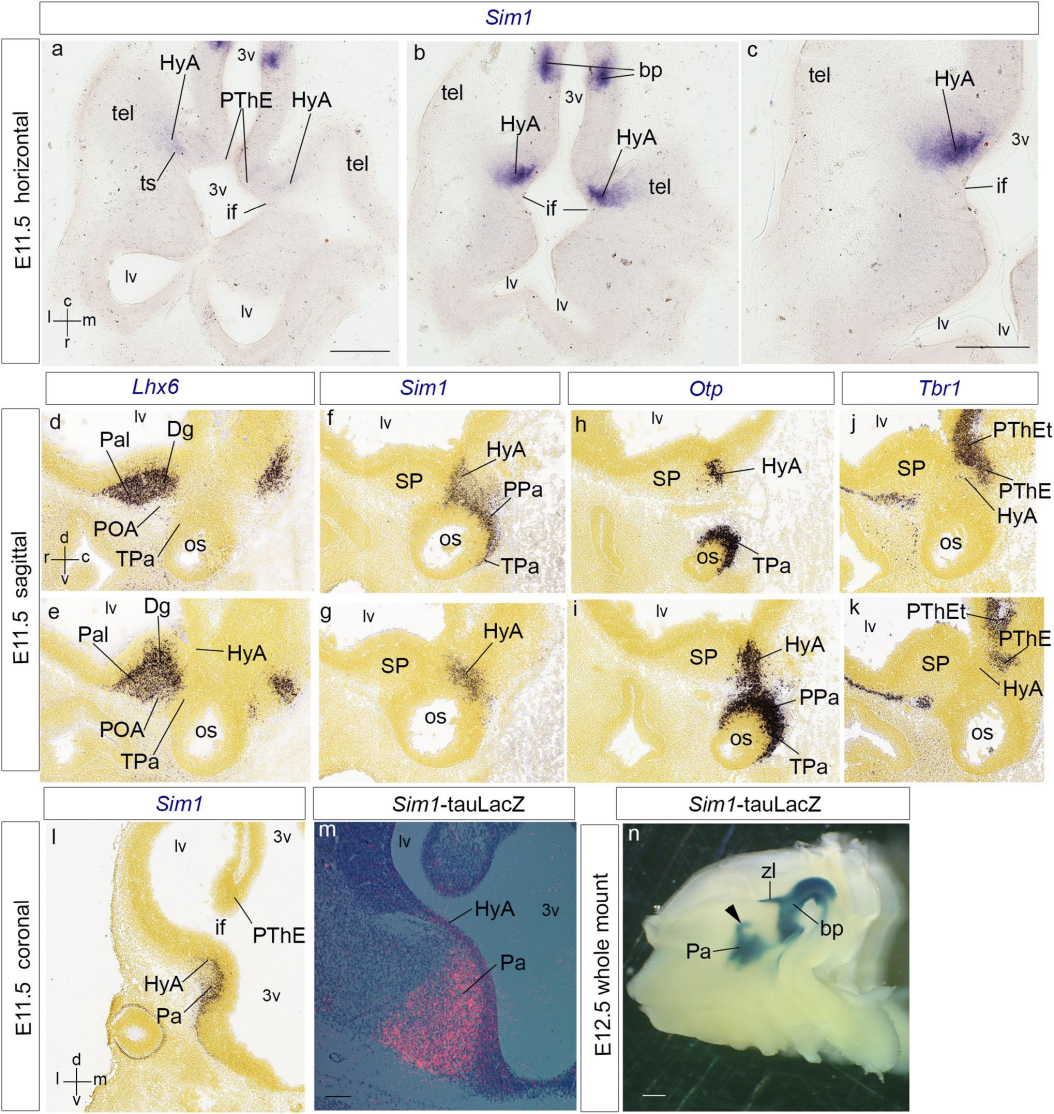
Expression of *Sim1*, *Lhx6, Otp*, or *Tbr1* in the developing mouse secondary prosencephalon at E11.5/12.5 (**a**–**m**); the section plane is indicated at the side jointly with the gestation day; spatial orientation is indicated at the bottom left-hand corners of panels (**a**, **d**,** l**); **d**–**l** illustrations from Website: ©2013 Allen Institute for Brain Science. Allen Developing Mouse Brain Atlas. http://developingmouse.brain-map.org). **a**–**c** *Sim1* expression in horizontal sections; **a**, **b** sections ordered dorso-ventrally illustrating the *Sim1*-expressing hypothalamo-amygdalar corridor (HyA) along the terminal sulcus (ts) and near the interventricular foramen (if); **c** this figure is a higher magnification detail of **b**. **d**–**k** Pairs of comparable sagittal sections at E11.5, each pair ordered from lateral to medial, and labelled with different markers, for mutual comparison. **d**, **e** Pallidal and diagonal expression of *Lhx6* at the subpallium (Pal, Dg). Note correspondence of mantle signal with the incipient ventricular bulge of the medial ganglionic eminence. The labelled domain has a sharp caudal boundary at the telencephalic stalk. **f**, **g** *Sim1* expression at the peduncular and terminal paraventricular hypothalamic areas (PPa, TPa), extending dorsally into the HyA at the stalk. Note sharp rostral limit of HyA, correlative with the *Lhx6*-positive domain in **d**,** e**. **h**, **i** *Otp* expression to compare with *Sim1* signal in **f**,** g**. **j**, **k** *Tbr1* signal to illustrate the position of the diencephalic prethalamic eminence (PThE) caudal to the HyA. **l**,** m** *Sim1* expression in coronal sections at E11.5. Note Pa expression expanding dorsalward into the HyA, which enters the hemispheric stalk at the floor of the interventricular foramen (if). **m** A pseudo-colored and Nissl-counterstained *Sim1*-tauLacZ coronal section in a heterozygote specimen; there is strong beta-galactosidase reaction at the Pa and HyA mantle zone ascending into the interventricular foramen (HyA). Note that, at this stage, there is also *Sim1* expression at the PA and HyA ventricular zone. **n** A half-brain whole-mount of a *Sim1*-tauLacZ embryo, reacted for beta-galactosidase at E12.5. The black arrowhead points to the HyA spike coming out of the Pa area and bending backwards (dorsalward, topologically) through the interventricular foramen into the terminal sulcus. Note also the separate patch of the axially bent basal plate signal (bp), reaching rostrally up to the mamillary body and caudally to the isthmus; the zona limitans intrathalamica spike (zli), which limits prethalamus and thalamus, extends dorsalward into the alar territory. For abbreviations, see list. Scale bars represent 200 µm (**a**, **b**, **d**–**l**), 150 µm (**c**), 100 µm (**m**), and 500 µm (**n**)

**Fig. 2:**
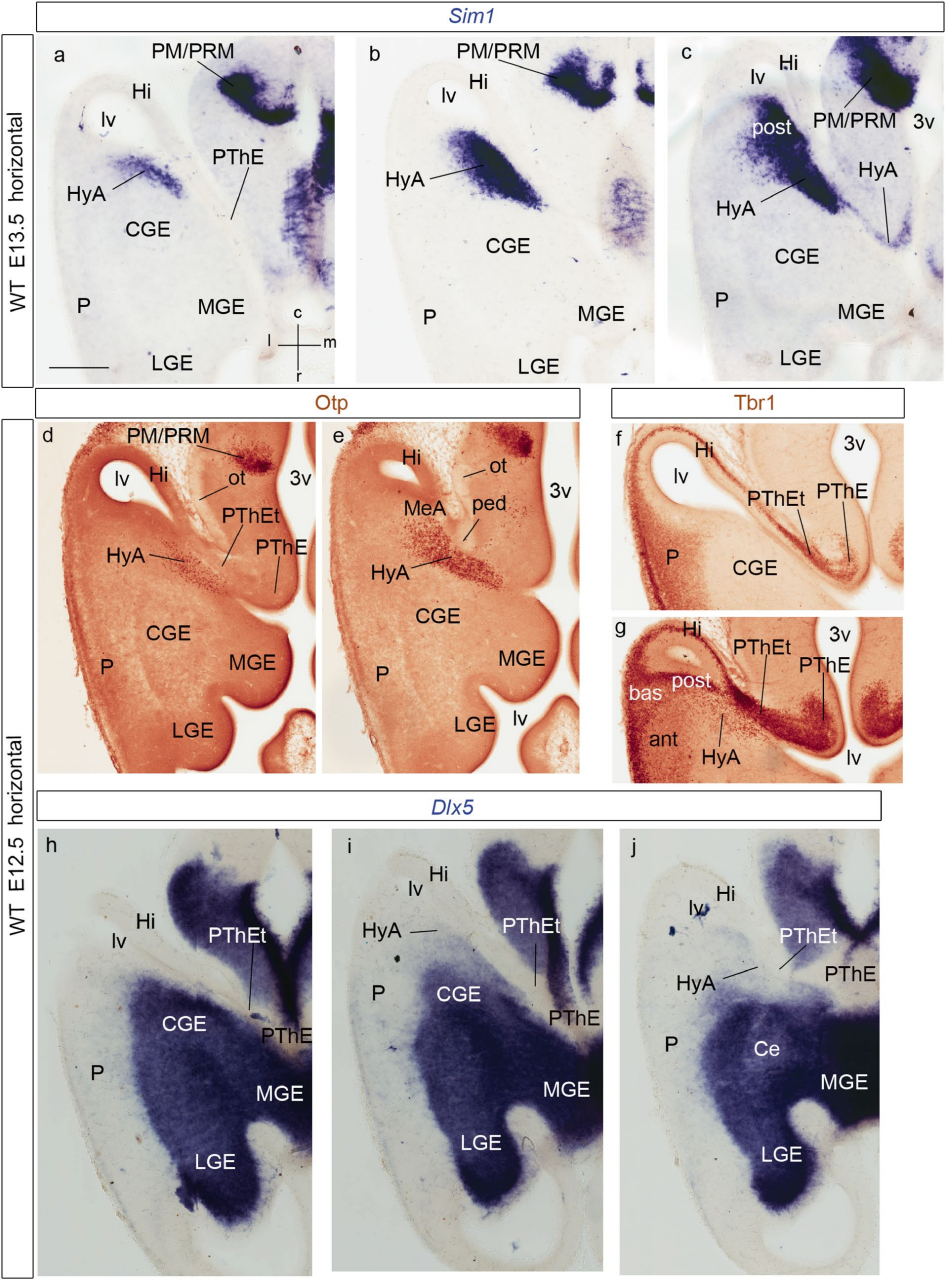
Horizontal telencephalic sections at E13.5 (**a**–**c**) and E12.5 (**d**–**j**), showing expression of *Sim1*, *Otp*, *Tbr1* and *Dlx5*, as indicated above the panels. The spatial orientation appears at the bottom right-hand corner of panel **a**. The images show dorsoventrally ordered section levels through the transition between the hypothalamus (3v) (plus the diencephalic prethalamic eminence; PThE) and the telencephalon, whose ganglionic eminences (LGE, CGE, MGE) are visible in relation to pallial areas (P) and the lateral ventricle (lv). **a**–**c** Panels illustrating *Sim1-expressing cells* dorsally along the hypothalamo-amygdalar corridor at the terminal sulcus (HyA; **a**), reaching the pallial amygdala behind the CGE and in front of the hippocampus (CGE; Hi; **b**), and starting to mix lateralwards with pallial amygdalar cells of the *posterior* amygdalar radial unit, the future AHi (post; **c**). The separate basal domain of *Sim1* expression at the perimamillary/periretromamillary hypothalamic area is visible in the three panels (PM/PRM; **a**–**c**). A less populated part of the HyA also appears at its entrance into the interventricular foramen and terminal sulcus, next to the 3v (**c**). **d**,** e** Panels immunoreacted for Otp. Dorsally, at the level where the HyA lies just under the floor of the terminal sulcus (HyA; **d**), there are few Otp cells, whereas their number increases in a more ventral section (HyA; **e**); the HyA in this case leads into the MeA (MeA; **e**). **f**,** g** Panels immunoreacted for Tbr1. Dorsally (**f**), pallial signal (P) extends around the caudal part of the lateral ventricle (lv) past the hippocampal primordium (Hi) into the evaginated ‘telencephalic’ part of the prethalamic eminence (PThEt) and the PThE proper; no signal of Tbr1 in the underlying central prethalamus, or in the subpallium (CGE); this section level passes above the pallial amygdala. More ventrally (**g**), we distinguish *anterior, basal* and *posterior* radial parts of the pallial amygdala, the latter next to the HyA (ant, bas, post; HyA). Otherwise hippocampal pallium (Hi), as well as PThEt and PThE are likewise immunoreactive for Tbr1. These direct transitions between Hi and PThEt occur below the end of the chorioidal fissure. **h**-**j** These panels illustrate *Dlx5* subpallial and prethalamic in situ hybridization signal (which excludes the PThE/PThEt areas, as well as the telencephalic pallium (Hi, P). The reaction delineates the LGE, CGE and MGE ganglionic eminences. The HyA (compare with **a**–**c**) appears very weakly stained, caudomedially to the CGE and MGE. For abbreviations, see list. Scale bar represents 300 µm

**Fig. 3:**
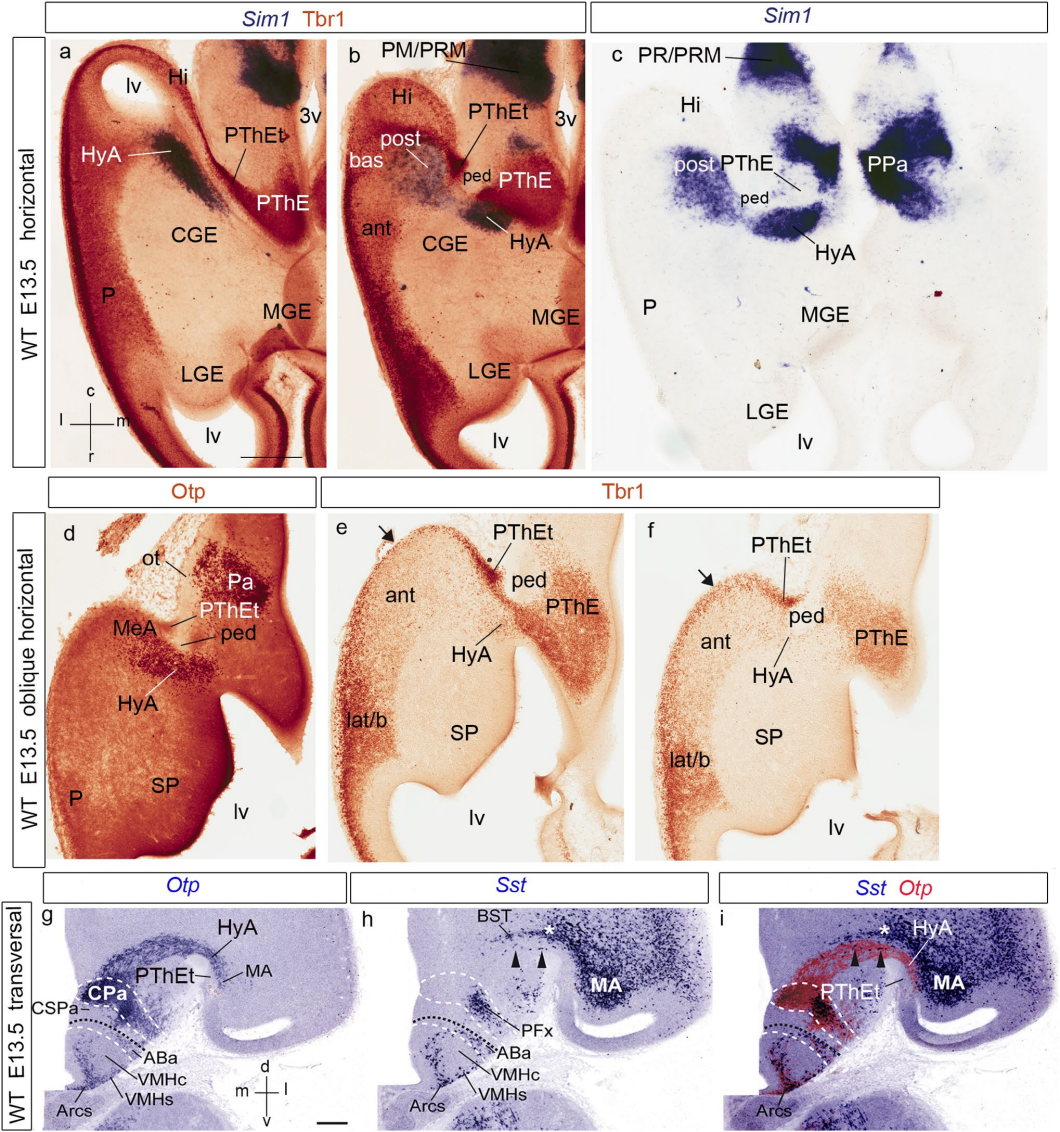
Horizontal (**a**–**c**), oblique horizontal (**d**–**f**) and transversal (**g**–**i**) sections of E13.5 mice telencephalon, showing *Sim1*, Tbr1, Otp, *Otp*, and *Sst* signals, partly combined for comparative purposes, as indicated above the panels. The spatial orientation appears at the bottom left-hand corner of **a**, and the bottom right-hand corner of **g**. **a**, **b** Panels comparing Tbr1 immunoreaction (brown) and *Sim1* transcripts (dark blue). Dorsally, the level of section is similar to that of Fig. 2g; the HyA courses here under the terminal sulcus, skirting the unlabeled CGE, and approaches the pallial amygdala (HyA; **a**). More ventrally, the *anterior*, *basal* and *posterior* radial units of the pallial amygdala appear (ant, bas, post; **b**; note the characteristic lower level of Tbr1 reaction at ant), and the pioneering HyA *Sim1* cells are starting to enter the posterior unit (blue signal; post; **b**). The HyA appears interrupted by the underlying peduncular fibres (ped; **b**). **c** This panel shows a *Sim1*-reacted section similar to that in **b**, but without counterstain, showing likewise the incipient invasion of posterior amygdala by *Sim1* cells, and the peduncular fiber packet located under the HyA (post; ped; **c**). We also see bilaterally the connection of the HyA with the hypothalamic (peduncular) paraventricular area, expressing likewise *Sim1* (PPa; **c**). **d**–**f** These three oblique sections are less than horizontal sections, without being coronal; they progress from rostroventral to dorsocaudal. **d** This panel shows Otp immunoreaction at the paraventricular area (Pa) and the subcapsular part of the HyA, entering straightforwardly the medial amygdala; the peduncle is nearby (HyA; MeA; ped; **d**). **e**, **f** Sections of the same series as **d** immunoreacted for Tbr1 and cut at levels through the pallial amygdala, but rostrally to the posterior unit (latb; ant; **e**, **f**; note again low Tbr1 signal at ant). The Tbr1 reaction at the telencephalic PThE (PThEt) seems to extend at the ventrocaudal pole of the vesicle (under Hi, not seen here) into amygdalar and olfactory pallium (black arrows; **e**, **f**). **g**–**i** Two adjacent transversal sections labeled with *Otp* and *Sst* in situ reaction (**g**, **h**), superposed graphically in **i**. We copied these images with permission from Morales-Delgado et al. (2014). The section level passes through the caudal part of the paraventricular area (CPa). *Otp* signal extends from CPa dorsolaterally through the subcapsular HyA stream into the medial amygdala (MA; **g**). The HyA relates intimately as a neighbor with *Sst* signal along the diagonal subpallial domain of the MGE/CGE, which includes BST elements and the medial amygdala (BST; MA; arrowheads; **h**, **i**). Note that the unlabeled PThEt domain appears medially to the Otp-labeled HyA domain (PThEt; **g**, **i**). The caudal cortex closing the lateral ventricle is the ventral hippocampus (unlabeled). For abbreviations, see list. Scale bars represent 300 µm (**a**–**f**), and 200 µm (**g**–**i**)

**Fig. 4:**
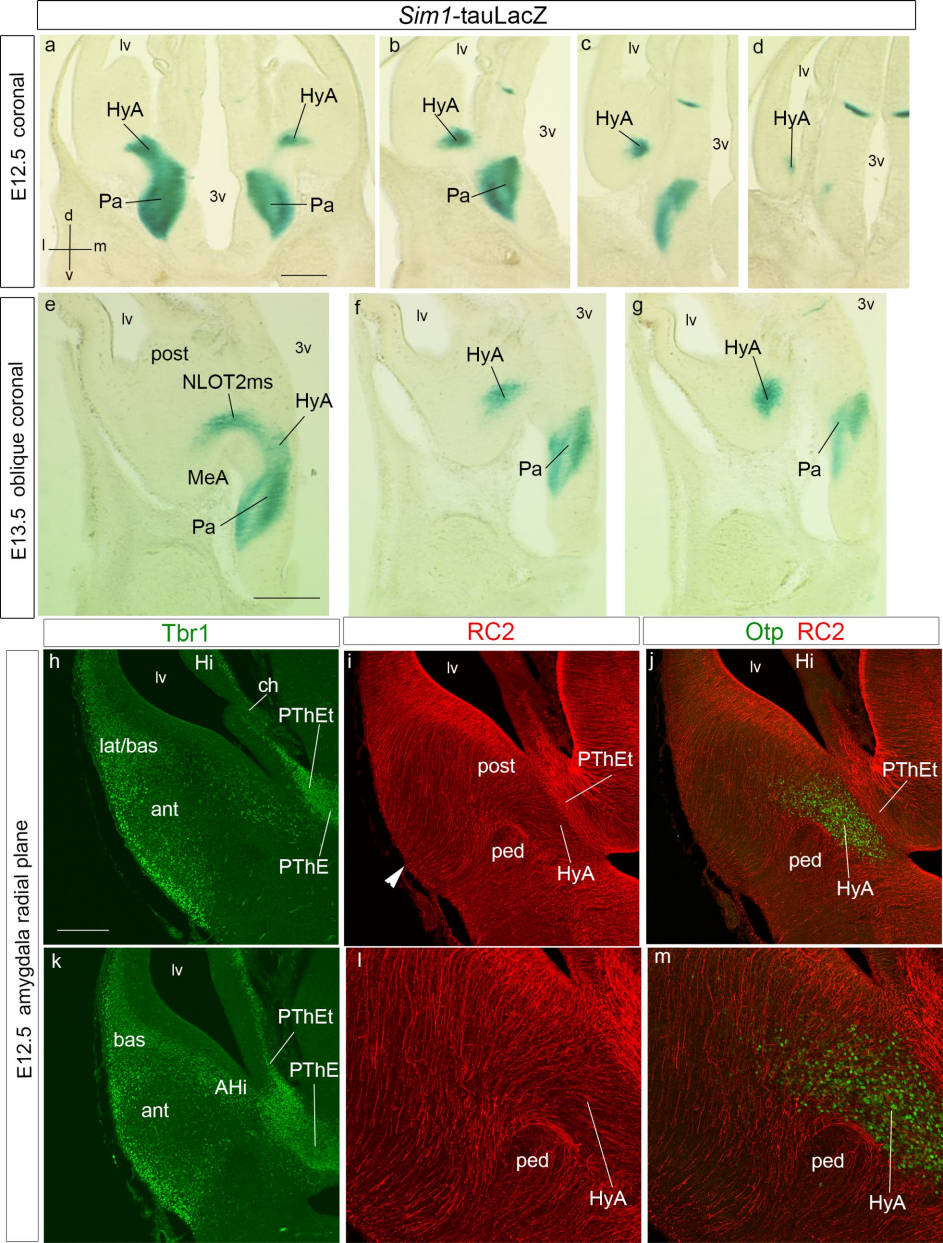
Coronal E12.5 rostrocaudal sections from a *Sim1*-tauLacZ embryo (**a**–**d**; see spatial orientation in **a**), rostrocaudal oblique coronal sections through an E13.5 *Sim*-tauLacZ embryo (**e–g**; similar orientation as in **a**), and radial amygdalar adjacent sections at E12.5 comparing *Tbr1* and *Otp* transcripts with RC2 glial anti-nestin immunoreaction (**h**–**m**). **a**–**d** This series is reacted for beta-galactosidase (green *Sim1*-tauLacZ signal), and shows rostrally at left the continuity between the paraventricular area and the HyA, alevel with the interventricular foramen (Pa; HyA; **a**). Subsequent sections show more ventral parts of Pa, and more caudal (dorsal) parts of HyA along the periventricular stratum under the terminal sulcus, with a tiny caudal tip (Pa; HyA; **b**–**d**). At E12.5 we do not see yet any sign of NLOT2ms. **e**, **g** This series is similar to **a**–**d**, but is cut with a more rostral inclination, favorable to visualizing the first rostrally migrating *Sim1* cells of the NLOT2ma, after these move lateralwards behind the MeA into the *posterior* amygdalar unit (NLOT2ms; MeA; post; **e**). The more caudal parts of the HyA are less advanced (HyA; **f**, **g**). **h**–**m** Immunofluorescent reaction for Tbr1 (green; **h**, **k**), RC2 glial stain (red; **i**, **l**), and superposed Otp (green) and RC2 signals (red; **j**, **m**) at E12.5, cut in the plane of amygdalar radial glia. **l**, **m** Magnified details of **i**, **j**, respectively. Tbr1 identifies various parts of amygdalar pallium, as well as the pallio-subpallial boundary (ant, bas, lat/bas, AHi/post; **h**, **k**). Radial glia of the HyA originates at the terminal sulcus (HyA; **i**, **l**), and contours laterally the peduncular fiber packet (ped), which relates to the pallio-subpallial boundary seen in **k**. The Otp cells characterize the position of the HyA in the same section (HyA; **j**, **m**). For abbreviations, see list. Scale bars represent 420 µm (**a**–**g**), 150 µm (**h**–**k**), and 200 µm (**l**, **m**)

We first analyzed the hypothalamo-telencephalic expression pattern of *Sim1* in mouse embryos at early developmental stages (E11.5–E13.5), using horizontal sections (relative to the prosomeric forebrain axis), in addition to conventional coronal and sagittal section planes (Figs. 1, 2, 3, 4, see also Fig. 1a in Puelles et al. 2016a; Fig. 1d-l were downloaded from the Allen Developing Mouse Brain Atlas). We compared *Sim1* gene expression with gene/protein markers present in the hemispheric stalk region, such as *Otp*/Otp (Pa), *Lhx6* (subpallial diagonal area), *Dlx5* (subpallium as a whole) and *Tbr1*/Tbr1 (prethalamic eminence). We subsequently compared immunocytochemically *Sim1* signal with the expression of Otp and Tbr1 proteins, and with the radial glia marker RC2, applying the recently proposed *amygdalar radial section plane* (Garcia-Calero et al. 2020). This plane is oblique to the conventional coronal plane, and forms a varying angle of 30°–45° with reference to a line tangent to the entorhinal cortex at the back of the hemisphere; this plane agrees with the spatial disposition of radial glial processes crossing the amygdalar pallial region (Fig. 4h–m).

At E11.5 and E12.5, early *Sim1* transcripts appear in the ventricular and mantle layers of the hypothalamic paraventricular area (Pa; Fig.1l, m). This pattern extends dorsalward in front of the diencephalic prethalamic eminence (PThE) into a curved spike that enters the telencephalic vesicle through the floor of the interventricular foramen (Pa; PThE; if; ts; Figs. 1a–c, f, g, 4a–d; see also E12.5 whole-mount in Fig. 1n). This labeled spike extends caudalwards (topologically dorsalward) along the terminal sulcus (Figs. 1a–c; 4a–d), reaching the posterior amygdalar area at E12.5 (data also at E13.5; Figs. 2a–c, 4a–e). There is partial overlap of the *Sim1* and *Otp* signals at the stalk and terminal sulcus mantle (Figs. 1f–i, 2a–e, 4j, m).

We named the telencephalic spike-like extension of the paraventricular mantle (first described, but left unnamed in Fan et al. 1996) the *hypothalamo-amygdalar corridor,* interpreting that it carries hypothalamic cells reported to migrate into the amygdala (HyA; Figs. 1a–c, f–i, 2a–e; present data and Wang and Lufkin 2000; Garcia-Moreno et al. 2010; Morales-Delgado et al. 2011; see Fig. 3g–i). The peduncular hypothalamic paraventricular area or Pa (PPa) is continuous rostrally with terminal Pa (TPa) within the terminal hypothalamus (THy); expression of both *Sim1* and *Otp* reaches the acroterminal optic stalk area (os; PPa; TPa; Fig. 1f–i; see also Morales-Delgado et al. 2011; Puelles and Rubenstein 2015).

At E11.5 the HyA distinctly limits rostrolaterally at the telencephalic stalk with the *Lhx6*-expressing primordium of the medial and caudal ganglionic eminences (MGE, CGE), which include the prospective pallidal and diagonal subpallial areas (Pal; Dg; Garcia-Lopez et al. 2008; Bupesh et al. 2011; Puelles et al. 2016a; Pal; Dg; Fig. 1d, e). Caudomedially, HyA limits with the *Tbr1*-expressing, partially evaginated ‘telencephalic’ portion of the rostral diencephalic prethalamic eminence (PThEt; Fig. 1j, k). The subpallial diagonal (Dg) area will form the *Sst*-expressing *principal*, *supracapsular* and *medial amygdalar* components of the stria terminalis complex ending at the medial amygdala, which are adjacent, but perfectly distinct from our HyA (BST, MA, HyA; see Fig. 3h, i; Morales-Delgado et al. 2011; Puelles et al. 2013, 2016a). Note we previously identified the Dg area as ‘anterior entopeduncular area’ (AEP; Bulfone et al. 1993, 1995; Puelles and Rubenstein 1993; Puelles et al. 2000). This name derived from classical literature, but was confusing, since ‘entopeduncular’ does not apply properly as a descriptor to a full radial domain (i.e., it refers only to the mantle stratum interstitial to the peduncle). On occasion of preparing the reference atlases and ontology for the Allen Developing Mouse Brain Atlas (during 2008–2011), LP changed the name of this domain to ‘diagonal domain or area’ (Dg). This refers explicitly to the radial domain that includes *periventricular* BST elements, *intermediate* substantia innominata structures including the basal nucleus of Meynert (SI) and *superficial* diagonal band (DB) components (Puelles et al. 2013; Thompson et al. 2014). The external anatomic relief of the subpial diagonal band tract serves for topographic identification of the Dg, which limits the HyA throughout its length. Medina and Abellán (2012) refer to the AEP/Dg domain as ‘caudoventral MGE’.

As mentioned above, caudomedially to the evaginated Pa spike, or HyA, lies the *evaginated PThE* or PThEt (a piece of rostrodorsal prethalamic alar diencephalon), which already forms at this stage a part of the caudomedial wall of the hemisphere, ending at the chorioidal fissure. The ventricular surface of the evaginated portion of PThE bends around the *caudal* border of the interventricular foramen (just behind the HyA at its floor) into the inner wall of the terminal sulcus (HyA lies along the sulcus proper). Both PThEt and HyA extend all the way into the roof-derived chorioidal fissure (see schema in Fig. 15a). The PThE region as a whole represents the dorsalmost histogenetic area of the prethalamic domain (the alar plate of prosomere 3; Puelles et al. 2020), whose *Dlx*-negative mantle layer (PThE; Fig. 2h–j) is characteristically labelled by *Tbr1* and *Lhx9* (PThE; Figs. 1j, k; 2f, g; see other markers in Puelles et al. 2012, 2016a, 2020). *Tbr1* also labels the mantle layer of the telencephalic pallium, whose hippocampal and amygdalar subdivisions are separated by the final part of the hypothalamo-amygdalar corridor (HyA) from direct contact with the evaginated or ‘telencephalic’ PThE (PThEt) at its topologically rostrodorsal end (PThEt; Hi; Fig. 2f, g).

The selective subpallial *Dlx5/Lhx6/Sst* markers as well as the pallial/eminential *Tbr1* marker accordingly identify differentially at E13.5 and later stages the embryologically diverse structural components present in a cross-section at the cryptic caudal end of the telencephalic stalk and the caudal amygdalar region. Rostromedial to the pallial amygdala there is the *Dlx5*/*Lhx6*-expressing (and CB/Sst-positive) subpallial *diagonal domain* ending at the medial amygdala (BST; MeA; Fig. 3h, i; see also Fig. 15a). Medially to the medial amygdala, there lies at the floor of the terminal sulcus the *Sim1/Otp*-expressing hypothalamic Pa spike or *hypothalamo-amygdalar corridor* (HyA; Figs. 3i; 15a). Finally, medially to the HyA there is the evaginated or ‘telencephalic’ part of the diencephalic PThE (PThEt) (Fig. 3i; see also horizontal sections in Figs. 2, 3a–f).

At E12.5/E13.5, *Sim1/Otp* transcripts and *Sim1*-tauLacZ labeling still appear along the *hypothalamo-amygdalar corridor* (HyA; Figs. 2a–e, 3a–i, 4e). The narrow *Sim1*-expressing area overlaps in part the band of Otp protein expression, but reaches essentially the posterior *pallial* amygdala coinciding with scattered Tbr1-immunopositive cells (HyA; Figs. 2g, 3a–f). The Otp-positive cells apparently end instead at the *subpallial* medial amygdala (compare Fig. 3a–c for *Sim1* with Fig. 3d for Otp, which displays a more ventral *subcapsular* section level, where no *Sim1* signal is found; see also Wang and Lufkin 2000; Garcia-Moreno et al. 2010; other *Otp* cells accompany the HyA to its dorsal end at the chorioidal fissure; EG-C and LP, unpublished observations). The HyA corridor always contacts rostrally and laterally the *Dlx5* positive and *Tbr1*-negative subpallial diagonal domain (along the medial portion of the medial and caudal ganglionic eminences), but is itself a region devoid of *Dlx5* transcripts (HyA; CGE; MGE; Figs. 2h–j, 3a, b). It represents the evaginated part of the hypothalamic Pa area along the floor of the terminal sulcus of the lateral ventricle.

The lateral wall of this sulcus is built by the medial and caudal ganglionic eminences, bulges into the ventricle, and is continuous caudally with the medial amygdala. The main part of the ganglionic eminences reaches the brain surface at the olfactory tuberculum (striatum and pallidum), while the diagonal domain ends along the superficially prominent diagonal band, and the preoptic area has its own pial surface. The lateral ventricle wall limiting medially the HyA corresponds to the evaginated part of the PThE, whose surface lies at the pial hemispheric sulcus (PThE; chf; Hi; HyA; Figs. 2a–g, 3; see also Fig. 15a). The neuroepithelial HyA corridor lying in between MGE/CGE and PThEt actually represents a radially complete histogenetic area that reaches the brain surface, but its hypothetic intermediate and superficial mantle elements remain poorly studied (probably lumped with the MeA). We estimate that the brain surface corresponding to the HyA corridor proper, which converges ventrally with the hypothalamic Pa area, probably lies medially adjacent to the telencephalic diagonal band (not shown; see Fig. 15a).

We compared Otp immuno-fluorescent signal at the hypothalamic Pa region and related HyA corridor with Tbr1 signal (PThE, and amygdalar or hippocampal pallial areas) and RC2-immunoreactive glial fibres in sections taken in the *amygdalar radial section plane* (Fig. 4h–m). The Tbr1-positive mantle of the evaginated PThE reaches the roof plate-derived chorioidal *tela* at the prethalamic taenia of the chorioidal fissure, and also contacts directly the caudal end of the hippocampal area (PThE; ch; Hi; Fig. 4h), as well as the amygdalo-hippocampal area, beyond the end of the chorioidal fissure (AHi; Fig. 4k; see also Fig. 15a). The Otp*-*labeled HyA corridor entering the telencephalic MeA area is separated at this level from the PThE by the compressed walls of the terminal sulcus; there are scarce Tbr1-positive cells (HyA; PThE; Fig. 4h, j, k, m). Otp cells are found later restricted to the medial amygdala, which may include unrecognized remnants of the HyA (Wang and Lufkin 2000; Garcia-Moreno et al. 2010). By co-immunolabeling with the RC2 marker, both HyA corridor-related and PThE-related glial fibres show their distinct radial trajectories; the HyA radial glial cells end superficially at the brain surface *outside* the peduncular subpallium (ped; HyA; PThE; Fig. 4i–m).

### Telencephalic *Sim1* expression at intermediate developmental stages

**Fig. 5:**
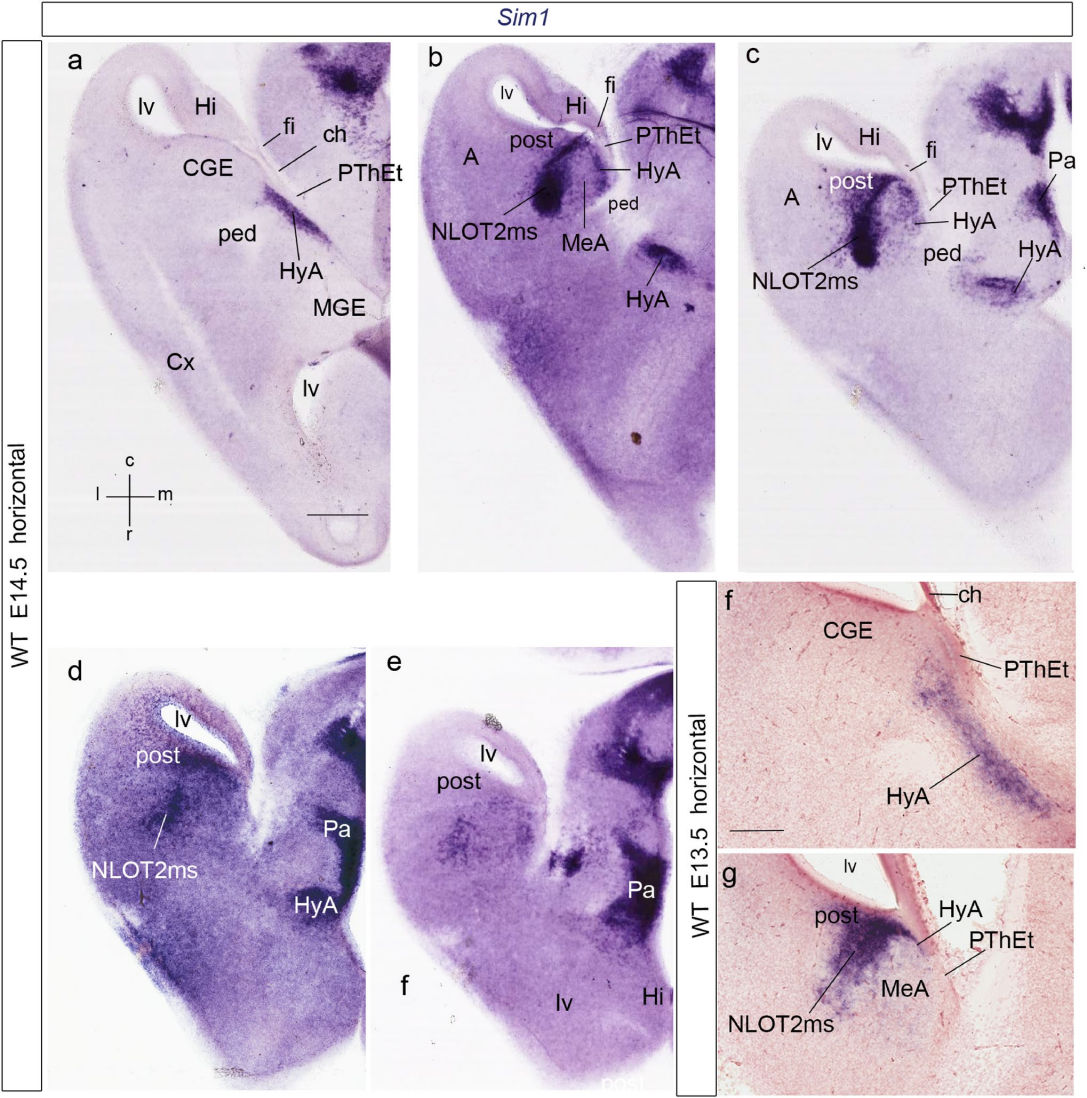
Horizontal sections through the telencephalon in two embryos at E14.5 (**a**–**e**) and E13.5 (**f**, **g**), showing *Sim1* transcripts at the HyA and the beginning of the NLOT2ms. **a**–**e** Dorsally, the HyA advances over the peduncle (ped) under the terminal sulcus. As it reaches the posterior pallial amygdala (**a**; see also **f** at E13.5), the HyA appears as a labeled thin radial domain intercalated between the MeA (laterally) and the PThEt (medially), both unlabeled (HyA; MeA; PThEt; **b**, **c**). Note the close position of the hippocampus and the fimbria/hem (Hi; fi; **b**, **c**). At E14.5, the *Sim1*-positive NLOT2ms appears pedunculated, that is, connected by a thinner stalk to the *posterior* amygdalar ventricular zone, and displaying a rostrally protruding thicker rounded mass (post; NLOT2ms; **b**, **c**). Passing into the ventralmost (caudalmost) sections, the NLOT2ms shows a more retarded appearance, and is accompanied by labeled cells apparently passing lateralwards along the *posterior* amygdalar subventricular zone (post; NLOT2ms; **d**, **e**). Compare with the similar aspect found at E13.5 (post; NLOT2ms; MeA; **g**). The connection of HyA with the Pa area beyond the interventricular foramen appears clearly in **c**–**e**. For abbreviations, see list. Scale bars represent 350 µm (**a**–**e**) and 150 µm (**f**, **g**)

**Fig. 6:**
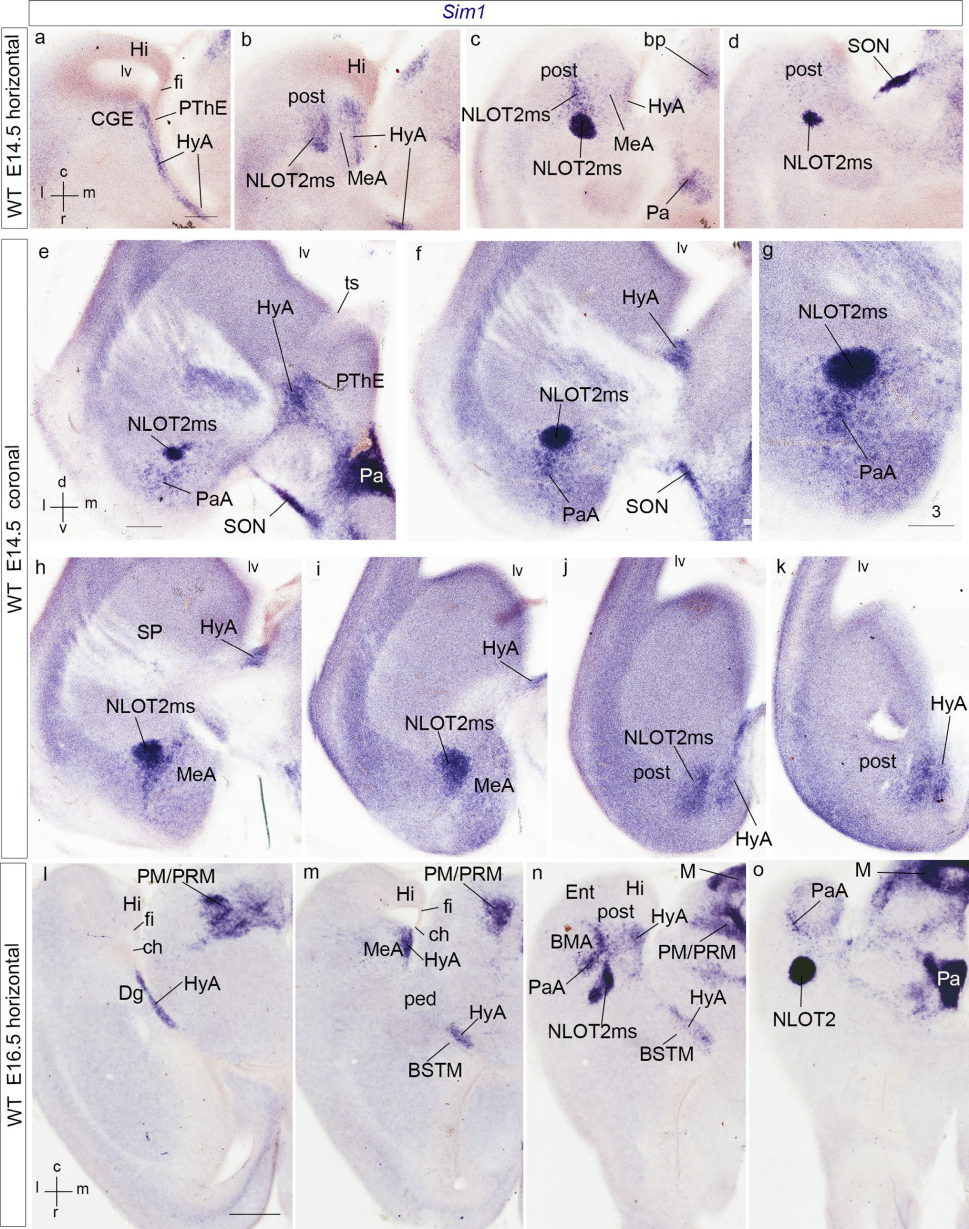
Progress of *Sim1*-expressing NLOT2ms in horizontal and coronal sections, relative to the HyA, at E14.5 and E16.5. The spatial orientation appears at the bottom left-hand corner of **a**, **e**, **l**. **a**–**d** This dorsoventral horizontally sectioned series shows dorsally the HyA stretching along the terminal sulcus (HyA; **a**), its arrival at the posterior pallial amygdala, medially to the caudal end of MeA (HyA; MeA; post; **b**), and the lateral emergence of the NLOT2ms, with a rounded and cell-dense advance head (NLOT2ms; **b**–**d**). **e**–**k** This anteroposterior coronally sectioned series shows rostrally the rounded advance head of the NLOT2ms, as well as the connection between HyA and the Pa/SON area (NLOT2ms; HyA; Pa; **e**; see also PThE and terminal sulcus—ts—separating it from ganglionic eminences). Sections that are more caudal follow the NLOT2ms backwards into its less dense stalk at the posterior amygdala, laterally to MeA, and continue showing the advance of HyA along the terminal sulcus (NLOT2ms; post; MeA; **f**–**i**;** g** is a magnified detail of **f**). The last two sections illustrate how the NLOT2ms stalk at the *posterior* amygdala connects with the HyA under the end of the MeA (HLOT2ms; HyA; post; **j**, **k**). Note also the rostral ventralward dissociation from the NLOT2ms of some labeled cells, which correspond to the para-anterior group (PaA; **e**–**g**). **l**–**o** At E16.5 the relative proportions of different telencephalic cell masses have changed, but a dorsoventral horizontal section series still shows the HyA stretching along the terminal sulcus next to the diagonal domain (HyA; Dg; **l**–**n**) and reaching the posterior amygdala medially to MeA (HyA; post; MeA; **m–o**). The NLOT2ms emerges laterally to MeA and produces the PaA cell group that forms a medial shell for the BMA, as well as the advancing stream ending in its rounded head (NLOT2ms; PaA; BMA; **n**, **o**). For abbreviations, see list. Scale bars represent 200 µm (**a**–**d**), 200 µm (**e**, **f**, **h–k**), 150 µm (**g**), 400 µm (**l**–**o**)

To explore further development of the *Sim1-*expressing cells of the HyA corridor, we mapped this gene in horizontal and coronal sections at stages E14.5 and E16.5 (Figs. 5, 6).

At E14.5, *Sim1* signal is still present at the supracapsular periventricular stratum of the HyA corridor. We visualize it in dorsal horizontal sections and intermediate coronal sections along the bottom of the terminal sulcus and next to the subpallial MGE and CGE bulges. The HyA lies orthogonally supracapsular relative to the internal capsule or peduncle, which courses through its underlying intermediate stratum before entering the subpallium (HyA; MGE; CGE; ped; Figs. 5a, f, 6a, b, f, h, i). In lower horizontal sections and rostral coronal sections, the topologically ventral end of the *Sim1*-positive HyA bends rostro-medially into the similarly labeled hypothalamic Pa (HyA; Pa; Figs. 5b–d, 6a–c, e). It also reaches caudally the posterior pallial amygdala, past the MeA, as a thin radially stretched domain reaching the pial surface (HyA; Figs. 5b, c, g, 6b, j, k). More rostrally, its para-amygdalar position still appears intercalated between the subpallial CGE (or MeA) territory, lateral to it, and the evaginated telencephalic PThE portion (PThEt), medially. The latter also separates the HyA corridor from caudal hippocampal structures (note the chorioidal fissure ends slightly above this level; ch; HyA; CGE/MeA; post; Hi; PThEt; Figs. 5a–c, f, 6a, b).

Starting at E13.5, a substantial mass of periventricular *Sim1*-positive cells, interpreted as hypothalamic HyA-vehiculated *Sim1* cells, incorporate into the incipient amygdalar NLOT2 migration stream. The latter appears periventricularly at the *posterior* amygdalar pallial domain, laterally to the caudal end of the CGE/MeA (post; Figs. 3b, c, 5g). At E14.5, this population extends rostralwards through the pallial amygdala as a distinct aggregated cell stream with a more dispersed trail component, coursing parallel and adjacent to the amygdalar pallio-subpallial boundary (NLOT2ms; Figs. 5b–e, 6b–k). This *Sim1* stream clearly corresponds in shape and topography with the *Tbr1-*expressing NLOT2 or CAS migrating stream described by Remedios et al. (2007) (Fig. 9a, b, d, e; see also coincidence with other relevant NLOT2ms markers such as *Zic2* and *Neurod1*, below).

Consistently with results at later stages, we straightforwardly called this structure the *NLOT2 migratory stream* (NLOT2ms). We use minimally the alternative term ‘caudal amygdaloid stream’ or CAS (Remedios et al. 2007), which at first sight seems perfectly appropriate, because the latter was misleadingly defined as originated in ‘caudal’ dorsal pallium, a point of discrepancy and possible reader confusion we will deal with below. We observed that this E14.5 formation extends from the periventricular stratum of the amygdalar *posterior* radial unit (prospective AHi), which typically reaches with its *rostromedial subdivision* the medial brain wall caudally to the MeA, to a more rostral domain within the amygdalar pallial mantle. We estimate that the latter locus lies close to the BLA primordium of the *basolateral* radial unit (post; NLOT2ms; A; Fig. 5b–e, g; see below comparisons with Tbr1 immunoreaction to establish pallial amygdalar territories). The stream of *Sim1*-positive cells does not reach yet at E14.5 the final NLOT2 locus within the anterior amygdala (AA), being still restricted to pallial amygdala.

At stage E16.5, the *Sim1*-expressing periventricular HyA corridor appears stretched into a relatively thin supracapsular band. This is presumably due to rostrocaudal morphogenetic growth and increased torsion of the hemisphere. Nevertheless, the HyA retains its previous topologic position along the bottom of the terminal sulcus. It still courses next to subpallial primordia of the diagonal domain and the bed nucleus of stria terminalis that similarly arch back into the amygdalar region (HyA; Dg; BSTM; Fig. 6l–n). These subpallial structures differentially present calbindin immunoreaction and *Lhx6/Sst* ISH reaction (not shown; Allen Developing Mouse Brain Atlas; García-López et al. 2008; Medina and Abellán 2012; Puelles et al. 2016a). Other *Sim1*-expressing cells, either sorting out ventralwards out of the dense NLOT2ms, or coming subcapsularly from its dispersed trail elements, form a tenuous medial shell around the amygdalar BMA nucleus, a component of the *anterior* pallial radial unit. We called this divergent population the *para-anterior cell group* (PaA; Fig. 6n, o; see also PaA in Figs. 13h–j, 14b). Postnatally, the PaA cells still lie caudally to the NLOT2 and medially to the BMA (Allen Developing Mouse Brain Atlas). At E16.5, most *Sim1*-labeled cells of the NLOT2ms have separated from the *posterior* amygdalar unit ventricle, and some pioneering ones may be reaching already the incipient NLOT2 nucleus, after crossing the pallio-subpallial boundary (NLOT2ms; NLOT2; Fig. 6n, o, compare Figs. 11k–m, 13g).

### Description of the Tbr1-expressing NLOT2 migratory stream in the context of ***Sim1*** expression in the telencephalic vesicle

**Fig. 7:**
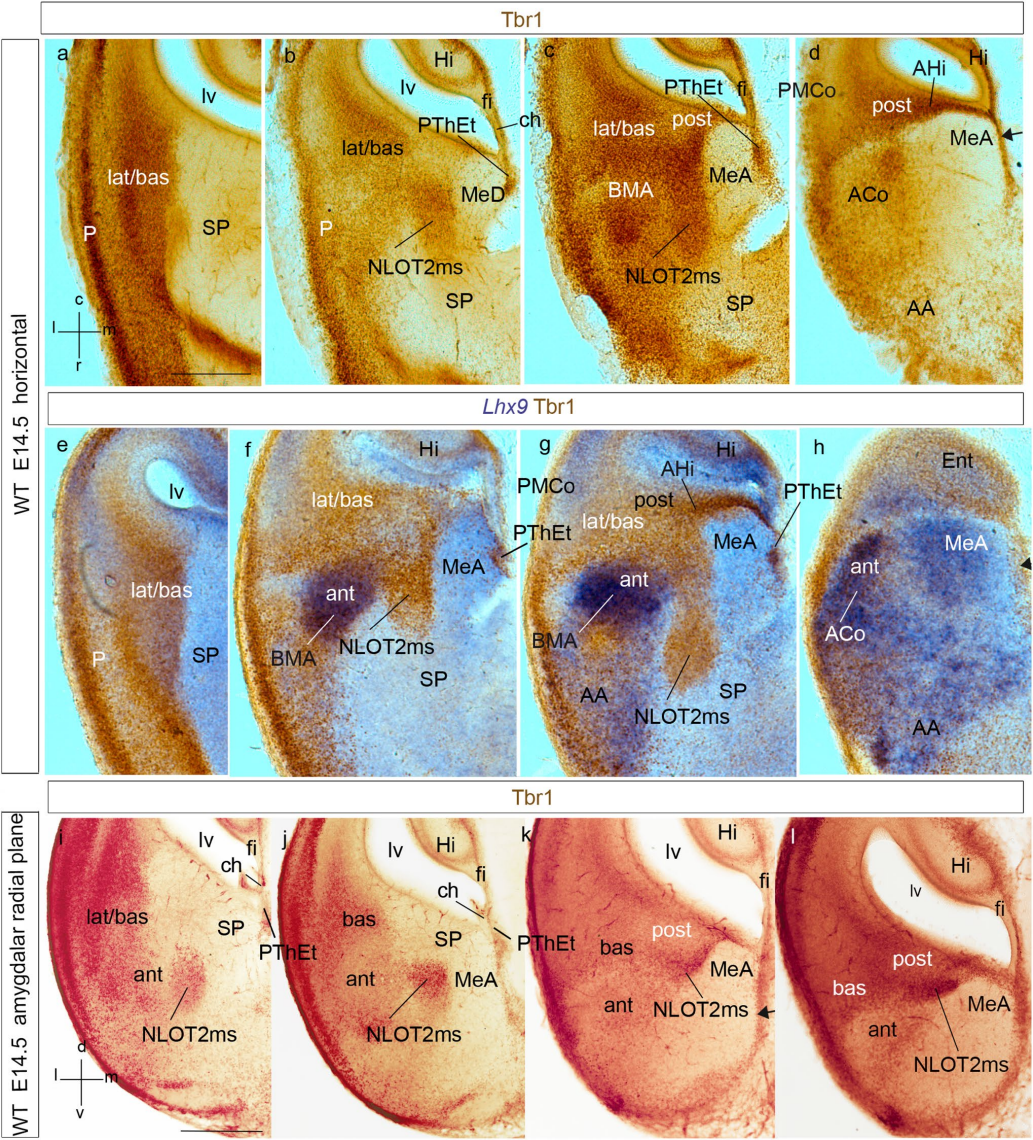
Horizontal sections at E14.5, illustrating relationships of the Tbr1-immunoreacted NLOT2ms—partly counterstained with *Lhx9* signal—in horizontal and radial amygdalar section planes. The spatial orientation appears at the bottom left-hand corner of **a**, **i**. **a**–**d** Brown Tbr1 pallial immunoreaction (P) contrasts with the unlabeled subpallium (SP). The dorsoventral series shows some pallial amygdalar subdivisions (lat/bas, BMA, ACo, post) jointly with the advancing NLOT2ms at the pallio-subpallial boundary (lat/bas; BMA; post; NLOT2ms; **a**–**d**). Note relationship with the subpallial MeA (MeA; **b**–**d**). At the medial brain surface, the marginal migrating stream of the PThEt appears also Tbr1-labeled (PThEt; black arrow; **b**–**d**). **e–h** Similar Tbr1-immunoreacted dorsoventral horizontal series, counterstained with *Lhx9* blue in situ signal, to compare with NLOT2ms. Major *Lhx9* labeling appears at derivatives of the *anterior* amygdalar unit, the BMA and ACo nuclei (ant; BMA; ACo; NLOT2ms; **f–h**). Tangentially migrated *Lhx9*-positive cells from the *anterior* pallial amygdala appear rostrally to the NLOT2ms at the anterior subpallial amygdala (AA) and medially at the MeA (AA; MeA; **h**). **i**–**l** Anteroposterior series of radial amygdalar sections at E14.5 showing the NLOT2ms cut orthogonally to horizontal sections in **c**, **g**. At E14.5, the Tbr1-labeled migration has advanced mainly through its pallial path phase, bringing the stream close to the anterior radial unit (with weak Tbr1 reaction), where it starts to enter the subpallium (SP); note the caudalmost dense NLOT2ms lies at the pallial side of the pallio-subpallial boundary (NLOT2ms; ant; post; SP; MeA; **i**–**l**). Note also that where the NLOT2ms starts to penetrate the subpallium, it is also covered dorsally by subpallial formations, probably the central amygdala (NLOT2ms; SP; **i**, **j**; compare Fig. 8b). For abbreviations, see list. Scale bars represent 300 µm

**Fig. 8:**
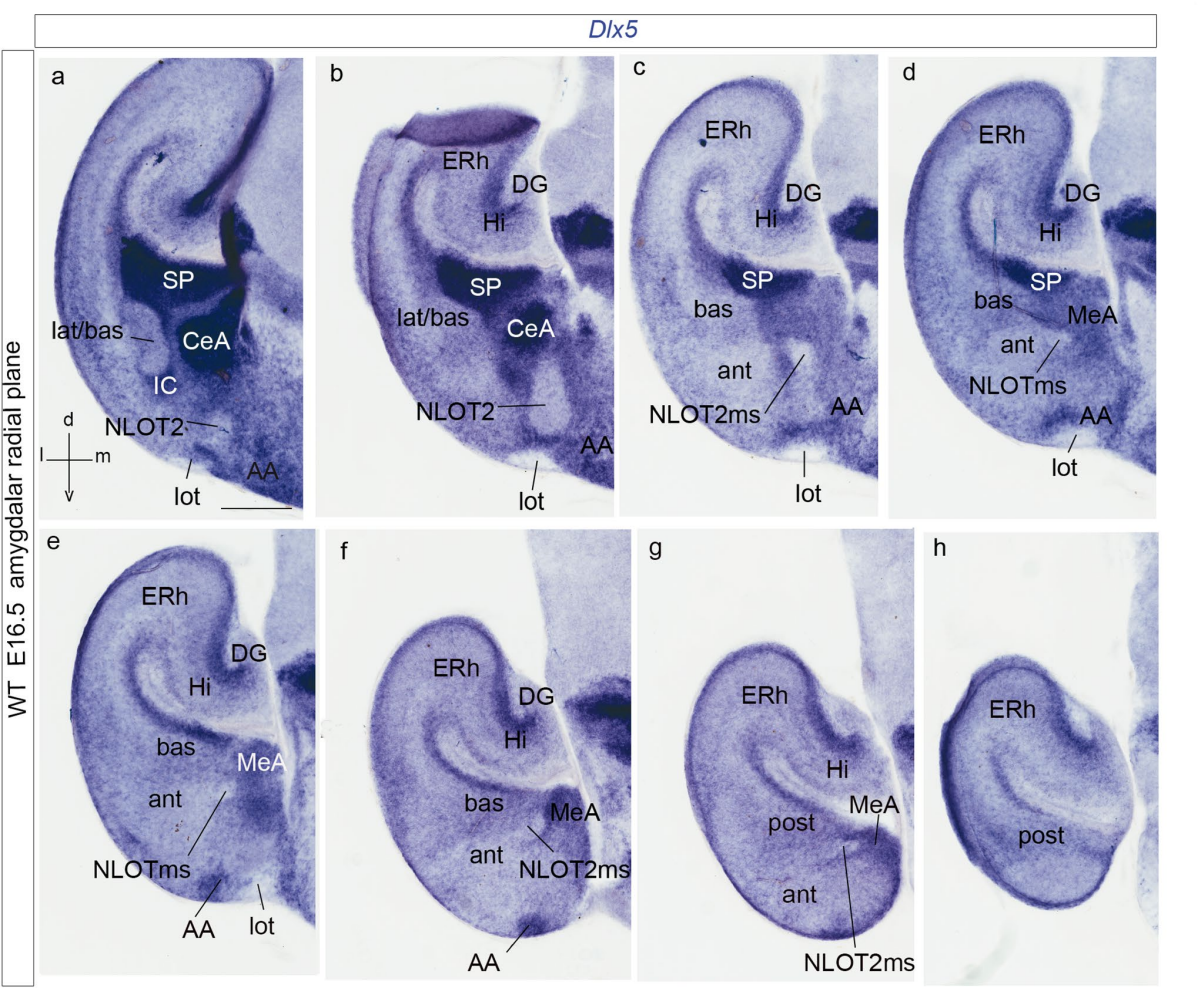
Rostrocaudal series of radial amygdalar sections showing *Dlx5 *in situ labeling of the subpallium at E16.5, to visualize the radial penetration of this territory by the negative NLOT2ms. **a**–**d** Sections showing periventricular and intermediate subpallium strata (SP; CeA), as well as the less dense superficial subpallial stratum penetrated by the migrated NLOT2 (partly under the CeA, where *Six3*-positive cells were found –see text-, and on top of the AA and the lateral olfactory tract; CeA; lot; **a**, **b**; AA; lot; **b**–**f**). The migrating NLOT2ms can be followed back into the posterior amygdala, laterally to the MeA, by its total lack of *Dlx5* signal (NLOT2ms; post; MeA; **c**–**g**); note also the primordium of the subpallial intercalated cell mass (IC), forming a rostral capsule to the rostrally advancing knee of the amygdalar laterobasal pallial units (IC; lat/bas; **a**, **b**). Note also the constant position caudal to the pallial amygdala of the hippocampal and entorhinal cortex (Hi; ERh; **b**–**h**). For abbreviations, see list. Scale bar represents 300 µm

**Fig. 9:**
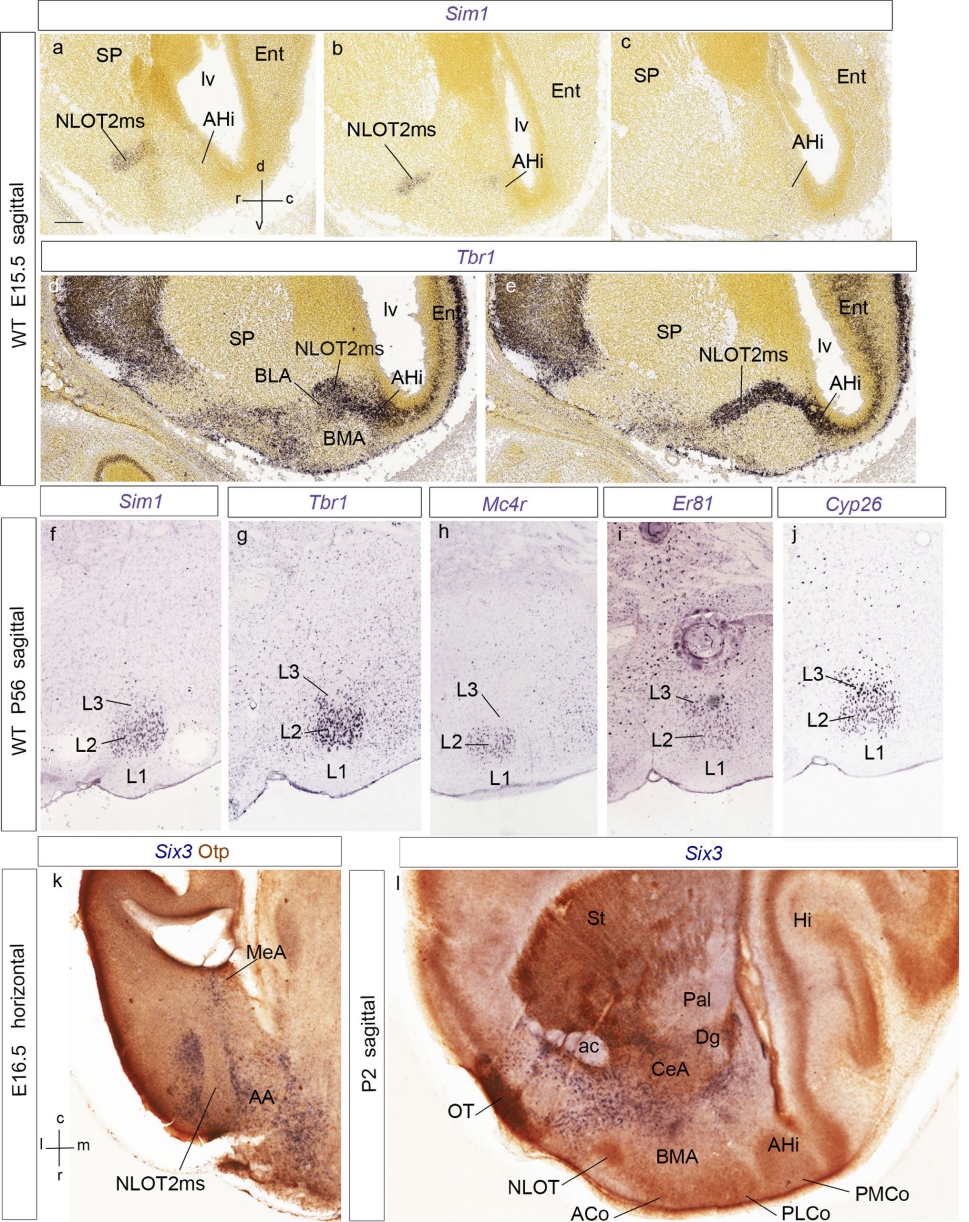
Panels illustrating sagittal sections of the *Sim1*- and Tbr1-labeled NLOT2ms at E15.5 (**a**–**e**), adult (P56) sagittal aspect of the NLOT labeled with *Sim1*, *Tbr1*, *Mc4r*, *Er81* and *Cyp26* (**f**–**j**), and horizontal (E16.5) and sagittal (P2) images of *Six3*-labeling of subpallial centro-amygdalar cells dorsally to the NLOT2ms (**k**, **l**). The spatial orientation appears at the bottom left-hand corner of **a**, **k**. The *Sim1* and *Tbr1* transcripts seen in E15.5 sagittal sections through the NLOT2ms clearly correspond topographically (NLOT2ms; **a**–**e**). Note relationship of amygdalar AHi (posterior amygdalar unit) with ventromedial ERh cortex, the latter being devoid of *Sim1* signal (AHi; Ent; **a**–**e**). Note also change of direction of NLOT2ms (pallial phase into subpallial phase) at the neighborhood of BMA (**d**). **f**–**j** These sections show that *Sim1*, *Tbr1* and *Mc4r* mainly label the NLOT2 layer (L2), whereas *Er81* and *Cyp26* label mainly L3, with fainter signal at L2. **k**, **l** These sections illustrate an intimate dorsal relationship of the NLOT2ms at the subpallial phase of its course with* Six3*-positive cells belonging to the CeA nucleus and similar superficial stratum cells extending into the olfactory tubercle (OT). At E16.5, Otp counterstaining (red reaction) shows a positive HyA-migrated cell group at the MeA, next to the chorioidal tela insertion (**k**). For abbreviations, see list. Scale bars represent 400 µm

Since Tbr1 signal is a general pallial marker in the telencephalic mantle it labels the pallial amygdalar NLOT2 migratory stream as well as the definitive NLOT2 itself (layers 2 and 3; Fig. 9g; Remedios et al. 2007). We studied this marker by immunoreaction in horizontal sections (comparable to our *Sim1* material at stage E14.5; Fig. 7a–h), as well as in the amygdalar radial section plane (Fig. 7i–l; Garcia-Calero et al. 2020). We hybridized in situ some sections of the horizontal series with the *Lhx9* probe, which selectively labels the BMA and ACo nuclei of the *anterior* amygdalar radial unit, as well as the neighboring ventral subdivision of medial amygdala and the anterior amygdala (Garcia-Calero et al. 2020; Garcia-Calero and Puelles 2021). We also examined the distribution of the subpallial marker *Dlx5*, whose signal is absent from the NLOT2 and its migration stream (Fig. 8a–h; Garcia-Calero et al. 2020).

This partly counterstained Tbr1 material shows a sharp contrast between the Tbr1-positive pallial amygdalar region and the Tbr1-negative medial amygdala and neighboring amygdalar subpallium (note the CeA and AA domains are distinctly *Dlx5* positive areas, whereas MeA shows a weaker *Dlx5* signal, possibly due to its diagonal nature; Puelles et al. 2016a; Fig. 8a–d). The pallial domain includes at dorsal section levels the *lateral* and *basal* radial units (P; SP; lat/bas; Fig. 7a–c, e; see also Fig. 7i–l in the radial plane), representing the primordia of the prospective lateral (L) and basolateral (BLA/BLP/BLI) nuclei (Garcia-Calero et al. 2020). Underlying horizontal sections also intersect the *Lhx9*-expressing *anterior* pallial radial unit (ant; BMA/ACo; Fig. 7f–h), which generally shows a low level of Tbr1 signal (Tole et al. 2005), in contrast to the *posterior* radial unit, whose periventricular AHi formation is strongly Tbr1-positive (post; AHi; Fig. 7d, g; see also Fig. 7k, l in the radial plane).

The evaginated PThE (PThEt) and its marginal migratory stream are observed as a superficial patch of Tbr1-positive cells limiting medially the medial amygdala, i.e., apparently covering superficially the MeA, though they are separated in fact by the compacted end of the terminal sulcus (PThEt; MeA; Fig. 7b, c, f, g, i, j; black arrow in Fig. 7d, k). The PThEt clearly reaches the caudal hippocampal formation beyond the caudal end of the chorioidal fissure and its fimbrial attachment (PThEt; ch; fi; Hi; Fig. 7b–d, f, g, i–l). In contrast, MeA is distinctly a Tbr1-negative domain (MeD; MeA; Fig. 7b–d, f–h, j–l).

As regards its apparent origin, the Tbr1-positive and *Dlx5*-negative NLOT2 *migratory stream* (NLOT2ms) is clearly continuous at E14.5 with the *posterior* pallial amygdalar domain where *Sim1*-expressing cells accumulate after E13.5. Once the Tbr1-positive NLOT2ms approaches the pallio-subpallial boundary, it appears intercalated between the *Lhx9*-positive and weakly Tbr1-expressing *anterior* radial unit (BMA nucleus) and the non-pallial MeA (NLOT2ms; ant; BMA; MeA; Figs. 7b, c, f, g, i–k, 8a–g). Rostrally, the *Dlx5*-negative head of the migrating stream approaches the *Dlx5*-expressing anterior amygdalar area (AA), which was also previously invaded tangentially by *Lhx9*/*Lhx2*-positive cells of the *anterior* radial unit (AA; compare *Lhx9* in Fig. 7h and *Dlx5* in Fig. 8a–d; Garcia-Calero et al. 2020; Garcia-Calero and Puelles 2021). The dorsal aspect of the NLOT2ms at E16.5 appears covered intimately by *Six3*-positive neurons apparently related to the CeA nucleus (Fig. 9k).

There may exist both lateral BLA-related and posteromedial AHi-related roots of the NLOTms (Fig. 7c), though the posteromedial root arising at the AHi clearly is the main one, and is the one that incorporates the *Sim1*-expressing population (see schema in Fig. 15b).

The posteromedial NLOT2ms root links the periventricular stratum of the AHi area, ascribed to the *posterior* pallial radial unit, to the main NLOT2ms (NLOT2ms; Fig. 7c, f, g, j–l; Garcia-Calero et al. 2020). Our material clearly shows that the NLOT2ms coming out of this root first advances inside the pallial amygdala, next to the boundary of the posterior pallial amygdalar region with the MeA (NLOT2ms; post; AHi; MeA; Figs. 7c, k, l, 8f, g).

At E14.5, the advancing rounded tip of the NLOT2ms has already progressed up to the BLA and BMA pallial amygdalar nuclear primordia (NLOT2ms; lat/bas; ant; BMA; Figs. 7b, c, f, g, i, j, 8c–e), where the ancillary lateral root of the migration may be added (Fig. 7c, f, g, k; see below). The stream crosses immediately thereafter obliquely the pallio-subpallial boundary, as indicated by global comparison of pallial Tbr1 immunoreaction with *Dlx5* subpallial signal (Figs. 7, 8). Note the rostral NLOT2ms and the incipient NLOT2 primordium within AA are separated laterally from the standard pallium by a band of Tbr1-negative and *Dlx5/Six3*-positive subpallial cells (Figs. 7f, g, 8b, c, 9k). This band disappears more caudally, where the stream is restricted to amygdalar pallium (Figs. 7b, c, f, g, k, l, 8d–g).

Marked partial continuity of the pallial BLA nucleus and the NLOT2ms cell mass suggests that a secondary *lateral* pallial root of the NLOT2ms possibly arises at the *basolateral* radial unit (Fig. 7c, f, g, k). The interaction would occur at the locus where the BLA later displays a subpopulation that departs from the standard radial disposition of *basolateral* radial unit derivatives, and advances tangentially in lateromedial direction, forming a cap over the BMA nucleus (Garcia-Calero et al. 2020). This aberrant cap population ends forming a medially prominent ‘horn’ of the BLA nucleus, which protrudes into the MeA/AA, and apparently follows partially the transient NLOT2ms rostralwards as it passes by, possibly contributing cells to it (Garcia-Calero et al. 2020). It is however impossible to assess descriptively whether basolateral Tbr1-positive cells indeed pass from the BLA cap and horn into the NLOT2ms, or just stop at the horn. The fact that various BLA markers (including AChE and TH activity; Garcia-Calero et al. 2020) do not appear in the NLOT bears against the hypothesis. Perhaps the BLA horn cells are only *partially attracted* into the passing NLOT2ms, without further consequences. Remarkably, though, the same BLA horn locus appears related in the adult to a parallel (and molecularly distinct) *amygdalo-olfactory migratory cell stream* population (AOS), which extends from the horn all the way into the NLOT, accumulating there into layer 3 and a peripheral shell (Fig. 9i, j; Garcia-Calero et al. 2020).

The *Sim1*-expressing cells penetrate the NLOT2ms close to the caudal tip of the hypothalamo-amygdalar corridor, apparently passing around the caudal end of the MeA (Figs. 5b, c, g, 6b, j, k, 7l, 8g; see schema in Fig. 15b). Sagittal sections found at the Allen Developing Mouse Brain Atlas, illustrating both *Sim1* and *Tbr1* markers in E15.5 embryos, corroborate present results (Fig. 9a–e); note AHi expression of *Sim1* decreases substantially at E15.5 (Fig. 9c).

On the other hand, the Tbr1-positive pallial neurons which principally build the NLOT2 mainly seem to originate at the same *posterior* amygdalar locus (AHi) invaded by the *Sim1* cells. There is a first phase of pallial intra-amygdalar migration, which runs orthogonal to local radial glia (Remedios et al. 2007; present results). After a decision point next to BLA and BMA nuclei, the migratory stream changes directions, perforates the boundary between pallial amygdala and subpallial amygdala, and proceeds in a descending radial course to form the NLOT primordium within AA (schemata in Fig. 15b, d). The adult NLOT shows *Sim1*, *Tbr1*, and *Mc4r* transcripts at its layer 2 (Fig. 9f–h), whereas the markers *Cyp26* and *Er81* label in addition some layer 3 components, which relate to the AOS shell and trail populations (Fig. 9i, j; Garcia-Calero et al. 2020).

### Other relevant gene patterns: *Zic2, NeuroD1, NeuroD2, NeuroD6, Lhx2, Emx1, Six3*.

**Fig. 10:**
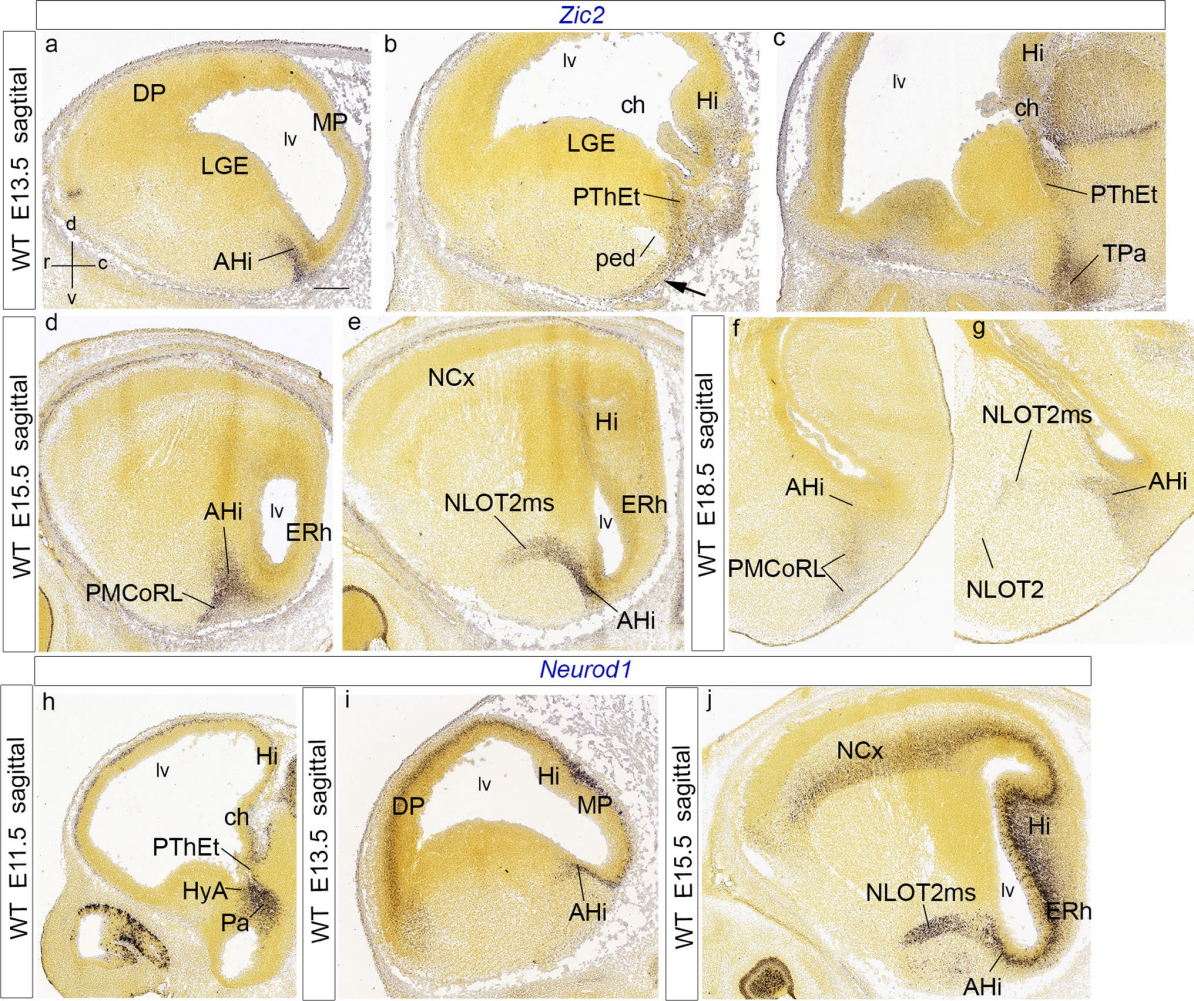
Sagittal sections illustrating amygdalar *Zic2* transcripts at E13.5, E15.5 and E18.5 (**a**–**g**), jointly with Neurod1 transcripts at E11.5, E13.5 and E15.5 (**h**–**j**). The spatial orientation appears at the bottom left-hand corner of **a**. At E13.5, Zic2 signal appears at the intermediate AHi mantle (**a**), as well as at the PThEt and its marginal migration stream passing medially caudal to the peduncle (PThEt; ped; black arrow; **b**, **c**), as well as the terminal part of the paraventricular area (TPa; **c**). At E15.5, the AHi *Zic2* signal continues labeling the AHi, and, particularly the full radial extent of its rostrolateral subdivision (AHi; PMCoRL; **d**), as well as the NLOT2ms (**e**; note neighboring ERh and Hi cortex). At E18.5, the *Zic2* labeling is less marked, but persists at some of the places seen before (AHi, PMCoRL, NLOT2ms, but not at the NLOT2; **f**, **g**). **h**–**j** *Neurod1* transcripts appear at the paraventricular hypothalamic area (Pa) and HyA at E11.5 (Pa; HyA; **h**). At E13.5, *Neurod1* signal appears at the AHi mantle, deep to the level labeled with *Zic2* (AHi; compare **a** with **i**; see also the relative positions of dorsal and medial pallium, DP, MP). At E15.5, Neurod1 signal clearly identifies the NLOT2ms and AHi periventricular stratum, the latter connected under the end of the lateral ventricle (lv) with ERh and Hi cortex derived from medial pallium (NLOT2ms; AHi; ERh; Hi; **j**). For abbreviations, see list. Scale bar represents 400 µm

The transcription factor *Zic2* is of interest, since its expression characterizes the initial phase of the NLOT2ms within pallial amygdala, but not the subsequent subpallial phase, or the definitive NLOT nucleus; moreover, lack of function of this gene leads to loss of the NLOT nucleus (Murillo et al. 2015). Our analysis of this pattern uses the Allen Developing Mouse Brain Atlas repository. At E13.5, amygdalar *Zic2* expression is weak at the pallial AHi ventricular zone and strong at its incipient mantle (AHi; Fig. 10a). The latter’s medial end lies close, but separate, from a marginal stream of *Zic2*-expressing cells which spreads out of the PThEt mantle, arching superficially to the cerebral peduncle (black arrow; PThEt; ped; Fig. 10b; see Alonso et al. 2020a,b). There is also *Zic2* expression at the terminal portion of the hypothalamic Pa area (TPa; Fig. 10c). At E15.5 *Zic2* appears expressed in a caudo-rostral gradient along the NLOT2ms, which reaches the locus of confluence of the two NLOT2ms roots next to BLA. The *Zic2*-labelled NLOT2ms distinctly originates from the strongly positive AHi mantle. Identification of the latter is certified further by distinct labeling of its characteristic *rostrolateral* radial subdivision (see Garcia-Calero et al. 2020), which typically ends superficially at the lateral aspect of the PLCo, rather than within the PMCo, like other parts of AHi (NLOT2ms; AHi; PMCoRL; Fig. 10d, e). No connection was visible between the separate labelled mantle layers of AHi and PThEt (not shown). At E18.5, weak *Zic2* signal remains at the AHi mantle and its related rostrolateral subdivision, but has practically disappeared from the remnant of the NLOT2ms, and the NLOT2 proper is completely negative (AHi; PMCoRL; NLOT2ms; NLOT; Fig. 10f, g).

**Fig. 11:**
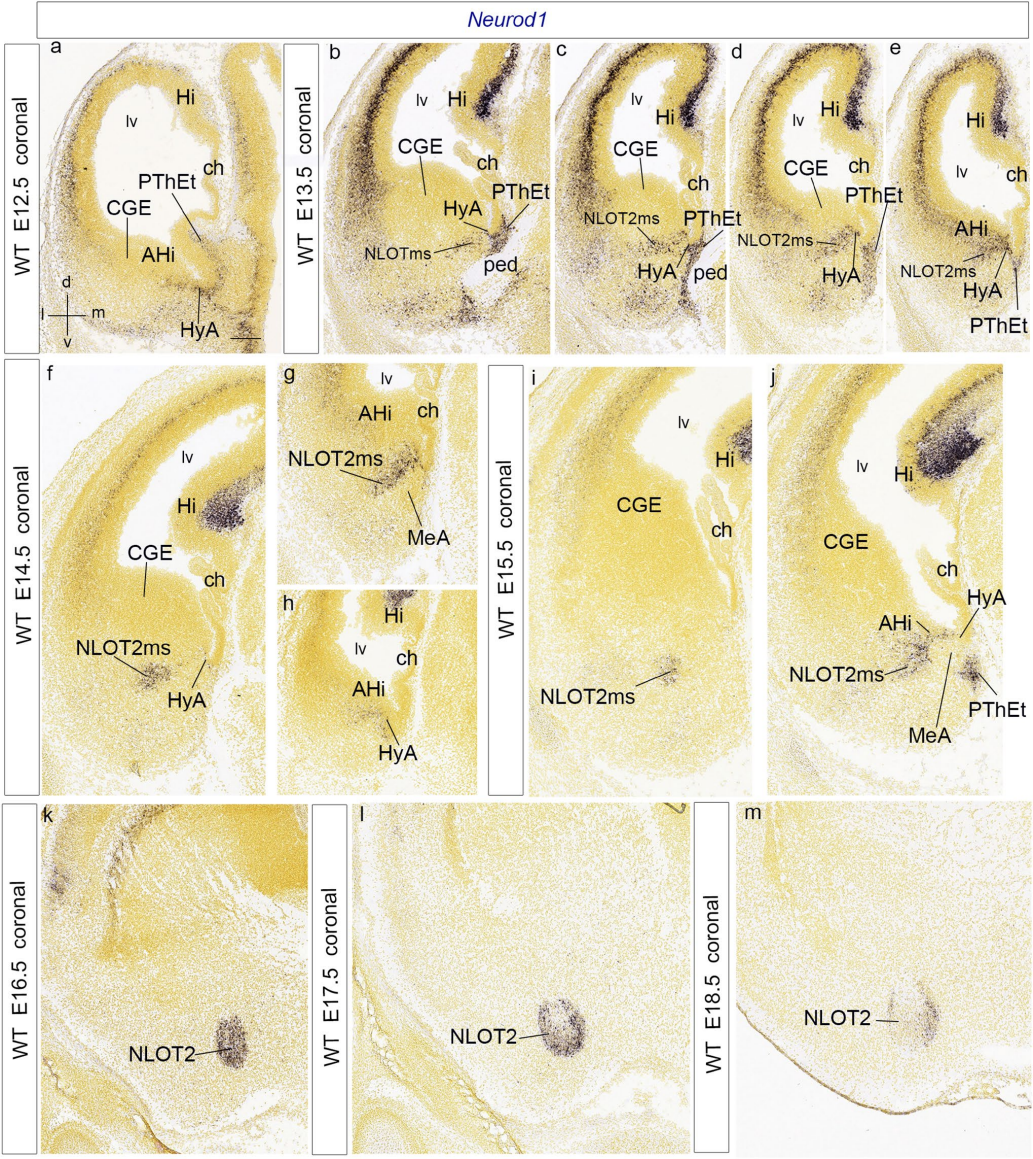
Anteroposteriorly ordered coronal sections illustrating *Neurod1* amygdalar expression at E12.5, E13.5, E14.5, E15.5, E16.5, E17.5 and E18.5 (note coronal sections cut obliquely the NLOT2ms, as well as the whole pallial amygdala). The spatial orientation appears at the bottom left-hand corner of **a**. At E12.5, *Neurod1* signal appears at the Pa, HyA, AHi and PThEt (**a**). At E13.5, the signal within the PThEt mantle expands medioventrally into its marginal migration stream at the back of the peduncle. Medially to HyA (PThEt; ped; HyA; **b**–**e**). The labeled NLOT2ms appears cut obliquely in a more lateral position, as a positive patch stretching back to the AHi domain (NLOT2ms; AHi; **b**–**e**). A similar anteroposterior sequence through NLOT2ms appears at E14.5 (NLOT2ms; AHi; **f–h**). At E15.5, the Neurod1-labeled NLOT2ms appears still connected caudally to the AHi (NLOT2ms; AHi; **i**, **j**); note also the labeled medial superficial marginal stream of the PThEt at (**j**). At more advanced stages, the definitive NLOT nucleus starts to conform, with *Neurod1* signal increasingly restricted to its layer 2 (NLOT2; **k**–**m**). For abbreviations, see list. Scale bar represents 400 µm

We studied *Neurod1* transcripts in sagittal and coronal sections (Figs. 10h–j, 11; data from the Allen Developing Mouse Brain Atlas). Signal was already present at E11.5 along an incipient mantle continuum, which communicates the PThE and the rostrally adjacent hypothalamic Pa area with the pallial amygdala mantle (the latter along the HyA corridor; Fig. 10h). This triple relationship appears clearly in coronal sections at E12.5 (AHi; PThEt; HyA; Fig. 11a). The amygdalar pallial mantle shows at E13.5 a dense *Neurod1* labelling of the *Zic2*-negative later-born AHi periventricular stratum, possibly suggesting later postmitotic expression than *Zic2* cells (AHi; Fig. 10i; compare with Fig. 10a). The pattern in coronal sections is reminiscent of the *Sim1* pattern at this stage, with *posterior* amygdalar unit elements labeled lateral to unlabeled MeA and labeled HyA and PThEt (HyA; AHi; PThEt; Fig. 11b–e). We believe that its expanded rostral portion is the beginning of the NLOTms (NLOT2ms; Fig. 11b–e). A topographically corresponding labeled cell patch stretches even more rostralward in a similar position at E14.5, partly detached now from the AHi periventricular stratum (NLOT2ms; AHi; Fig. 11f–h). The PThEt mantle is massively labelled with *Neurod1* at E13.5 and shows a marked tangentially migrated stream of *Neurod1*-positive cells passing outside the cerebral peduncle (PThEt; ped; Fig. 11b–d); this stream is the same which was revealed by *Zic2* signal, seen now more favorably. Notably, there is also labeling of the deep layers of the allocortical and neocortical pallium (Figs. 10i, 11b–f).

At E15.5 the arc-shaped *Neurod1*-positive NLOT2ms is recognized in sagittal section (Fig. 10j) as described by Remedios et al. (2007). Interestingly, at E15.5 the migrating stream still partly connects caudally with the periventricular AHi, where it is separated by a distinct negative gap from other labeling seen medial to the MeA, which may be ascribed to the HyA remnant and the marginal PThEt migration mentioned above (NLOT2ms; AHi; MeA; HyA; PThEt; Fig. 11g, h, j). At E16.5 the target NLOT2 locus starts to be reached by the *Neurod1* signal. The rostral rounded and larger end of the NLOT2ms is now the strongest labelled part (Fig. 11k); a tenuously labeled tail of the migration stream is still visible, corresponding to the PaA population described above (not shown). The NLOT2ms has lost its caudal connection with the AHi source at this stage (not shown). There remains signal at the deep cortical stratum (unmarked in Fig. 11k). At E17.5 the *Neurod1*-labelled cells start to aggregate and compact into the prospective NLOT2, leaving prospective layer 3 less populated; most positive cells adopt a peripheral shell-like position (NLOT2; Fig. 11l). At E18.5 most labelled NLOT cell are concentrated at the cited shell-like peripheral configuration, appearing as rostral and caudal shells in sagittal sections and as medial and lateral shells in coronal sections (NLOT2; Fig. 11m).

**Fig. 12:**
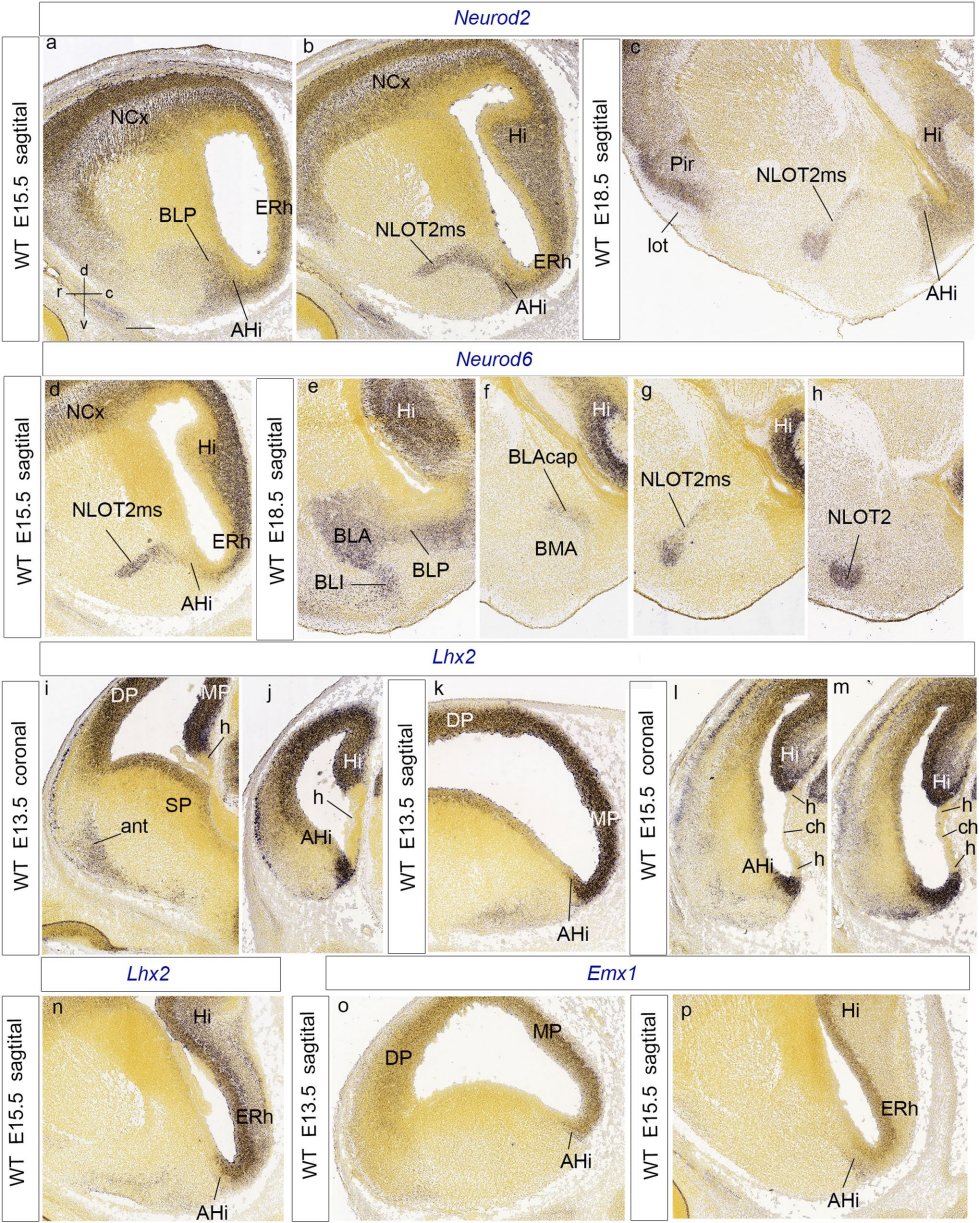
Lateromedially ordered sagittal (or coronal) sections at various stages, showing amygdalar and cortical expression of *Neurod2*, *Neurod6*, *Lhx2* and *Emx1*. **a**–**c** *Neurod2* signal labels the BLP, AHi and NLOT2ms, jointly with most mediopallial and dorsopallial cortical areas at E15.5 (**a**, **b**). At E18.5, the signal is visible at the olfactory cortex (Pir), NLOT2ms, AHi and neighboring Hi cortex (Pir; lot; NLOT2ms; AHi; Hi; **c**). **d**–**h** *Neurod6* signal characterizes the typical NLOT2ms at E15.5, but shows scarce signal at the AHi (**d**). At E18.5, lateral sections show *Neurod6*-labeled most derivatives of the *laterobasal* amygdalar radial unit, namely the nuclei BLP, BLA, and BLI (**e**). The BLA signal extends medialwards into the so-called BLA cap, which overlies the unlabeled BMA nucleus (BLAcap; BMA; **f**). Medial to this, theoretically passing through the BLA medial horn (not shown), labeling continues at the NLOT2ms and final NLOT2 nucleus (**g**, **h**). Note neighboring labeled Hi (**e–g**). *Lhx2* transcripts in coronal sections show rostrally weak signal at the anterior amygdalar radial unit (ant), as well as labeling at the subpallial ventricular zone (SP), the lateral dorsal pallium (DP), and the medial hippocampal pallium (MP), with unlabeled cortical hem (h) (ant, SP; DP, MP, h; **i**). More caudally, coinciding with the elongated end of the hem (h), strong labeling appears at the AHi mantle and ventricular zone (AHi; h; Hi; **j**). This pattern is observed also in a sagittal section at E13.5 (AHi; MP; DP; **k**). Images of *Lhx2*-labeled AHi ventricular zone and mantle next to mediopallial formations (and similar to those at **j**, **k**) appear also at E15.5 (AHi; h; ch; Hi, ERh; **l**–**n**). **o**, **p** *Emx1* signal appears at E13.5 and E15.5 at the AHi ventricular zone and mantle, caudally related medial pallium derivatives (MP; ERh; Hi), and, less strongly, and in a rostrally decreasing gradient, at the dorsal pallium (DP). For abbreviations, see list. Scale bar represents 400 µm

*Neurod2* shows at E15.5 a rather diffuse and graded expression pattern in the amygdalar BLP and AHi nuclear primordia, as well as in the hippocampal/entorhinal mantle, with strongest signal at the AHi and the related NLOT2ms, homogeneously labelled throughout (NLOT2ms; BLP; AHi; Fig. 12a, b). At E18.5, there is distinct labeling at the NLOT primordium, as well as at remnants of the migration stream, and at the now discontinuous AHi mantle and cortical pallium mantle (NLOTms; AHi: Fig. 12c). The NLOT layers are distinct at P4; a few positive cells disperse superficially to NLOT2, possibly within NLOT1; others remain associated to NLOT3 (not shown).

*Neurod6* (*Math2*) transcripts appear at the deep periventricular amygdalar pallium at E13.5 (but are absent at the PThE, an interesting differential characteristic with *Neurod1/2*). However, the amygdalar labelling is mainly present laterally (prospective BLP, BLA) and diminishes towards the caudomedial AHi (not shown). At E15.5, *Neurod6* transcripts have practically disappeared at the AHi mantle, but persist mainly in the mantle derivatives of the *basolateral* radial unit of the amygdala (not shown; see below at E18.5), as well as at the NLOT2ms, which appears labelled in a graded manner, with signal increasing rostralward (NLOTms; AHi; Fig. 12d). At E18.5 *Neurod6* expression clearly delineates at lateral sagittal section levels the whole *basolateral* amygdalar radial unit (the periventricular BLP and associated BLA and BLI intermediate masses, which approach the unlabeled superficial CxAC; Fig. 12e; Garcia-Calero et al. 2020). In more medial sections the BLA can be followed into its smaller medially deviated portion, which forms a cap on top of the BMA (BLAcap; BMA; Fig. 12f), and finally ends via the protruding BLA medial horn (see Garcia-Calero et al. 2020) into the labeled NLOT2ms (Fig. 12g). Since labelled BLA-related formations form at E18.5 a bridge with the remnants of the labeled NLOT2ms, whereas little labelling was found at the AHi with this marker, this pattern represents circumstantial evidence supporting a hypothetic posterolateral BLA-related lateral root of the NLOTms, which we postulated above tentatively. At E18.5, the NLOT2 adopts a mature shape and continues to express *Neurod6* (Fig. 12h). Interestingly, another gene, *Lmo3*, shows a very similar pattern as *Neurod6*, appearing expressed from E13.5 onwards mostly at the *basolateral* radial amygdalar unit (prospective L, BLA, BLI, BLP), as well as at the NLOT2ms, and finally at the NLOT2 (not shown; Allen Developing Mouse Brain Atlas).

Expression of *Lhx2* is widespread in the cortex, with reduced signal at the lateral and ventral pallium (Fig. 12i). At E13.5, ventricular zone expression at the hippocampal allocortex (but there is no signal at the cortical hem) is continuous with a small part of the pallial amygdala, identifiable topographically as the AHi primordium, under the caudal end of the lateral ventricle; there appears weaker signal at the anterior radial unit as well (Fig. 12i–k). A strongly labeled AHi ventricular zone and deep mantle, continuous caudally with the hippocampal cortical hem, was also present at E15.5 (Fig. 12l–n). The AHi *Lhx2* signal was still identified at E18.5 (not shown; Allen Developing Mouse Brain Atlas). The marginal migration stream of the PThEt also expresses *Lhx2* (not shown). At P4, the superficial derivative of this area, the PMCo nucleus, appears positive for *Lhx2*, jointly with the basolateral amygdalar derivatives (L, BLA, BLP) and the retroendopiriform nucleus (not shown; Allen Developing Mouse Brain Atlas).

*Emx1* appears expressed at E13.5 and E15.5 exactly at the same ventricular amygdalar place as *Lhx2* (Allen Developing Mouse Brain Atlas), which corresponds to a periventricular lamina of positive mantle cells at the *posterior* radial unit; there is also ventricular labeling of the hippocampal/entorhinal allocortex and the neocortex (AHi; Fig. 12o, p).

*Six3*-expressing neurons are present abundantly at the central amygdalar nucleus (CeA), as well as in radially related more superficial cells extending from CeA into the caudal part of the olfactory tuberculum (Allen Developing Mouse Brain Atlas). At E16.5, a section cutting horizontally the subpallial phase of the NTOL2ms shows dense *Six3*-positive cells contouring intimately laterally and medially the *Six3*-negative NTOL2ms (Fig. 9k). Similar cells also cover dorsally the NTOL2ms (not shown). A sagittal section at P2 shows the relationship of the *Six3*-positive CeA population with regard to the final NLOT2 formation (Fig. 9l).

### *Sim1* loss of function

As was reported for the hypothalamic Pa derivatives (Michaud et al. 1998), *Sim1* loss of function does not alter early differentiation patterns and migrations. *Sim1*-expressing mantle cells emerge in normal quantity, and migrate into characteristic positions. It is only after E15.5, when they should take the next step in differentiation towards more specific neuronal typologies (e.g., transforming under control of *Sim1* and *Brn2* into specific peptidergic phenotypes; see Michaud et al. 1998), that they fail to do so and start to die. Apparently, the earlier born superficial elements, such as the supraoptic nucleus, are more death-resistant than the later born deep ones (Michaud et al. 1998). Our *Sim1*-tauLacZ material partly shows the same pattern; up to E16.5, the embryos show apparently normal Pa/HyA and amygdalar *Sim1*-expressing mantle derivatives, but the *whole* amygdalar NLOT2ms apparently does not advance beyond the amygdalar pallium into the subpallial AA (Figs. 1n, 4a–g).

**Fig. 13:**
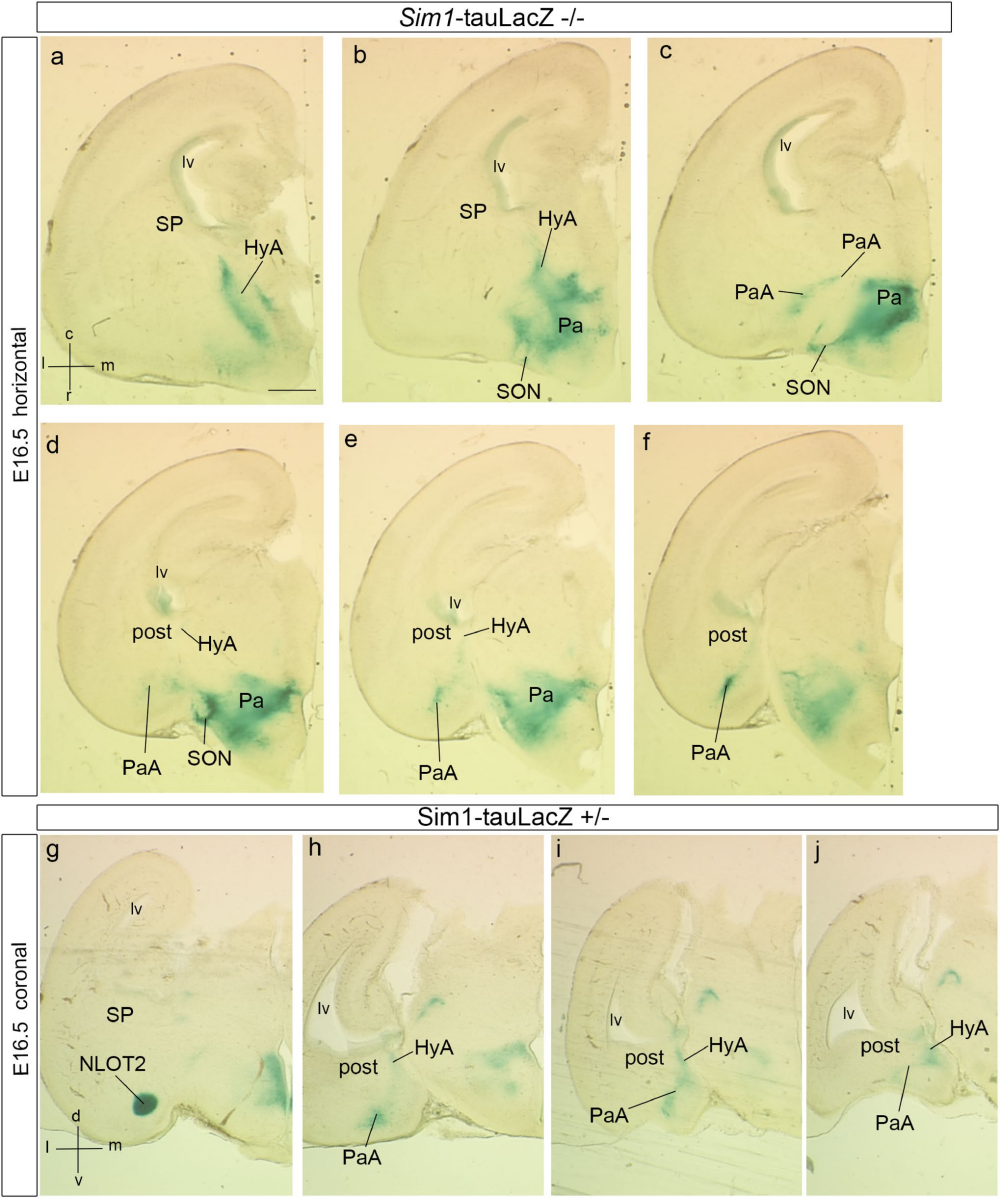
Sets of horizontal sections (dorsoventral order) and coronal sections (anteroposterior order) showing *Sim1*-tauLacZ reaction in the homozygote mutant (a-f) and the heterozygote (g-j) at E16.5. The spatial orientation appears at the bottom left-hand corner of **a**, **g**. **a**–**f** The homozygote mutant series starts dorsally with the HyA cell stream (possibly abnormally populated), cut along its terminal sulcus course (**a**), and then follows through various more ventral section levels in which a somewhat deformed (irregularly shaped) paraventricular area and supraoptic nucleus complex appear (Pa; SON; **b**–**f**). At some levels there are small remnant patches of blue cells at the posterior amygdala (post; **d**–**f**), and the rostral tip of the possible NLOT2ms remnant, practically disappeared, is observed at the level of the PaA cell group (PaA; **c**–**f**). **g**–**j** The coronal series through the heterozygote specimen shows essentially the wildtype pattern, starting rostrally with a typical rounded NLOT2 (**g**), followed by the PaA cell group and posterior amygdalar NLOT2ms remnant (PaA; post; **h**), and converging upon the rest of the HyA (HyA; **h**–**j**). For abbreviations, see list. Scale bar in represents 420 µm

**Fig. 14:**
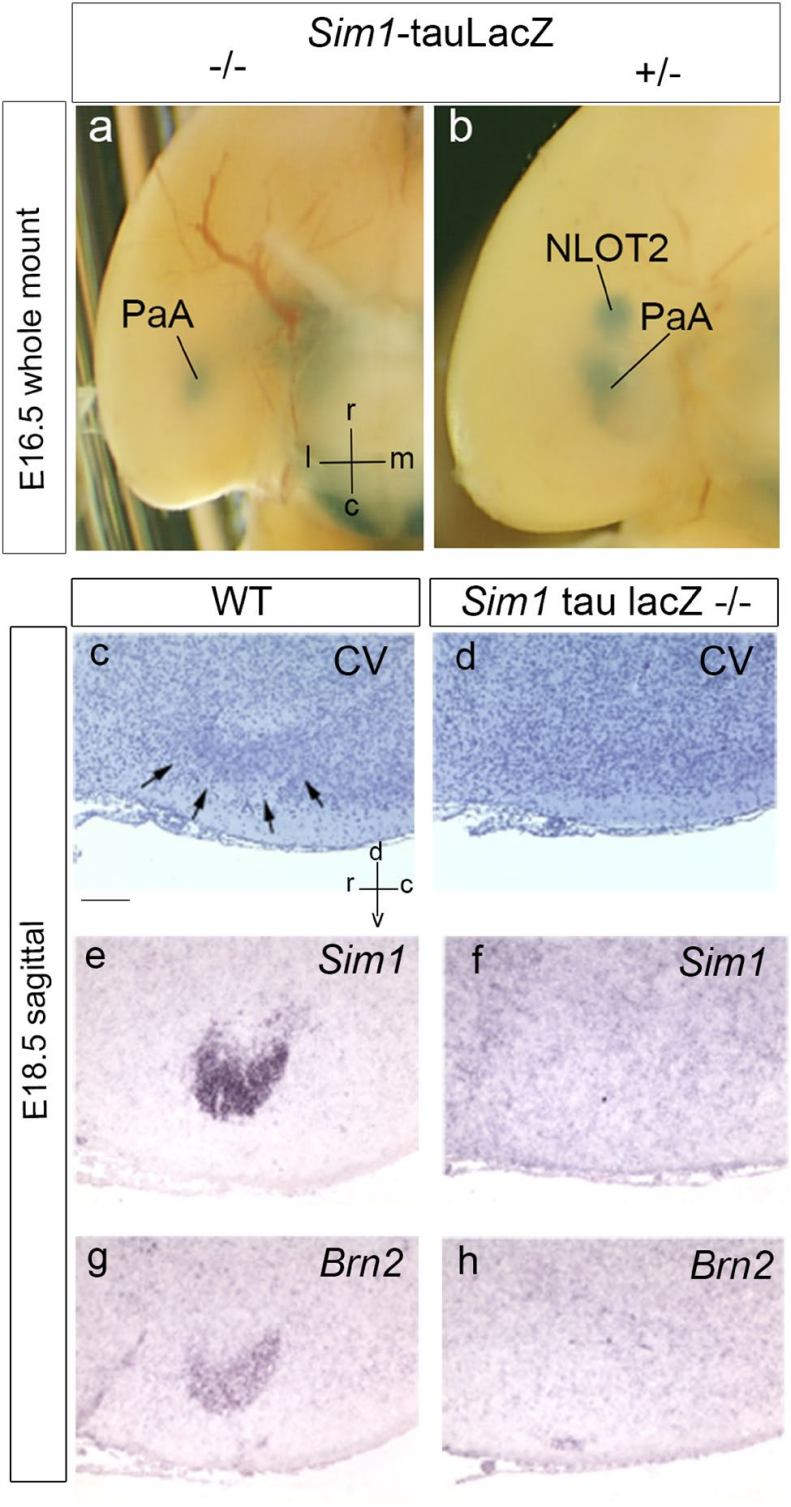
*Sim1* expression in wildtype and *Sim1* mutant mouse specimens at E16.5 and E18.5. The spatial orientation appears at the bottom right-hand corner of **a**, **c**. **a**, **b** Comparison of *Sim1*-tauLacZ whole-mount reaction in a homozygous *Sim1* mutant (**a**) and a heterozygous mutant at E16.5 (basal view; **b**). The homozygote shows only one positive patch, corresponding in position to the PaA cell group, whereas the heterozygote shows in addition the rounded NLOT2 patch. **c**–**h** Sagittal sections at E18.5 comparing with Nissl stain (cresyl violet; CV), and *Sim1*/*Brn2 *in situ the presence of the characteristic NTOL2 in the wildtype (WT), and its absence at comparable section level in the *Sim1* mutant homozygote. For abbreviations, see list. Scale bars represent, 1 mm (**a**, **b**), and 400 µm (**c**–**h**)

We studied E16.5 and E18.5 *Sim1* tau-LacZ homozygotes. Comparison of mutant and wild-type E16.5 cross-sections in Fig. 13a–j shows in the mutant a somewhat irregular HyA remnant, perhaps more populated than would be expected at this stage, likely reflecting a halted migration, with very limited AHi signal (Fig. 13a, b). There is also an irregularly shaped Pa mantle extending into the supraoptic nucleus locus (post; Pa; SON; Fig. 13b–f). The expected NLOT2ms appears reduced in volume and length, and looks as if a diminished NLOT2ms has diverted entirely into the position of the PaA cell population described above, associated to the medial edge of the BMA nucleus (Fig. 13c–f). This aberrant pattern contrasts to that of the *Sim1* tau-LacZ heterozygote, where both the NLOT2 and the PaA are distinct, as well as a normally dimensioned HyA remnant (NLOT2; PaA; post; HyA; Fig. 13g–j). Consistently, homozygote E16.5 wholemounts differ from heterozygous controls by showing at the brain base only one positively labeled patch (instead of the normal two) (Fig. 14a, b). We interpret that this single patch represents the PaA population described above within the amygdalar pallium, associated to the BMA nucleus, so that the subpallial NLOT2 patch is missing. These results suggest, first, that some *Sim1*-expressing mutant cells fated for the NLOT2 do not complete their migration into the posterior amygdala (AHi), remaining perhaps at the HyA (Fig. 13a, b). Those that do reach the amygdala and advance along the NLOT2ms either die after E15.5 (particularly those destined to the NLOT2), or fail to penetrate the AA (i.e., do not exit the pallial amygdala) and aggregate instead at the PaA. Coronal sections of E18.5 homozygote and wild-type specimens taken at NLOT level fully confirm this interpretation; the post-migratory wild-type NLOT2 is easily recognized in adjacent sections stained for Nissl, or reacted for *Sim1* and *Brn2* transcripts, whereas no sign of this structure was found in the E18.5 mutant (Fig. 14c–h).

## Discussion

The first goal of this study was to examine the *origin* of the *Sim1*-expressing young neurons that later populate the migrated NLOT2 nucleus. The second goal was to determine how the *Sim1* population *relates* to the stream of migrating pallial NTOL2 cells that occurs in the mouse roughly between E14.5 and E17.5 (Remedios et al. 2007). Our results unexpectedly illuminated the parallel issue of the ambiguous statements made by the cited authors on *pallial origin* and *migration course* of the NLOT layer2 cell population. We found that the Tbr1-positive neurons of the NLOT2ms (or CAS) originate from the *posterior amygdalar* radial unit (Garcia-Calero et al. 2020). Remedios et al. (2007) previously deduced that this component arises from a caudal extension of the dorsal pallium. Their origin and our origin seem topographically identical (present data), confirming an *amygdalar* and *non-cortical* source of the phenomenon.

Our recent analysis of the cortical and amygdalar pallium fields (Puelles et al. 2019a; Garcia-Calero et al. 2020; Garcia-Calero and Puelles 2020, 2021) leads us to doubt that it is possible to extrapolate cortical pallium sectors into the histogenetically separate amygdalar pallial field. This now obsolete analytic approach was actually initiated by our group (Puelles et al. 2000, and Medina et al. 2004), and its use is still common (e.g., Desfilis et al. 2018; Ruiz-Reig et al. 2018), but should be discontinued. Along the Discussion, we will argue that all the data considered by Remedios et al (2007) are coherently reinterpretable according to a simpler amygdalar origin hypothesis, without involving wider cortical relationships other than the hippocampal/entorhinal close neighbors. This option is advantageous at least in offering higher consistency with the known anatomy of developing and adult rodent brains, by recognizing that the primordium of the neocortex is always distant from the amygdalar field, due to their absolute separation by interposed mesocortical and allocortical pallial domains (Puelles et al. 2019a; Garcia-Cabezas et al. 2019; Pattabiraman et al. 2014; Bayer and Altman 1991; Swanson 1987).

**Fig. 15:**
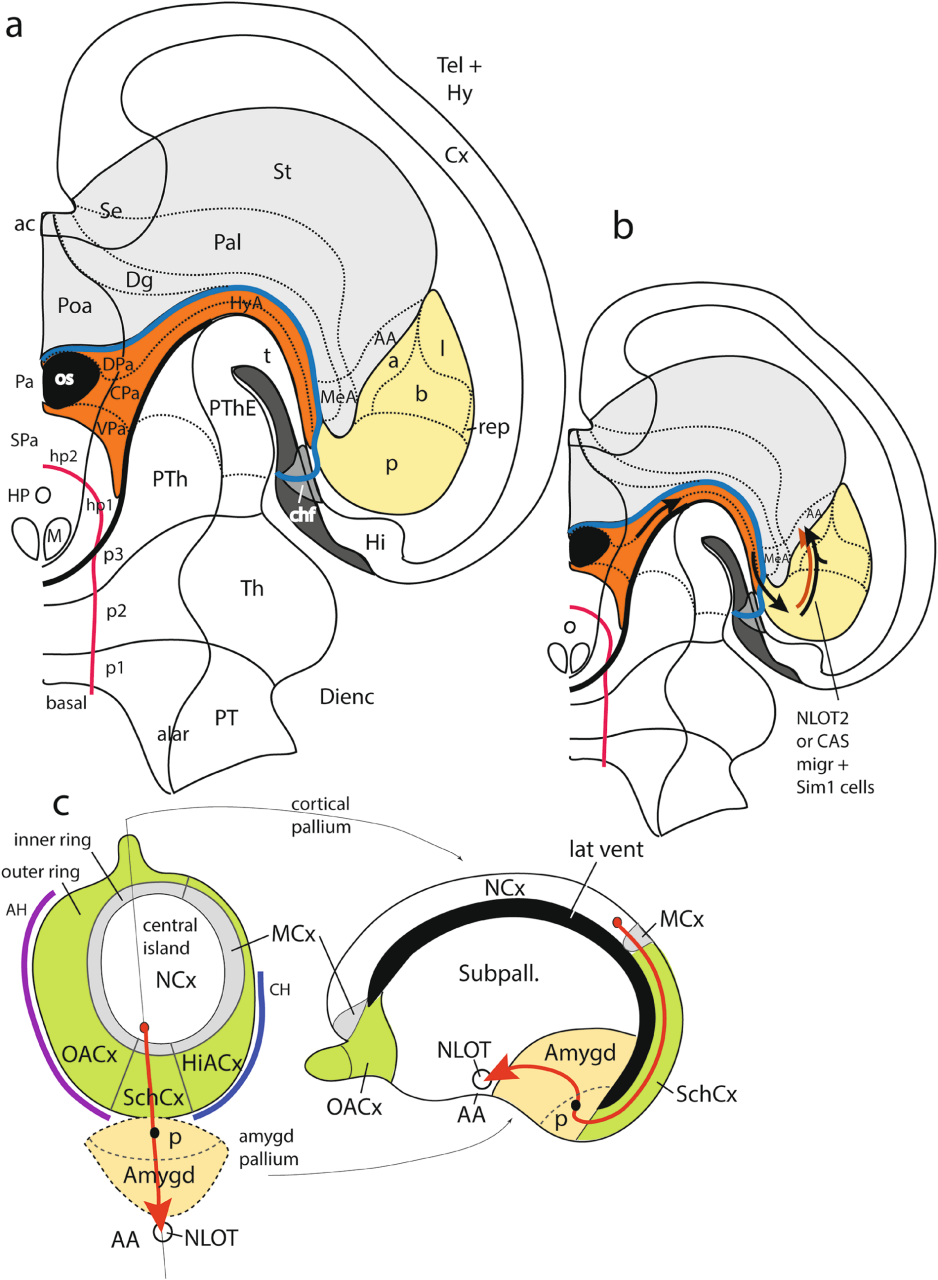
Schemata summarizing our findings according to the updated prosomeric model (Puelles and Rubenstein 2015). **a** This schema visualizes our present conception of the right half of the forebrain, after eliminating the upper alar plate and part of the roof plate of telencephalon and diencephalon by a horizontal section. The hypothalamo-telencephalic structural unit (secondary prosencephalon) appears separated from the diencephalon by a thick transverse black line. A thinner topologically transversal black line separates the hypothalamo-telencephalic neuromeres hp1 and hp2 units one from another. Similar lines separate the three diencephalic neuromeres (p3, p2, p1) and their major alar derivatives, prethalamus (with the prethalamic eminence bulging into the interventricular foramen), thalamus and pretectum (PTh/PThE, Th, PT; a part of the PThE is evaginated into the medial wall of the hemisphere (its ‘telencephalic’ part, marked here with a ‘t’). The medial hemispheric wall surface is identified in dark grey. At the midline there is caudally the basal plate (basal), limited by a red alar-basal boundary, and bending orthogonally into the rostromedian acroterminal domain (not marked, for clarity; it extends along the basal and alar midline from the mamillary area, M, where the forebrain floorplate ends rostrally, into the septal anterior commissure, ac). The basal plate encloses the mamillary bodies (M) and the tubero-infundibular region with the median hypophysis (HP). The hypothalamic alar plate, divided into its two prosomeres hp1 and hp2 appears partly in white (the subparaventricular area, SPa) and in orange (the paraventricular area, Pa, which encloses rostrally the optic stalk, os. The Pa is subdivided dorsoventrally into dorsal, central and ventral bands (DPa, CPa, VPa). Both DPa and CPa extend topologically dorsalward into the hypothalamo-amygdalar corridor or HyA (note this direction is deformed backwards by differential growth of the caudal telencephalic pole; the HyA tip is the dorsalmost hypothalamic alar derivative, because it touches the similarly deformed chorioidal *roof plate* present at the chorioidal fissure in the medial hemispheric wall; chf). The roughly longitudinal (but rather oblique) hypothalamo-telencephalic boundary is marked as a thick blue line separating the HyA from the subpallium (in pale grey) and the amygdalar pallium (in pale yellow). The major septo-amygdalar subpallial domains, preoptic area, diagonal area, pallidal area and striatum are identified (Poa, Dg, Pal; St). We simplified here the schema, because in fact the caudal subpallium would partly cover much of the pallial amygdala. We represent a state in which the torsion of the hemisphere is not completed; just imagine the central Pal and St extending backwards above the MeA and pallial amygdala, pushing the latter below the plane of the schema). All subpallial parts converge upon the septum (which is *not* a ventral, but a dorsal topologic entity, against what is repeated in the literature, since it encompasses the median commissural roof (e.g., the anterior commissure, ac, plus other telencephalic commissures more caudally, and adjacent extreme alar telencephalic subregions). The caudalmost subpallial regions are amygdalar, and include the anterior amygdala (AA) and the medial amygdala (MeA). The pallial amygdala (in pale yellow) appears divided into its five radial macrounits, anterior, lateral, basal, posterior and retropiriform (a, l, b, p, rep; based on Garcia-Calero et al. 2020). Note the HyA corridor leads into the rostromedial part of the posterior unit (p), just behind the end of the subpallial MeA. The schema identifies as well-general cortex (Cx) and hippocampal allocortex (Hi; note this receives the telencephalic insertion of the roof chorioidal fissure (chf) at its alar border, the cortical hem (not identified). **b** This second schema is essentially the same as in **a**, without most letterings, and is used to make clear the course followed by *Sim1*-expressing cells (arrows). These originate within the hypothalamic CPa and the HyA (see “Discussion”). They reach the *posterior* amygdalar pallium unit (p) behind the MeA, and incorporate (orange arrow) into the caudorostral NLOT2 or CAS pallial Tbr1-expressing migration stream (black arrow, with main posterior, p, origin and possible secondary, smaller origin at the basal unit, b; see schema **a**). The mixed arc-shaped migration stream courses first through amygdalar pallium, but finally crosses the limit of the subpallium and ends superficially at the anterior amygdala (AA). The conclusion is that the primary origin of the Tbr1-positive CAS coincides with the locus where the hypothalamic *Sim1* cells arrive at the posterior pallial amygdala, and both populations compose the CAS or NLOT2 stream. **c** This figure shows a flattened topological schema distinguishing the amygdalar pallium (pale yellow) from the cortical pallium, which is divided into a central neocortex island (NCx, white) surrounded by a thin mesocortical inner ring (MCx; light grey) and a partly broader allocortical outer ring with olfactory, entorhinal/schizocortical and hippocampal subregions (OACx, SchCx, HiACx; green). The violet colored line alongside the OACx symbolizes the antihem (AH), whereas the blue colored line alongside the HiACx represents the cortical hem (CH). This cortical map reproduces notions reported in Puelles et al. (2019a), partly inherited from previous literature. As shown in **a**, normally the cortex covers topographically the amygdalar pallium, causing the erroneous impression that the pallial amygdala is a cortical derivative. In **c**, we represent its true topological subjacent position, obtained if the respective ventricular zones (seen with the same color-code in **d**) are separated and flattened out. The posterior pallial amygdala (p) falls close to the SchCx and the caudal ends of the OACx and HiACx (and of the AH, CH). The position of the final NLOT nucleus within subpallium (AA) appears in **c** at the bottom of the schema. According to us (compare **b**), the mixed CAS migration starts from the black dot within (p), and then proceeds along the red arrow into the AA. According to Remedios et al. (2007), the CAS migration would start at the NCx (red dot) and follow a longer route to reach the AA (but they also wrongly think that the NCx extends initially into the posterior amygdala; however, note the intercalated mesocortical and allocortical expanses of cortex). **d** This schema is complementary to **c**, and represents a sagittal section through it passing from the rostral olfactory bulb across the whole cortex, and in particular the SchCx, into the pallial amygdala (thin line in **c**). The cortical pallium is represented with the same color code as in **c** (NCx, white; MCx, light grey; OACx/SchCx, green). In this schema, the structure is unflattened, and shows the standard sagittal section configuration (lateral ventricle cavity in black). Note the rostral relationship of the pallial amygdala (yellow) with the subpallium that partly covers it, as mentioned above (Subpall.; white; compare **a**, **b**) and its caudal relation with allocortical cortex domains, closest to the posterior amygdalar radial unit (p) where the CAS migration originates (p; black dot; red arrow into NLOT/AA). As in **c**, we also represent with an alternative red dot origin of the red arrow the longer course predicted from an hypothetic NCx origin of the CAS (unless it is proven that the NCx ends caudally at the posterior pallial amygdala, disrupting the continuity of the meso- and allocortical rings)

With regard to *Sim1* cells, we described a *Sim1-*expressing *hypothalamo-amygdalar corridor* (HyA). Fan et al. (1996) first mapped it at E10.5 and E12.5, but left it unnamed (see their Figs. 1n, 15a). In agreement with tentative schemata of Puelles and Rubenstein (2003, 2015) on this point, we think that the HyA is a dorsal prolongation of the hypothalamic paraventricular area (Pa) that results co-evaginated into the early telencephalic vesicle jointly with the neighboring rostrodorsal ‘telencephalic’ part of the diencephalic prethalamic eminence (PThEt; Puelles 2019). This implies *a novel concept* which may be of interest in comparative neuroanatomy, namely the existence of an alar hypothalamic subdomain that stretches into the telencephalic roof without losing its original molecular character (Fig. 15a). This hypothesis explains the course of HyA through the floor of the interventricular foramen and of the terminal sulcus (Fig. 15a). The HyA always lies next to neighboring subpallial formations (e.g., main BST nuclei, supracapsular BST and MeA) and finally reaches the *posterior pallial* amygdala at the end of the chorioidal fissure (Fig. 15a).

The Pa/HyA progenitor domain represents the apparent origin of all forebrain *alar Sim1*-expressing cells (there are separate basal ones; Fig. 1n). This implies that the derivatives of this area must include the population that reaches the amygdala and eventually enters the NLOT2. The HyA can be understood as the migratory pathway for the arrival of paraventricular *Sim1* and *Otp* cells to pallial or subpallial parts of the amygdala, as was already demonstrated for Otp cells (Wang and Lufkin 2000; Garcia-Moreno et al. 2010; Morales-Delgado et al. 2011; Morales et al. 2021; present results; see also Bardet et al. 2008 for a comparative perspective). Considering that various names applied previously to this pathway were conceptually inappropriate (see below), we renamed it ‘hypothalamo-amygdalar corridor’ (HyA), emphasizing its hypothalamic origin and molecular profile, as well as its amygdalar ending.

The topographically caudal end of the HyA corridor (which topologically is actually its *dorsal end*, where the hypothalamus reaches the chorioidal roof plate; Fan et al. 1996) allows the access of *Sim1* cells to the extreme caudomedial part of the pallial amygdala (namely its *posterior* radial unit, or prospective AHi/PMCo complex). The invasion occurs specifically at its *rostromedial* subdivision (AHiRM; Garcia-Calero et al. 2020), which characteristically protrudes into the medial brain surface with the underlying PMCo nucleus, *behind* the subpallial MeA (p; MeA; Fig. 15a; De Olmos 2004).

Analysis of the *posterior* amygdalar radial unit in horizontal sections at E13.5 and E14.5 revealed that the migrated *Sim1*-expressing cells first shift laterally within AHi, passing behind the MeA, and adopting a new position within the rostrolateral AHi subdivision found *lateral* to the MeA (Fig. 15b). Here they join the Tbr1-positive local elements that start to migrate into the arc-shaped NLOT2 migration stream. The latter, aptly (though somewhat ambiguously) named the ‘caudal amygdaloid stream’ (CAS), arrives at its target between E16.5 and E17.5 (Remedios et al. 2007). Our NLOT2ms results corroborate absolutely these earlier data, adding some points of interest.

We examined in more detail the course of the arc-shaped *Sim1*-expressing NLOT2ms/CAS using Tbr1 immunoreaction and *Dlx5* ISH, which respectively mark the pallium versus subpallium domains. We thus were able to divide the migration into successive *pallial* and *subpallial* phases. The first phase traverses several parts of the pallial amygdala, always next to the pallio-subpallial boundary, proceeding orthogonally to the local radial glia between E14.5 and E15.5 (as already noted by Remedios et al. 2007, and corroborated by us; Fig. 15b). Once the stream reaches at about E15.5 the medial side of the BLA and BMA nuclei (*basolateral* and *anterior* radial amygdalar units; b, a; in Fig. 15b; Garcia-Calero et al. 2020), the NLOT2ms/CAS proceeds into its second *subpallial* phase. To this end it crosses the pallio-subpallial border into the subpallial anterior amygdala (AA), and advances therein in a radial course into its target locus (Fig. 15b, d), surrounded by dispersed *Dlx5-*, *Six3-*, *Pax6-*, calbindin, and *Lhx9/Lhx2*- expressing cells. Here the migration ends, and the NLOT nucleus forms. Remedios et al. (2007) observed these two phases as regards the changing relationship with radial glial processes (tangential to radial), but they apparently did not notice that the change also coincides with the pallial versus subpallial character of the tissue surrounding the migrating cells. We think this result has relevance towards understanding the roles of diverse genes known to control this migration (see below). The decision point where the cells change into the second phase lies next to the *anterior* radial unit of the pallial amygdala (prospective BMA/ACo nuclei), and in the medial vicinity of the BLA nucleus (intermediate mass of the *basolateral* unit; Garcia-Calero et al. 2020).

The convergence of the hypothalamic *Sim1*-expressing HyA pathway with the *posterior* amygdalar source of migrating Tbr1-positive pallial cells (Fig. 15b) clearly identifies the origin of the NLOT2ms/CAS migratory process as the *posterior* unit of the pallial amygdala. The latter contacts caudally the *hippocampus* and *entorhinal cortex*, both of them allocortical (De Olmos 2004; Franklin and Paxinos 2013; Puelles et al. 2019a; Garcia-Calero et al. 2020). In contrast, Remedios et al. (2007), referring to our amygdalar AHi/PMCo complex, reported that the migration originates from a caudal part of the dorsal pallium, thus pretending to establish a ‘link between the amygdala and neocortex’. This conclusion is clearly contradictory with the easily observable caudal direct boundary of the AHi *posterior* unit of the pallial amygdala with the hippocampus (as appears reflected in its conventional name, ‘amygdalo-hippocampal transition area’). Several other commentaries, reviews or reports accepted the Remedios et al. (2007) interpretation of the CAS migration as a cortical dorsal pallium phenomenon without raising doubts or objections (Deussing and Wurst 2007; Subramanian et al. 2009; Murillo et al. 2015; Ruiz-Reig et al. 2017; Chou and Tole 2019). In a recent review of cortical models featuring classically defined concentric ring-shaped domains (Puelles et al. 2019a) we did express doubts about this hypothesis of Remedios et al. (2007), since all these cortex models showed the neocortex to be *totally separated* from the amygdala by the massive outer allocortical ring and the thinner mesocortical ring.

We studied carefully the Remedios et al. (2007) paper, exploring various ways to understand what seemed a remarkable error of interpretation coming from first-rate researchers. We reached the conclusion that the paper includes various sorts of doubtful assumptions and interpretive errors that were actually widely shared (including by us) in the field of pallial studies back in the first years of 2000. We will divide our resulting interpretation into three levels of analysis.

First, we will examine whether there is discrepancy between Remedios et al. (2007) and present results about the *observed embryonic location* and *adult identity* of the origin of the NTOL2ms/CAS phenomenon. The possible use of ambiguous terms might have approximated at least implicitly our respective interpretations. It seems clear that we both see the origin of the NTOL2ms at exactly the same place of the embryonic telencephalon, but for various reasons we classify this locus as *posterior pallial amygdala*, whereas Remedios et al. (2007) classified it as *dorsal pallium*. We cannot be both right.

In a second, more semantic level of analysis, we consider whether there existed in 2007, and still exist today, solid grounds to classify the particular telencephalic locus where the CAS originates as ‘dorsal pallium’. This question possibly needs a ‘yes and no’ answer, because such a notion was indeed possible, and even conventional, during a number of years, but this has changed now, turning it into a risky idea. We also must address the scientific meaning of ascribing an embryonic brain pallial locus to a given *pallial cortical sector*, such as the ‘dorsal pallium’. The molecularly distinct pallial sectors with which Remedios et al. (2007) dealt were partly corroborated *postulates* within a conceptual pallium model that is now 20 years old (Puelles et al. 2000; Medina et al. 2004; Tole et al. 2005, and has recently evolved for good reasons to different postulates. The relevant assumptions have indeed changed significantly in recent years (Puelles 2014, 2017; Puelles et al. 2016b, c, 2017, 2019a).

Finally, we will discuss the notion of Remedios et al. (2007) that the dorsal pallium, the primordium of neocortex, actually extends caudalwards between the caudal ends of hem and antihem to establish as its caudalmost subregion the locus of CAS origin. This idea seems inconsistent with available developmental and adult rodent brain neuroanatomic knowledge. Tradition in this field does not detect any direct contiguity whatsoever between neocortex and pallial amygdala (Puelles et al. 2019a; Garcia-Cabezas et al. 2019; Pattabiraman et al. 2014; Bayer and Altman 1991; Swanson 1987).

Proceeding now to our first topic, we inquire whether we really disagree about the embryonic location of the source of NLOT2ms or CAS. As stated above, interpretation of our results within our recent radial amygdala model (Garcia-Calero et al. 2020) allows little doubt that this origin lies in the *posterior* pallial amygdalar unit (AHi/PMCo complex). On the other hand, Remedios et al. (2007) vaguely described this locus as the ‘caudal telencephalic neuroepithelium’ or ‘the caudal extreme of the telencephalon’ (for instance, in the legend to their Fig. 2, while locating their electroporation experiment). They probably referred to the local end of the lateral ventricle. According to us, the neuroepithelium at the front of the end of the ventricle is in large part pallial amygdalar (note subpallial amygdala also steps in somewhere), whereas the neuroepithelium that lines caudally the end of the ventricle is hippocampal and entorhinal (check the plates in Garcia-Calero et al. 2020). Remedios et al. (2007) seem to have lacked these anatomic references. The description of their important electroporation experiment did not include any suggestion that the experimental site lies *rostral to the lateral ventricle*, as is clearly visible (see their Fig. 2). This anatomic locus is systematically ascribed to the *amygdala* in all developing rodent brain atlases (e.g., Altman and Bayer 1995; Alvarez-Bolado and Swanson 1996; Jacobowitz and Abbott 1997; Foster 1998; Paxinos et al. 2007; Ashwell and Paxinos 2008).

After proceeding to an analysis of ventral, lateral, medial and dorsal pallium markers which was concluded to exclude the first three options, Remedios et al. (2007) jumped to the conclusion that the caudal locus of CAS origin had to be an integral part of the remaining option, the dorsal pallium, adducing some molecular evidence (considered below) and the existence of a gap between the hem and antihem organizer territories at the periphery of the pallium, which might allow the dorsal pallium to extend into the area of CAS origin. The ambiguous term ‘caudal amygdaloid stream’ (CAS) they used does not refer to an *amygdalar origin* of the NLOT2, but to a caudal *dorsopallial* (neocortical) origin of this migrated, finally ‘amygdalar’ structure. The authors apparently did not conceive any primary amygdalar non-cortical pallial neuroepithelial region at the ‘caudal telencephalic neuroepithelium’, though their own radial glia preparations clearly suggested it (their Figs. 7a–c, 8h). They apparently believed, probably inspired by our earlier study of ventral and lateral pallium parts of the amygdala (Medina et al. 2004), or by other contemporaneous sources, that *all parts* of the pallial amygdala originate in one of the four postulated cortical pallial sectors, and secondarily migrate to distinct final amygdalar sites. To illustrate this notion, Remedios et al. (2007) drew dashed lateral and ventral pallium *arrows* in their Figs. 1 and 8, complemented by their dorsopallial CAS, jointly depicting the hypothetic paths of amygdalopetal migrating cells originated in various parts of the cortex. Similar arrows were reproduced by Deussing and Wurst (2007; their Fig. 1), reflecting editorial approval of this notion. The whole Discussion of Remedios et al. (2007) developed thereafter out of the initial ‘dorsal pallium’ diagnosis. We did not find any place where Remedios et al. (2007) considered even hypothetically that the CAS locus of origin might be intrinsically pallial amygdalar (this option might have been qualified to represent an *analog* of cortical dorsal pallium in terms of its molecular profile). We understand that Murillo et al. (2015) and Ruiz-Reig et al. (2017) later simply accepted the scientific authority of the respected research group and Nature Neuroscience.

We initially also thought in much the same way, since we also shared the assumption of varied cortical origins of distinct amygdalar components, all the way from Puelles et al. (2000) to Puelles et al. (2016b). Subsequently, we started to doubt such interpretations, as we gradually assimilated various contradictory data accruing from the work of Gorski et al. (2002), Puelles (2014) and Puelles et al. (2016c), as we will comment below. We have since realized that this conventional viewpoint on the mode of pallial amygdala formation is unfortunately erroneous. This happens, partly, but importantly, because all studies performed in this period confided on coronal sections *oblique* to radial amygdalar structure. In such sections, we never see the true amygdalar ventricular zone, because the latter appears in other coronal sections *caudal* to the amygdala, precisely at the ‘caudal telencephalic neuroepithelium’ of Remedios et al. (2007). That is the simplest reason why these authors missed the true amygdalar identity of the CAS origin.

We thus conclude that Remedios et al. (2007) did *not* discover or deduce that the CAS/NLOT2 origin is at the *posterior* part of the pallial amygdala because they ignored (as did everybody else at the time) that the latter, equivalent to their ‘caudal neuroepithelium’, is a *local pallial amygdalar progenitor domain,* that does not derive from the cortical dorsal pallium, or from any other cortical subregion. The cause of this error was the widely shared assumption that *cortical* pallial sectors produce claustral and amygdalar nuclear populations that migrate long distances into extracortical adult positions, such as the amygdala. This false assumption, tied methodologically to the use of coronal sections, misled Remedios et al. (2007) into the simplistic and, as it turns out, false conclusion involving the actually distant dorsal pallium as the origin of the pallial cells of the CAS/NLOT2.

The implications of such biased thinking, given the real distance of the amygdalar target from the molecularly delimited dorsal pallium (Puelles et al. 2019a), are reduced to the absurd in our Fig. 15c, d (red dots and lines). The data produced by Remedios et al. (2007), and notably their excellent electroporation experiment (whose position we represent as a black dot within the posterior amygdala; Fig. 15c, d), do not support long-distance migration of the CAS. Such translocation would be needed if these cells really came from the dorsal pallium (red dot). The site labeled by electroporation clearly corresponds to a posterior amygdalar pallial site, not to any distant dorsal pallium cortical sector. We will come back to this conclusion under the third point of discussion.

As a second topic of clarification, we examine now how solid were the grounds used by Remedios et al. (2007) to classify as ‘dorsal pallium’ the caudal telencephalic locus where the CAS/NLOT2ms originates, and whether their argument remains valid today. We think that they reasonably excluded the ventral and lateral pallial sectors, but not so the medial pallium, as we will see below. We also address the *meaning* and *interest* of ascribing an embryonic pallial locus to a given *pallial sector* postulated within a model (Puelles et al. 2000).

In the molecular era of pallium developmental studies, started roughly between 1998 and 2000, ascriptions of pallial elements to the postulated four sectors of the Puelles et al. (2000) pallium model proceeded by demonstrating a particular molecular marker profile shared by the corresponding territory. This was the case even when the pallial portion of interest was *not cortical* in structure (there are pallial parts of the septum and of the amygdala which essentially lack cortical structure; see also more general comments on comparative studies of non-corticoid pallium regions of non-mammals in Puelles 2017 and Puelles et al. 2017).

Remedios et al. (2007) employed the standard approach in their Results and Discussion, but did not consider first whether their area of interest—the ‘caudal telencephalic neuroepithelium’—was cortical or nuclear (i.e., amygdalar) in nature. This possibly distracted their attention from the theoretical possibility of a primary amygdalar classification of the CAS origin. Eventually, after thinking they had excluded the molecular profiles of the ventral, lateral and medial cortical sectors, they concluded that the data available pointed clearly enough to the single remaining option, the ‘dorsal pallium’. We think in retrospect that the markers they considered specific of the dorsal pallium, mainly *Emx1* and *Lhx2* (as well as *Neurod1/*2 and *Tbr1*) were not really ‘dorsal pallium’ specific. These signals also extend importantly at appropriate early stages within the mediopallial primordia of hippocampal and entorhinal cortical areas, even though the hippocampal *hem* is indeed negative for *Lhx2*, as was pointed out by these authors (see our Figs. 9d, e, l, 10, 11a–j, 12). These mediopallial or allocortical areas are close neighbours of the amygdala (see Hi, ERh, MP; Fig. 12a, b, d, j–p), much closer than the dorsal pallium or neocortex (DP). This close relationship was also evident in some of the illustrations of Remedios et al. (2007), such as their Figs. 1b, c, 2a, 3b, i–m, 4t, 8a, as well as in various other reports of the same group illustrating *Lhx2* expression. The lapse in recognizing a lack of specificity of these markers is difficult to understand. Probably other less relevant comparative considerations, like the sharing of a reelin/Dab1/Cdk5 neuronal migration control mechanism at both the CAS and the dorsal pallium, or their new data on a gap between the hem and antihem organizers, added salience to the ‘dorsal pallium’ hypothesis. The interesting comparison made in their Fig. 4w between *Wnt2b* (a hem marker) and *sFrp2* (an antihem marker) against the pallial *Emx1* signal should have been accompanied by a similarly oriented image comparing the same landmarks with *Lhx2*. Note the *Lhx2* pattern shown in their Fig. 4t is quite different from that of *sFrp2* in Fig. 4r. To identify the hem versus antihem is not the same as identifying the medial pallium versus the dorsal pallium, because these specializations lie topologically* outside* the medial and ventral pallium allocortical regions, and do not contact the dorsal pallium at all (Fig. 15c). We thus believe Remedios et al. (2007) did not attend to this diagnostic task optimally, according to available evidence. In our opinion, the authors should have concluded that the area of interest could as likely be either medial pallium or dorsal pallium. Between these two possibilities, the most parsimonious option was to ascribe the caudal area of interest to cortical *medial pallium*, rather than to dorsal pallium, due to the observable vicinity of mediopallial cortical areas to the area of interest. More indirectly, the then known joint requirement of hem-related *Emx1/Emx2* function for NLOT development also bespoke of a *mediopallial* relationship (Tole et al. 2005; Shinozaki et al. 2004; Suda et al. 2010). This viewpoint was indeed reached 7 years later by Abellán et al. (2014), who emphasized a number of shared LIM-homeobox genes between the medial pallium and the AHi/PMCo amygdalar complex, that is, the amygdalar source of the CAS.

We next consider critically the rationale of ascribing any amygdalar pallial parts to one of the four pallial* cortical* sectors defined by Puelles et al. (2000). We presently hold that, in any case, the seemingly reasonable pallial structure assumptions that Remedios et al. (2007) employed back in 2007 are now outdated (followed also, implicitly, by Deussing and Wurst 2007; Subramanian et al. 2009; Murillo et al. 2015; Puelles et al. 2016c; Ruiz-Reig et al. 2017; Chou and Tole 2019).

Historically, Puelles et al. (2000) and Medina et al. (2004) used a peculiarity in *Emx1* expression (its absence in a previously non distinguished portion of cortical pallial mantle lying next to the subpallium) to develop a *tetrapartite* mouse/chick pallium model that left behind the classic *tripartite* pallium model (the latter only contemplated lateral, dorsal and medial constituents; see Striedter 1997). Four (ventral, lateral, dorsal and medial) pallium sectors were distinguished at middle levels of the hemisphere. Though it was based on rather sketchy molecular data, this model apparently was valid ab initio for tetrapods (Smith-Fernández et al. 1998; Puelles et al. 2000). Corroborations of the new tetrapartite model (demonstrating presence of the *Emx1*-negative VPall) accrued thereafter in various anamniote species, as well as in man, giving ample credibility to the model. It was initially unclear whether the four pallial sectors extend throughout the whole length of the telencephalic pallium, that is, e.g., whether they reach the caudal amygdalar pole in a parallel arrangement of longitudinal partitions, as had been theorized previously by Kuhlenbeck (1973), Holmgren (1925), and other classic authors. Recently we have learned that the four cortical pallial sectors *do not reach the pallial amygdala*, due to the emerging alternative *concentric ring-shaped arrangement* of allocortical and mesocortical areas around a central iso/neocortical island (Garcia-Cabezas et al. 2019; Puelles et al. 2019a; see other references therein, an idea ranging back at least to Swanson 1987). Medina et al. (2004) first addressed the partial demonstration of parallel longitudinal pallial cortical sectors expected to reach the amygdala, using chosen markers thought to define the novel ventral and lateral cortical pallium sectors. The conclusion was that, according to the distribution of such markers, both ventropallial and lateropallial cortical sectors seemed to extend caudalwards into the pallial amygdala, each having specific *radially migrated* amygdalar nuclear derivatives. This tendentious, rather preconceived conclusion inspired in what one sees in coronal sections lamentably soon became a sort of established fact, or strong assumption, in the field, leading to the non-fundamented stream arrows drawn by Remedios et al. (2007) and Deussing and Wurst (2007).

Indeed, the illustrations of the Medina et al. (2004) report, based on standard coronal sections and artist drawings, *arbitrarily* suggested that the sources of any claustral or amygdalar nuclear structures lay at the ventropallial or lateropallial *cortical* ventricular zones *appearing in the same sections*, next to the pallio-subpallial boundary. This was an error inconsistent with the glial structure already known at the time, because no radial glia processes extend from the cortical ventral pallium into amygdalar pallial territory (the ventropallial glial processes rather plunge straightforwardly into the local olfactory cortex). Nobody recognized this conceptual error at that moment, due to the conventional massive use of coronal sections and the mentioned assumptions. These induced subliminally the belief that the nuclei you see in one coronal section come from the ventricular zone you have in that same section (disregarding other possible origins more rostrally or caudally in the brain).

It so happens that telencephalic coronal sections are *oblique* by some 45 degrees to *amygdalar* radial glial structure, because the ventricular zone where amygdalar nuclei actually arise lies *behind* the amygdalar nuclei, precisely at the ‘caudal telencephalic neuroepithelium’ whose glial structure was examined by Remedios et al. (2007), whereas the corresponding pial surface lies *ventrally* to the amygdala (Garcia-Calero et al. 2020; Garcia-Calero and Puelles 2020). Many authors accepted at face value the fore mentioned arbitrary conclusion about cortical origins of amygdalar cell masses held to migrate via ‘ventropallial and lateropallial migration streams’ into secondary amygdalar positions. The list of reports incurring in this inherited error includes Remedios et al. (2004, 2007), Tole et al. (2005), Subramanian et al. (2009), Martínez-Garcia et al. (2007, 2012) and Olucha-Bordonau et al. (2015), and there surely are other cases, including Puelles et al. (2016c).

A *lateropallial* migration stream that ends superficially in a dorsal part of the olfactory cortex, as proposed by Puelles et al. (2000) and Medina et al. (2004), was never demonstrated in terms of radial glia or experimental analysis. A later re-examination of this conundrum led LP to redefine the concept of lateral pallium as a radially organized ‘claustro-insular’ complex, now believed to represent the true lateral pallium, whereas the whole olfactory cortex resulted ascribed to the updated ventral pallium (Puelles 2014). Remarkably, the new lateropallial cortico-nuclear unit also appears with corresponding topology and selective gene markers in chick and reptiles, where nobody had previously expected to find claustrum and insula homologs (Puelles et al. 2016b, 2017, Puelles 2017; these surprising results were recently corroborated with a transcriptomic approach in reptiles by Tosches et al. 2018 and Norimoto et al. 2020). However, as concluded in the cited reports, as well as in Puelles et al. (2019a), this updated lateral pallium has *no molecularly identifiable derivative in the mouse amygdala*.

Contrarily, the postulated *ventropallial* migration stream is easily observable with radial glia stains (Puelles 2014; Garcia-Calero et al. 2020), but throughout its whole length it leads superficially into the olfactory cortex, *not into the amygdala* (see experiments labeling radial glia in Garcia-Calero et al. 2020). Another conflict with the supposed amygdalar ventropallial derivatives had appeared previously in the work of Gorski et al. (2002), who found that *all parts* of the pallial amygdala contained *Emx1*-derived progeny (labeled with *Emx1*-LacZ). According to the pallial model in vogue this marker supposedly had to be *absent* in true ventral pallium derivatives,* held to include most amygdalar nuclei*. Medina et al. (2017) and Desfilis et al. (2018) reproduced the Gorski et al. (2002) result in a lizard, and proposed a possible distinct ‘ventrocaudal pallial sector’, restricted to the amygdalar domain, as the origen of amygdalar *Emx1* cells (such solution was already mentioned as a possibility in mouse in Puelles et al. 2016c; however, this hypothesis has not yet been correlated with the recent radial model of the mouse amygdala which we presently use; Garcia-Calero et al. 2020). At the time of the Gorski et al. (2002) paper, we did not understand what this sharp contradiction might mean, but we were motivated to accommodate these and other discrepant data in a better model. Unfortunately, we also remained dominated by coronal section-driven assumptions for years.

Our last coronal section-based effort to define the ventropallial contribution to the amygdala used analysis of the theoretically conclusive *Dbx1*-derived progeny, since *Dbx1* expression was held to be strictly restricted to cortical ventral pallium progenitors (Medina et al. 2004; Puelles et al. 2016c). What we did not consider significant was that *Dbx1* also appears expressed primarily, and, actually, more importantly, at the ‘caudal telencephalic neuroepithelium’ (Bielle et al. 2005; Teissier et al. 2010), probably because we thought we already knew where the amygdalar nuclei came from. Our standard coronal analysis surprisingly produced various results that seemed contradictory, inconclusive, or difficult to explain. This included parts of olfactory cortex (and the whole olfactory bulb) which lacked *Dbx1* derivatives largely, or altogether, or only *halves* of some amygdalar nuclei appearing to be positive. It was also unclear that the observed *Dbx1*-LacZ labeled amygdalar elements had actually migrated through the distinctly labelled ventropallial migration stream seen reaching olfactory cortex *just outside* of the pallio-subpallial boundary and of the pallial amygdala. Importantly, the amygdalar *periventricular* masses at the back of the amygdala oddly belonged likewise to *Dbx1* progeny (partly seen, for instance, in Figs. 3c–e and 4a–e in Puelles et al. 2016c). These data were unexplainable by the standard model we were using. This led to offering two alternative interpretations (so far both unverified, and maybe both wrong), proposed respectively by Medina and Puelles, since we could not convince each other about either interpretation.

Eventually, we realized that the source of all these incoherencies of the tetrapartite pallial model as regards derivatives in the pallial amygdala were two false assumptions. (1) The assumption that coronal sections of the hemisphere identify the real neuroepithelial origin of amygdalar pallial formations, and, (2) the assumption that a ‘molecularly similar’ *amygdalar nucleus* should derive from the comparable *cortical sector*, without showing anything of its primary cortical nature. It was simply *wrong* and *misdirected* to expect cortical pallial sectors to produce parts of the nuclear amygdalar domain. The latter clearly represents a *different* sort of pallium adjacent to the cortex, which has *its own progenitors*, as happens likewise at the pallial septum. The issue of* shared gene markers* was just a distracting circumstance, whose significance seemed more important when we only were able to study a few markers. Nowadays we can inspect thousands of gene markers in databases, and know that distinct cortical or nuclear fates result from complex combinations of hundreds of gene functions. The existence of a few shared markers is not important, though some interesting correlations may be drawn out of them (Nieuwenhuys and Puelles 2016). A case in point is the fact that olfactory bulb efferents innervate both the olfactory cortex and several superficial amygdalar nuclei (although these do not derive from the former’s progenitor domain). This might be due to effects of shared genes guiding these fibres. Our point is that there is no predictive value of interest in explanations of amygdalar nuclear *structure* that simply are based on a presumed cortical origin.

This novel analytic light put us in the search of credible origins of the amygdalar nuclei, and we were helped by the idea to use as guides the radial glia processes (first investigated in the area of interest by Remedios et al. 2007, and later also by Bupesh et al. 2011). Many hours of work afterwards, and having checked carefully over 80 promising amygdalar gene markers, and similar numbers of cortical markers, we produced first our updated version of the previously existent *concentric ring model* of cortical pallium (see references reviewed in Puelles et al. 2019a, as well as Barbas 2015; Garcia-Cabezas et al. 2019). This new cortical model (initiated in Pattabiraman et al. 2014) fills holes identified in our previous updated model (Puelles 2014). The concentric cortical map already showed that the pallial amygdala is a *different* neuroepithelial field that is adjacent, but *separate,* from the cortex. The two adjacent fields nevertheless do clearly share general pallial molecular characteristics, and thus are both pallial (Puelles et al. 2000).

This first step allowed us conceptually to investigate next what happens inside the isolated amygdalar pallial field. We eventually produced a *radial model of the pallial amygdala* (Garcia-Calero et al. 2020), which postulates that all amygdalar nuclei originate in different subareas within the ‘caudal telencephalic neuroepithelium’ locus identified by Remedios et al. (2007). The model derives all amygdalar nuclei from no less than 9 molecularly different *amygdalar radial units*, subsumed under 5 macrounits (Fig. 15a), each of which develops molecularly distinct periventricular, intermediate and superficial strata. This new, much more complex amygdalar model now explains easily the previously contradictory data of Gorski et al. (2002), Puelles (2014) and Puelles et al. (2016c). Garcia-Calero and Puelles (2021) reexamined and reinterpreted the *Dbx1* data of Puelles et al. (2016c) within the novel *radial amygdalar model*. They reached the conclusion, consistent with both radial glia pattern and Bielle et al. (2005), that *amygdalar Dbx1* derivatives are intrinsic to the amygdalar field, and cannot be assimilated to *cortical* ventral pallium *Dbx1* derivatives. This is precisely the conclusion that also satisfactorily explains the *Emx1-LacZ* data in Gorski et al. (2002).

In the wake of these advances, the effort made by Remedios et al. (2007) to trace the amygdalar CAS migration to the ‘dorsal pallium’ cortical sector seems outdated and devoid of explanatory meaning, when reexamined under the light of the radial model (and the abandonment of coronal sections). There is simply no interest in extracting amygdalar pallial nuclei from parts of the cortical pallium, because no part of pallial amygdala (including the CAS/NLOT2ms) comes out of any cortical domain. Moreover, the concentric ring cortical model provides wider significance and novel lines of analysis for the hem and antihem functions, admitting other possible organizer sites as well. Note our Fig. 15c illustrates that the dorsal pallium does not even come close to the hem and antihem organizers, because the inner mesocortical and outer allocortical cortical rings are interposed (i.e., these organizers lie topologically outside the entire concentrically organized cortex; interestingly, the gap illustrated by Remedios et al. 2007 that separates ‘longitudinally’ the hem from the antihem relates to the new *amygdalo-allocortical or amygdalo-entorhinal border* proposed by us). This new topologic concept of the cortical field and its organizers recovers the classic notions of allocortex and mesocortex (the two concentric peripheral cortical rings) as the natural evolutionary environment of the central neocortex/isocortex island (Puelles 2001, 2011; Puelles et al. 2019a).

It was thus a commonly held, but *not wholly supported,* or *solidly evidence-based*, assumption of many workers in the pallial developmental field at the time of the Remedios et al. (2007) study that cortical pallial sectors *might or must* have derivatives in the amygdala. It is important to realize that all the referred amygdalar molecular mappings aiming to establish cortical origins of amygdalar parts stood systematically on *coronal sections*, which are oblique to amygdalar radial glia.

The new amygdala model agrees with the facts that the respective cortical and amygdalar structures are *different* (layers versus nuclei). Note that amygdalar neurogenetic stratification is *outside in*, whereas the cortical one is *inside out* (Garcia-Calero and Puelles 2020), not to speak of differential connections and functions. Consequently, pallium models postulating a number of parallel longitudinal pallial sectors extending throughout the whole telencephalon are presumably on the wane. We should study the cortical pallium and the amygdalar pallium as causally separate complex fields, each with quite diverse molecular profiles, and probably with correspondingly diverse causal mechanisms, irrespective of the known partially shared gene patterns.

As regards our third discussion point sketched above, we refer to atlases of developing rodent brains (e.g., Altman and Bayer 1995; Alvarez-Bolado and Swanson 1996; Jacobowitz and Abbott 1997; Foster 1998; Paxinos et al. 2007; Ashwell and Paxinos 2008), which unanimously identify the posterior pallial region (AHi) where the NLOT2ms originates as an integral part of the amygdala. This clearly includes the caudal pallial area where Remedios et al. (2007) electroporated at E11.5 a GFP-fluorescent label, which later nicely concentrated in the CAS migration stream (their Fig. 2). The AHi name (‘amygdalo-hippocampal transition area’) given conventionally in atlases to this posterior amygdalar area actually alludes to its easily visible extensive caudal neighborhood with the hippocampus (and entorhinal cortex), rather than with the distant neocortex. This fact obviously already stood ab initio against the conclusions of Remedios et al. (2007). These authors explicitly identified the ‘dorsal pallium’ as the primordium of the neocortex, and even announced a ‘link between the amygdala and neocortex’ in their title. However, no part of the amygdala is histologically neocortical or eulaminate (i.e., six-layered; Garcia-Cabezas et al. 2019), or even contacts neocortical structures (recent reviews in Barbas 2015; Garcia-Cabezas et al. 2019; Puelles et al. 2019a; Garcia-Calero et al. 2020; see also Paxinos and Franklin 2013, and other rodent brain atlases; see also our Fig. 15c, d). This means that there is no solid anatomic or developmental evidence for the neocortex-amygdala link, irrespective that the posterior amygdala seems to share given molecular markers with both dorsal and medial pallium (as was argued above).

Neuroanatomists know for a long time that the neocortex lies far away from the amygdala. If neocortex really produced the CAS cells, these would *have to migrate* across the interposed mesocortex and allocortex rings that surround the neocortex, possibly having to pass across the entorhinal schizocortex, in order to reach the amygdala beyond the caudal end of the lateral ventricle (see red dot and red arrow schematics in our Fig. 15c, d). Actually, Remedios et al. (2007) did not really think, or wanted to imply, that their postulated ‘neocortical origin’ was *distant*, since they did not explore that possibility. After all, they knew from their crucial electroporation experiment that the migration originates entirely *in front* of the caudal end of the lateral ventricle (compare Fig. 15c, d with their Fig. 2).

These authors accordingly just played with the hypothesis that the relatively unexplored caudal part of the dorsal pallium might extend caudalwards through the caudal gap they had discovered between hem and antihem, passing beyond the caudal end of the ventricle into a similarly unexplored ‘caudal telencephalic neuroepithelium’. This implies an aberrant model of cortical development which we need to criticise, because patterning studies are brought into significant confusion by arbitrary modifications of the ‘area map’ that needs to be explained (see comments in Puelles et al. 2019a).

In holding this hypothesis, Remedios et al. (2007) implied (probably unwittingly) that the limits of neocortex are wrong in all precedent publications, atlases and specialized book chapters on the cortex since Brodmann (1909) and von Economo (1927). These sources do not show that the neocortex extends backwards to contact the posterior pallial amygdala (Garcia-Cabezas et al. 2019; Puelles et al. 2019a). It is a sign of a certain dismissive attitude to conventional neuroanatomy in the molecular era that Remedios et al. (2007) actually got this conclusion published in Nature Neuroscience! However, these authors did not even explore in their Discussion the conventional interpretation suggesting that any cortex immediately caudal to the amygdala most probably had to be of the hippocampal allocortical sort, that is, *mediopallial*. This involves either entorhinal cortex (a variant non-eulaminate sort of allocortex, also termed ‘schizocortex’; Puelles et al. 2019a), or hippocampal subicular or Ammon’s horn areas. We represented these diverse amygdalar caudal vicinity relationships in optimal horizontal sections in Garcia-Calero et al. (2020; e.g., our Fig. 5). Various cortex models supporting this notion were recorded since 1987 (e.g., Swanson 1987; Bayer and Altman 1991; Pattabiraman et al. 2014; Garcia-Cabezas et al. 2019). See also Witter (2012; his Figs.5.1 and 5.2) and Martínez-García et al. (2012; their Fig. 6.3).

Alternatively, the conclusion of Remedios et al. (2007) might imply that the postrhinal, entorhinal and hippocampal cortex domains are actually developmentally and molecularly *neocortical*, against the majority of existing opinions in the field, and a host of inconsistent molecular data (Thompson et al. 2014; Allen Mouse Brain Atlas, and other analogous repositories). We thus think that Remedios et al. (2007) were not aware of this anatomic difficulty, and did not realize that the hypothetic existence of dorsal pallium passing through the hem-antihem gap to contact the amygdala implied a highly improbable *rupture of the allocortical ring* present around the whole mesocortex and neocortex, as was already known in 2007 (Swanson 1987; Bayer and Altman 1991; Fig. 15c).

All this implies that it is possible in 2020 to reinterpret coherently the objectively beautiful and rich results of Remedios et al. (2007) as we presently did here, by substituting a primary posterior amygdalar origin of the CAS/NLOT2ms migration, and leaving the dorsal pallium apart in its central place within the cortical ‘area map’. We eliminated doubtful assumptions based on the use of coronal sections, and other conceptual errors of the near past. We refrained from deciding the issue at hand with hardly specific gene expression patterns (*Lhx2*, *Emx1*), or from believing that selective *cortical* gene patterns inform us about the origin of differently fated *amygdalar* structures, or intrinsic amygdalar migratory phenomena. We will see below that we can insert the duly corroborated gene requirements observed by Remedios et al. (2007), into a different and more complete explanatory rationale that does not involve the dorsal pallium, and does not disrupt on the sly the anatomic traditions preserved by rodent brain atlases and other relevant literature.

The real CAS origin at the amygdalar AHi/PMCo primordium (*posterior* amygdalar unit) is consistent, moreover, with our present results showing that this amygdalar unit *is also the target* of the HyA corridor and its migrated hypothalamic *Sim1*-positive cells. These are fated to help the CAS reach the NLOT2 locus by their apparently* required* presence in this cell stream. Divergent evolution of an equivalent NLOT2 homolog in other vertebrate groups thus may be also linked to co-evolution of the *Sim1*-expressing population and its timely arrival at the pallial amygdala, in order to participate in the NLOT migration stream.

### Precise origin of Sim1 cells targeting the pallial amygdala and Sim1/Otp comparison

Present results suggest that *Sim1*-expressing NLOT2 cells originate along the HyA and Pa areas, since both belong strictly to the same progenitor domain, merely deformed in part during hemispheric evagination. There is the caveat that the Pa (and thus perhaps also the HyA) is presently held to be divided into 3 dorsoventral subdomains with some variant properties (dorsal, central and ventral Pa, or DPa, CPa, VPa; see Fig. 15a; Puelles et al. 2012). All of them produce *Sim1-* and *Otp-*expressing cells (Michaud et al. 1998; Wang and Lufkin 2000), but only the CPa and VPa subdomains display *Brn2* expression, while only DPa co-expresses *Foxg1* (Morales et al. 2021). The dorsoventral subdivision of Pa leads to phenotypic and migratory differential properties before or after the terminal differentiation step of the neurons produced in each sector (Schonemann et al. 1995; Michaud et al. 1998; Wang and Lufkin 2000; note none of these authors were aware of the existence of Pa subdomains; see also Diaz et al. 2015). DPa apparently produces mainly TRH and SST cells, while CPa and VPa jointly produce CRH, AVP and OT cells. We had difficulties in confirming this pattern (except for CRH cells, which are clearly related to CPa) on the basis of mappings at the Allen Developing Mouse Brain Atlas, possibly due to short-range tangential migrations that redistribute the cells. Remarkably, only the TRH cell type appears later in the pallial amygdala (at the PMCo part of the AHi/PMCo posterior complex), though this happens in a much delayed chronology (first tenuous expression at P4; strong signal at P14). Dispersed SST cells are abundant in various amygdalar nuclei, due to massive tangential migration of SST-expressing interneurons from the subpallial diagonal domain (Puelles et al. 2016a). Therefore, we cannot identify potential intrinsically developed, HyA-related SST elements.

Morales et al. (2021) interestingly observed that the DPa subdomain (interpreted by these authors as being telencephalic rather than hypothalamic, due to its coincident *Foxg1* marker signal) extends into a *rostrodorsal part* of our HyA. These authors show that DPa contributes glutamatergic Otp cells to the medial amygdala, extended medial subpallial amygdala, and part of the BSTM nucleus (curiously, all of them targets practically devoid of *Sim1*-expressing cells). The CPa subarea extends instead into the *caudoventral part* of HyA, which presumably leads into the pallial amygdala (as represented in Fig. 15a). This HyA division idea was already advanced by Puelles and Rubenstein (2003, their Fig. 3 schema) and Puelles and Rubenstein (2015, their Fig. 10 schema). In any case, the fact that the wild-type E18.5 NLOT2 expresses both *Sim1* and *Brn2* (Fig. 14e, g) suggests that its hypothalamic cells originate at the extended CPa subarea, rather than the DPa, where *Brn2* reportedly is not expressed (Michaud et al. 1998). Hence, there are at least two molecularly distinct parts dividing lengthwise the HyA corridor, which extend the *Foxg1/Brn2* molecular border existing between DPa and CPa (Fig. 15a). Our observation of overlapping *Sim1* and *Brn2* signal in NLOT2 at E18.5 suggests that the latter marker may render the CPa-HyA subdivision ostensible.

Further studies should explore why *Otp*-expressing cells apparently target preferentially subpallial centers (Morales et al. 2021; Wang and Lufkin 2000; Garcia-Moreno et al. 2010), whereas *Sim1*-expressing cells target selectively the pallial amygdala, and mainly NLOT2 and PaA therein (present analysis). Wang and Lufkin (2000) misidentified the locus of *Sim1* cells within the amygdala (see below).

The NLOT2 migration and the presence of *Sim1* cells in the postnatal NLOT2 are *unaffected* in *Otp* mutant mice, though the *Otp*-expressing cells migrated early on into the MeA are substantially reduced in number after E15.5 (Wang and Lufkin 2000; their Figs. 2i–l, 3). In Fig. 7a, b of the same report it can be noticed that the HyA appears *Otp*-labeled under the terminal sulcus in the *Otp*-LacZ *heterozygote* at E15.5, but not in the *homozygote*, though the MeA is invaded equally. Probably most of the *Otp* cells migrated into MeA arrive there via the subcapsular part of the HyA (LP and EG-C, unpublished observations). Wang and Lufkin (2000) also showed in their Fig. 6e, f (at P1), Fig. 7q, r (at E15.5), and Fig. 8s, t (at E13.5) images of pairs of comparable sections from wild type and *Otp*^−/−^ embryos *reacted for Sim1 transcripts*, which compare perfectly with our present material. The P1 amygdalar cell mass expressing *Sim1* (their Fig. 6e, f) clearly is the NLOT2, though it was mislabeled as ‘MeA’ (there is no *Sim1* signal at the MeA, nor any *Otp* signal at the NLOT, and the position and shape shown correspond to NLOT). The authors apparently were unaware of the fact that the amygdalar targets of *Sim1* and *Otp* cells are different. In fact, it is unclear to us whether something similar occurs also in the hypothalamus, where a subtle differential topography of *Sim1* versus *Otp* cells might have passed undetected, unless the mixed pattern accepted conventionally truly reigns there. The amygdalar E15.5 *Sim1* image of Wang and Lufkin (2000; their Fig. 7q, r) shows a normally labeled NLOT2ms in both heterozygote and homozygote, while the E13.5 image (their Fig. 8s, t) exactly duplicates our Fig. 5g, illustrating incipient *Sim1* penetration of the periventricular *posterior* pallial amygdala (future AHi). It is of interest as well that heterozygotic *Otp*-LacZ whole-mounts show *no labeled HyA* at E11.5, whereas a fully formed HyA appears at E12.5 (Wang and Lufkin 2000; their Fig. 2e, g; in the legend, the authors misidentified the HyA as ‘amygdaloid nuclei’). This result points to an independent, possibly consecutive production of earlier *Sim1-*fated cells versus slightly later *Otp*-fated cells, at least at the HyA. Part of the timing and positional subtleties detected later in the patterns of delayed cell death resulting from *Sim1* or *Otp* loss of function (Michaud et al. 1998; Wang and Lufkin 2000; present results) probably obey to this temporal dissociation.

The production of *Sim1* neurons at the Pa/HyA indeed begins precociously at E10.5 or earlier (Fan et al. 1996), whereas Acampora et al. (2000) detected *Otp* transcripts in the hypothalamus already at E9.5. The corresponding ventricular zone is transiently *Sim1*-positive between E10.5 and E11.5, but the expression becomes subsequently restricted to the mantle layer as of E12.5 (Fan et al. 1996; present results—see Fig. 1m; this coincides with the first emergence at E12.5 of the *Otp*-LacZ positive HyA; Wang and Lufkin 2000; their Fig. 2G). One may thus conjecture that *Otp* cells possibly start to emerge at the HyA once the *Sim1* gene is downregulated at the *ventricular zone*, leaving only *Otp* activated there. This change in *molecular profile* might underlie causally the change in neurogenetic timing between *Sim1* and *Otp* cells at the HyA, and possibly affect as well their differential molecular profile, terminal differentiation, migratory interactions and targets.

### The present concept of the hypothalamo-amygdalar corridor

We described for clarity the evaginated paraventricular HyA corridor as a supracapsular anatomic entity lining ventricularly and periventricularly the floor of the interventricular foramen and the terminal sulcus, in planar continuity with the hypothalamic Pa area and the posterior pallial amygdala (Fig. 15a). However, we must assume, based upon general knowledge on neuroepithelial histogenesis (Nieuwenhuys and Puelles 2016), that the HyA surely develops its own mantle layer containing narrow periventricular, intermediate and superficial strata derivatives. This radially complete HyA territory of the neural wall must end at the pial surface of the telencephalic stalk contiguously with that of the non-evaginated Pa, intercalated between the prethalamic pial surfaces of the prethalamic eminence and the subpallial diagonal band (Fig. 5b, c). We do not know anything about the non-periventricular HyA mantle derivatives, which apparently do not express significantly *Sim1*. They may contain Otp cells, and be lumped with components of the MeA in atlases and publications (Fig. 15a).

Previously we conceived the HyA as a ‘pallial corridor’, that is, as a part of telencephalic pallium that descended behind the MeA and other parts of the ganglionic eminences to contact the alar hypothalamus (implicit in Puelles et al. 2013, 2016a). In a similar vein, we identified the avian counterpart as ‘pallial extended amygdala’, given its parallel disposition relative to the subpallial extended amygdala complex (e.g. Puelles et al. 2007, 2019b). Our present work made us realize finally that the HyA represents permanently an *evaginated portion of the hypothalamic Pa area*, sharing some of its molecular and neurogenetic properties, and even reaching the chorioidal roof plate (see chorioid fissure -chf- in Fig. 15a). The presence of this thin hypothalamic band extending into the roof plate separates the telencephalic cortical and amygdalar pallial fields from the diencephalic prethalamic eminence (we previously wrongly supposed that the prethalamic eminence contacts both the hippocampus and the pallial amygdala; e.g., Puelles 2013; Puelles et al. 2015; Alonso et al. 2020a).

The HyA schema in Fig. 15a clarifies our presently updated understanding of the hypothalamo-telencephalic, hypothalamo-subpallial, hypothalamo-pallial-amygdalar, and diencephalo-telencephalic boundaries. It suggests the need to continue thinking about relevant topologic notions relative to this cryptic brain area (Puelles 2019; Puelles et al. 2019a).

### The NLOT2 migratory pathway re-examined within the radial amygdala model

As mentioned above, we proposed recently a radial model of the pallial amygdala (Garcia-Calero et al. 2020). This model postulates the existence of 5 histogenetic pallial-amygdalar radial macrounits. By conservative topological criteria we defined the macrounits as *lateral*, *basal*, *anterior, posterior,* and *retroendopiriform* radial units (there are some subdivisions, leading actually to nine molecularly distinguishable radial structural complexes; see *loc.cit*., and Table 1 in Garcia-Calero and Puelles 2020, 2021). Each of these radial units shows a different combinatorial molecular profile (with variously shared markers), and produces characteristic amygdalar nuclei in a stratified *outside-in arrangement relative to birthdates* (i.e., superficial nuclei are born before intermediate ones, and these before the periventricular nuclei; see discussions in Garcia-Calero et al. 2020, and Garcia-Calero and Puelles 2020, 2021). It is thus meaningless to expect *inside-out* radial migrations within the pallial amygdala (e.g., Subramanian et al. 2009).

Hypothalamic (paraventricular) *Sim1-*expressing cells first translocate into the telencephalon along the HyA; note this displacement is topologically strictly restricted to a caudal neighbourhood (it occurs next to the hypothalamo-diencephalic border) and advances dorsalward (into the telencephalon), even though the HyA appears in whole-mounts as a caudally oriented arc; this bespeaks of the morphogenetic deformation caused by the development of the caudal telencephalic pole (Fig. 1n; see also Fan et al. 1996). At the end of this corridor, the migrating cells bypass the caudalmost part of the MeA, and invade directly the *posterior* radial amygdalar unit (between E13.5 and E14.5; Fig. 15b). They enter it through its *rostromedial subdivision*, intercalated between the MeA and the ventral hippocampus. The *Sim1* cells immediately mix there with local Tbr1 cells, and, together, they start to migrate tangentially rostralwards within the amygdala, advancing now *laterally* to the MeA and alongside the local pallio-subpallial boundary (Fig. 15b). We corroborated the immediate formation of a dense composite *Tbr1/Sim1*-expressing mass which also shows *Neurod1/2/6* and *Zic2* signal, as was initially described by Remedios et al. (2007) and Murillo et al. (2015). See also selective CAS expression of *Dach 1* in the Allen Developing Mouse Brain Atlas. This mass forms the CAS or NLOT2ms (NLOT2ms; Fig. 7l).

At E15.5 the mixed NLOT2 migratory stream continues advancing rostralward through the pallial side of the pallio-subpallial boundary, passing from the *posterior* unit (AHi) to the medial aspect of the *basal* and *anterior* radial units (p, b, a; in Fig. 15a, b). The stream ends what we call the *pallial phase* of its course at a locus just medial to the immature basolateral anterior nucleus (BLA) and slightly above the basomedial anterior nucleus (BMA). BLA and BMA are intermediate strata derived respectively from the *basal* and *anterior* radial units (a, b; Fig. 15a, b; Garcia-Calero et al. 2020). It is at this locus where we noted that *Zic2* expression is downregulated within the NLOT2ms (Murillo et al. 2015 did not comment on this point, visible in their material). *Zic2* expression, apart from defining *selectively* the pallial phase of the NLOT2ms, also happens to be clearly selective for the amygdalar AHi, *excluding any cortical expression* (Fig. 10a, d, g). It also labels distinctly what we first described recently as the *rostrolateral subdivision* of the posterior radial unit or AHi/PMCo complex, which ends superficially separately from the PMCo, next to the PLCo formation (RL; Fig. 10d, f; details in Garcia-Calero et al. 2020).

Some additional *Tbr1*-positive cells from the BLA primordium may incorporate at this level into the NLOT2ms, after becoming displaced lateromedially from the radial axis of the basolateral subunit. This deviation apparently results in the characteristic non-radial *BLA cap* over BMA, and the typical *medial horn* of the BLA nucleus (see Garcia-Calero et al. 2020). Another marker identifying the BLA horn is* Lmo3* (Abellán et al. 2009; their Fig. 1E). This formation penetrates the AA in correlation with the molecularly distinct *amygdalo-olfactory cell stream* (AOS), which thereafter trails the NLOT2ms from this point into the definitive adult NLOT (see these details in Garcia-Calero et al. 2020). We noted here that *Neurod6* (*Math2*) seems to label selectively this possible secondary rostrolateral root of the NLOT2ms (see selectively labeled BLA, BLAcap, BLI, BLP, and NLOT2ms in Fig. 12e–g; a similar combined expression pattern was recently noted in *Etv1* preparations at E15.5 and E18.5 at the Allen Developing Mouse Brain Atlas). Indeed, *Neurod6* signal appears scarcely if at all at the AHi main NLOT2ms origin (Fig. 12d, g), but nevertheless labels the NLOT2ms and the mature NLOT as well (Fig. 12g, h). We thus believe that *Neurod6,* and possibly also *Etv1*, label selectively a minor *basolateral* Tbr1-positive source of the NLOT2ms (b in Fig. 15a, b). This maybe resolves in the AOS stream and the few Tbr1 cells aggregating later at the layer 3 of NLOT (Fig. 9g), also forming a tenuous shell around this nucleus. The AOS cells were first detected by their Azin2-LacZ signal, also present at the BLA and AHi (not shown; see Garcia-Calero et al. 2020), and were later found to express selectively *Er81 (Etv1)* and *Cyp26*, signals, which appear, jointly with *Tbr1*, at the postnatal layer 3 of the NLOT (Fig. 9g, i, j).

In contrast to *Zic2*, transcripts of *Neurod1* and *Neurod2* (as well as of *Dach1*, discovered at the Allen Atlas after our first submission) are selectively present at the AHi, NLOT2ms and definitive NLOT, as well as at the entorhinal and hippocampal cortex in the case of *Neurod1/2* (Figs. 10i, j, 12a–c). *Neurod6* signal is also present at the neighboring cortex (though hardly at E13.5), but does not label significantly the AHi at any stage examined (Fig. 12d, g, h).

The NLOT2ms next passes from the BLA/BMA neighborhood into the anterior amygdalar subpallium (AA; Fig. 15b), crossing the local pallio-subpallial boundary. This is an obvious decision point where the migratory strategy changes, possibly aided by the downregulation of *Zic2* (Figs. 7f, g, 15b). Otherwise, it is of interest in this respect that homozygous mutants lacking *Sim1* function apparently do not progress from the pallial into the subpallial phase of NLOT migration (present results); this may indicate that the *Sim1-expressing* cells present in the migrating stream (particularly at the advancing head of the migration) may be somehow needed to achieve passage into the subpallium. *Sim1* function thus seems required at least for the final subpallial phase in the formation of the NLOT (present results).

It is of further interest in this context that the AA domain is occupied superficially by abundant *Lhx9*/*Lhx2/Tbr1*-positive pallial cells (see *Tbr1* in Fig. 9d, e; *Lhx9/Lhx2* data in Garcia-Calero and Puelles 2021; see also Tole et al. 2005). These elements apparently migrated previously tangentially (subpially) from the precocious ACo population of the *anterior* amygdalar radial unit (a into AA; this migration is not marked specifically in Fig. 15a, b; see Garcia-Calero and Puelles 2021). A good number of subpallium-derived cells expressing *Pax6* or calbindin (not shown) accompany these migrated *pallial* cells at the AA. Tole et al. (2005) reported that absence of *Pax6* function generates anomalies in the formation of NLOT. Moreover, as the NLOT2ms enters the subpallium, it is surrounded *rostrodorsally* (perhaps guided) by a fairly dense *Six3-expressing* population of subpallial cells filling the radial space between the central amygdalar nucleus and the olfactory tuberculum (CeA; OT; Fig. 9k, l).

We conjecture that pallial and subpallial cells populating the AA may produce attractive signals acting on the NLOT2 cells when these reach the BLA/BMA decision point and downregulate *Zic2*. Such attracting signals surely include reelin, as suggested by Remedios et al. (2007), who demonstrated reelin and *Dab1* signals at the AA, and showed experimentally that the progress of the migration depends on the reelin/cdk5 pathway (shared by neocortical radially migrating neurons). It is remarkable that no violation of the pallio-subpallial border occurs until the stream contacts the AA; Fig. 15b). This suggests that the reelin attraction effect only reaches up to the aforementioned decision point.

Finally, the NLOT creates its own encapsulated place (with practically no AA cell mixing) within AA; this locus relates to the subpial passage of the lateral olfactory tract medialwards. Over E16.5-E18.5, the trailing part of the NLOT2ms gradually disappears as the cells reach the target, and the definitive tri-layered NLOT forms in conjunction with the other layer components. The latter have still uncertain origins. Layer 1 was reported to express selectively *Lhx2* (Tole et al. 2005) and *Lhx9* (Remedios et al. 2007), a pattern it possibly shares with the neighboring, likewise olfacto-recipient, superficial BAOT nucleus (García-López et al. 2008; their Fig. 14D). Layer 3 expresses partially *Tbr1*, as well as *Azin2-LacZ*, *Er81(Etv1)* and *Cyp26* signals, as we have reported (Fig. 9g, i, j). This pattern apparently relates the layer 3 NLOT cells to the BLA medial horn and the distinctive Azin2-LacZ-positive AOS (Garcia-Calero et al. 2020), though we have also observed a good number of probably tangentially migrated subpallial *Dlx5/6-LacZ-labelled* neurons mixed therein (LP; unpublished observations). As mentioned above, a few *Sim1-*expressing cells named by us *para-anterior cell group* (PaA) remain dispersed medially to the BMA (part of the *anterior* radial pallial amygdalar unit), caudally to the definitive NLOT (PaA; Figs. 6n, o, 13c–f, h–j, 14a, b; see postnatal stages in the Allen Developing Mouse Brain Atlas). We are not sure whether these cells split off from the NLOT2ms, or migrate independently along a subcapsular route from the hypothalamic paraventricular area, or the HyA. Remarkably, a remnant of the *Sim1*-expressing HyA located next to the AHi also remains visible, intercalated between the MeA and the PThEt up to P4, but apparently disappears afterwards (not shown; see Allen Developing Mouse Brain Atlas). Curiously, *Otp* cells are also observed postnatally at this extreme caudal HyA locus, next to the chorioidal tela of the chorioidal fissure; these *Otp* cells persist even in the adult brain (LP; EG-C; unpublished observations).

### Synthesis of loss of function mouse phenotypes affecting NLOT development

Mice mutants for genes such as *Tbr1*, *Emx1/Emx2*, *Lhx2, Pax6, Zic2* (and also *Sim1*, according to present results) affect NLOT formation (Remedios et al. 2004, 2007; Tole et al. 2005; Murillo et al. 2015), but their analysis has not been brought yet to a synthetic conception of how the NLOT migrates and forms under their joint influences. We offer a tentative synthetic interpretation that seems consistent with presently available data, and suggests some possibilities apt to be tested experimentally.

The joint mutation of *Emx1/Emx2* leads to absence of the NLOT (Tole et al. 2005). This phenotype shows mainly a severe loss of hippocampal portions including the dentate gyrus (e.g., Bishop et al. 2003; Shinozaki et al. 2004; Suda et al. 2010; see also Mangale et al. 2008), apparently due to failure of hem patterning effects. We postulate that at least the *rostromedial* subdivision of the *posterior* radial amygdalar unit, which lies just in front of the caudoventral end of the hippocampus and of the attached hem organizer (Fig. 12o, p), possibly lacks in this situation needed hem patterning signals, apart of its intrinsic *Emx1* signal (Remedios et al. 2007; present results; Fig. 12o, p). This molecular abnormality at the origin of the CAS migration would lead to a primary failure of NLOT formation, due to abnormal specification of its origin. Such hem influence upon the *posterior* amygdalar unit may explain as well the existence of several shared hippocampal genes at this locus (Abellán et al. 2014). We do not believe that this implies that the origin of the CAS migration is mesopallial* cortical*, for the same reasons that we discard the notion that it may be dorsopallial (see above), or perhaps caudo-ventropallial (Ruiz-Reig et al. 2018). These notions implicitly (and arbitrarily) refer to cortical pallial sectors, and we are dealing with the pallial amygdala.

A similar case may apply to mutants devoid of *Lhx2* signal (Mangale et al. 2008; Chou and Tole 2019). Since this gene appears strongly expressed in combination with *Emx1* at the posterior radial unit and neighboring allocortex (Fig. 12i–n) at the stage in which the NLOT2ms starts to form. Lack of *Lhx2* function *within the posterior amygdala* possibly alters the normal local specification of the origin of this migration, so that as a result the NLOT2ms does not form. We do not believe that *Lhx2* lack of function phenomena described within cortical pallium, particularly phenomena taking place in the allocortical primordia (apparently not contemplated by Mangale et al. 2008, Subramanian et al. 2009, or Chou and Tole 2019), affect per se the posterior pallial amygdala, unless the hem functions are affected (see case of *Emx1/Emx2*). Secondarily, if the CAS migration should emerge anyway in this mutant (this point can be examined), lack of *Lhx2* function might have a different sort of amygdalar relevance. The *Lhx2/Lhx9* cells migrated into AA may normally participate in generating the anterior amygdalar reelin signal needed for the final attraction of the NLOT2 cells into their definitive AA position (Remedios et al. 2007). Such attraction may result somehow compromised by lack of *Lhx2* function in these AA cells. Interestingly, the pallial amygdalar nuclei co-expressing *Lhx2* and *Lhx9* (BMA; ACo) seem to attract on their own the para-anterior cell group (PaA) of *Sim1*-expressing cells, which forms a persistent medial shell next to the BMA nucleus, thus remaining within the pallial amygdala. This structure apparently resists loss of the *Sim1* signal (Figs. 13a–f, 14a, b), but we lack data about its possible absence in *Lhx2* mutants.

The *Lhx2/Lhx9* expressing amygdalar pallial cells were earlier thought to derive from the cortical ventral pallium (Medina et al. 2004; Tole et al. 2005; Puelles et al. 2016b). However, Garcia-Calero et al. (2020) and Garcia-Calero and Puelles (2021) reconsidered this notion, visualizing the *Lhx9/Lhx2* combination as restricted primarily to the embryonic *anterior* radial amygdalar unit. They also described the secondary tangential migration of superficial BMA/ACo *Lhx9/Lhx2* cells into the AA. García-López et al. (2008) visualized other local migrations of similar cells that penetrate the MeA, a point later verified experimentally by Bupesh et al. (2011).

*Tbr1* and *Zic2* mutants show a disorganization of the migration and the NLOT nucleus is not formed (Remedios et al. 2004, 2007; Murillo et al. 2015). Since the NLOT2ms is a pallial migration, lack of the fundamental pallial Tbr1 marker may cause abortion of the origin of the migration. As regards *Zic2*, we noted that it only appears expressed by the NLOT2ms cells during their pallial phase of migration, and this gene results downregulated afterwards as the stream moves into the subpallium (shown, but not discussed by Murillo et al. 2015; present results). This peculiarity suggests that the migration needs *Zic2* function only during the initial pallial phase (while it proceeds orthogonal to glial processes, according to Remedios et al. 2007), that is, between the *posterior* radial unit origin and the BLA/BMA decision point. This part of the migration possibly does not obey yet the reelin/cdk5 signaling pathway, a mechanism which appears to apply mainly to the second subpallial phase (Remedios et al. 2007; Subramanian et al. 2009), and perhaps even participates directly or indirectly in the downregulation of *Zic2* at the decision point. The shorter CAS migration observed in absence of reelin/cdk5 signaling (Remedios et al. 2007) possibly occurs thanks to *Zic2* in concert with the *Neurod1/2* genes (see also in vitro data of Murillo et al. 2015).

We observed that *Sim1* mutant homozygotes reproduce in absence of *Sim1* function the initial phase of *Sim1*-tau-LacZ-labelled NLOT2ms migration up to the pallial-subpallial decision point. However, once *Sim1* cells start to die at about E15.5-E16.5 (as found by Michaud et al. 1998 in hypothalamic paraventricular *Sim1* derivatives), progression of the NLOT2ms (with majoritary *Tbr1* cells, and a *Sim1/Brn2*-expressing subpopulation) into the subpallial migration phase fails to occur. No cytoarchitectonic or molecular trace of the NLOT nucleus appears subsequently within AA at E18.5 (Fig. 14c–h). This result possibly indicates an intimate migration-facilitating or perhaps trophic relationship needed for the second phase of migration, which unifies the further migration of both *Tbr1*- and *Sim1*-expressing cells within the stream. This interaction seems needed at least from the intermediate decision point onwards, once *Zic2* activity is repressed. Accordingly, *Sim1* function is somehow required to advance the whole migration stream into its second subpallial phase. One interesting possibility is that the *Tbr1-Sim1* interaction makes the newly *Zic2*-negative NLOT2 migrating cells able to *respond* to the subpallial reelin signals related to *Lhx2* and *Pax6* signals within AA, thus allowing the second phase to begin. Moreover, *Sim1* function possibly is also needed less critically during earlier stages in the migration into and inside the amygdala of the *Sim1* cells, since we also observed abnormal accumulations of *Sim1* cells along the HyA at E16.5 (Figs. 13, 14a, b).

Our tentative hypothesis of a second *basolateral* root of the migrating NLOT2 stream, connected to *Neurod6* and *Etv1* signals, maybe does not hold, since we have only circumstantial evidence so far (*Neurod6* or *Etv1* knockouts might be informative). In any case, we think that the notable adult BLA cap and horn elements, which singularly protrude *anti-radially* into the amygdalar subpallium in the wake of the advancing NLOT2ms (as suggested by Garcia-Calero et al. 2020) is perhaps understandable alternatively as a sketched but unfinished reaction to the subpallial attracting reelin signals which bring the NLOT2ms into AA. The AOS cells extending from the BLA horn into the NLOT layer 3 and related shell formation (Garcia-Calero et al. 2020) seem to represent another AA-attraction phenomenon starting at or near the decision point.

Our present synthetic hypothesis accordingly suggests that hem *Emx1/Emx2* expression (with added *Emx1, Lhx2* and *Neurod1/2* signals at the *posterior* radial unit) specifies molecularly the posterior amygdalar territory where the CAS originates (Tole et al. 2005). Failure of this early step, or lack of local *Tbr1* function, compromises the whole migration. Subsequently *Zic2* jointly with* Neurod1/2* and* Dach1* acting at the origin of the migration are crucial for the activation and control of the first phase of migration (pallial steps orthogonal to glial structure; Murillo et al. 2015). The head of the NLOT2ms (which strongly expresses *Dach1*) thereafter downregulates *Zic2* expression, and *Sim1* function is needed at least at the intermediate decision point for the progression of the stream into its second phase of migration into AA, now parallel to radial glia. This phase crucially requires as well an active reelin/cdk5 signaling pathway, as shown experimentally by Remedios et al. (2007). The latter aspect possibly involves *Pax6* function in AA subpallial neurons or in correlative radial glia cells (Tole et al. 2005; Remedios et al. 2007), and perhaps this depends partly on *Lhx2* in the migrated *anterior* pallial amygdalar cells populating AA (Remedios et al. 2004; Subramanian et al. 2009). Whether subpallial *Six3-*expressing cells that cover rostrodorsally the subpallial phase of NLOT2ms migration (present results) are also involved in its control requires investigation; an involvement is suggested by their closeness to the migrating stream (present results; Fig. 9k). A further concurring circumstance is that the *Lhx2* mutant does not develop a normal lateral olfactory tract under the AA and NLOT (Saha et al. 2007); it is so far unclear whether this defect also affects the NLOT2 final migration into the AA.

## Experimental procedures

### Animal preparation and tissue analysis

The day of the vaginal plug was counted as embryonic day E0.5. The brains from sacrificed mouse embryos were dissected out, and fixed overnight in 4% paraformaldehyde in pH 7.4 phosphate-buffered saline (PBS) at 4 °C. The brains were embedded in 4% agarose in PBS, and 100 µm sections were cut in horizontal, sagittal, coronal and oblique planes with a Leica vibratome (VT1000 S), to be processed for in situ hybridization and immunohistochemistry.

The generation and genotyping of mice carrying *Sim1*-tauLacZ was described previously in Marion et al. (2005). Briefly, a gene cassette encoding tauLacZ was inserted into the first exon of the *Sim1* gene to generate the *Sim1*^tau−lacZ^ allele. The β-galactosidase activity protocol for detection of the *Sim1*^tau−lacZ^ allele was also previously described in Marion et al. (2005).

### In situ hybridization

We used the restriction enzymes and polymerases suitable for specific riboprobe synthesis in the presence of digoxigenin-11-UTP. The hybridization protocol used was according to Shimamura et al. (1994). Mouse cDNA probes used for in situ hybridization analysis were *Dlx5* and *Sim1* (J.R. Rubenstein), *Brn2* (J.L.Michaud) and *Lhx9* (our own lab).

### Immunohistochemistry

For immunostaining we followed the protocol published in Garcia-Calero and Scharff (2013). The primary antibodies used in this study were: rabbit anti-Otp (F. Vaccarino), rabbit anti-Tbr1 (1:200; sc-48816, Santa Cruz Biotechnology, Inc), mouse anti-RC2 (1:10; Developmental Studies Hybridoma Bank, Iowa City, IA, USA).

### Image capture, manipulation and figure assembly

Digital photomicrographs were acquired using an Aperio CS2 digitalizing device and a confocal microscope (TCS SP8 AOBS; Leica Microsystems GmbH, Mannheim, Germany). The z-stack images were acquired with LCS software. Digital images were processed with Aperio ImageScope (Leica Microsystems GmbH, Mannheim, Germany), ImageJ (NIH, http://rsb.info.nih.gov/ij) and Adobe Photoshop and Adobe Illustrator softwares (Adobe Systems Mountain View, CA, USA).

## Abbreviations

3v: Third ventricle
a: Anterior radial unit
AA: Anterior amygdala
Aba: Anterobasal nucleus
ac: Anterior commissure
ACo: Anterior cortical nucleus
AHi: Amygdalo-hippocampal area
ant: Anterior radial unit
Arcs: Arcuate nucleus
b: Basal radial unit
bas: Basal radial unit
BLA: Anterior basolateral nucleus
BLAcap: Cap portion over BMA of the anterior basolateral nucleus
BLP: Posterior basolateral nucleus
BMA: Anterior basomedial nucleus
bp: Basal plate
BST: Bed nucleus of the stria terminalis
BSTM: Bed nucleus of the stria terminalis, medial part
c: Caudal
CeA: Central amygdalar nucleus
CGE: Caudal ganglionic eminence
ch: Chorioidal tela
chf: Chorioidal fissure
CPa: Caudal paraventricular nucleus complex
CSPa: Caudal subparaventricular area
Cx: Cerebral cortex
d: Dorsal
Dg: Diagonal band
DPa: Dorsal paraventricular nucleus complex
Ent: Entorhinal cortex
Fi: Fimbria of hippocampus
Hi: Hippocampus
HiACx: Retrocommissural hippocampal allocortex
HP: Hypothalamus
hp1: Hypothalamo-telencephalic prosomere 1
hp2: Hypothalamo-telencephalic prosomere 2
HyA: Hypothalamo-amygdalar corridor
ICM: Intercalated cell mass
if: Interventricular foramen
l: Lateral
l: Lateral radial unit
L1: NLOT nucleus, layer 1
L2: NLOT nucleus, layer 2
L3: NLOT nucleus, layer 3
lat/bas: Lateral/basal radial units
lv: Lateral ventricle
LGE: Lateral ganglionic eminence
m: Medial
M: Mamillary region
MA: Medial amygdala
MCx: Mesocortex
MeA: Medial amygdala
MGE: Medial ganglionic eminence
NCx: Neocortex
NLOT2: Nucleus of the lateral olfactory tract, layer 2
NLOT2ms: Migratory stream of nucleus of the lateral olfactory tract, layer 2
OACx: Olfactory allocortex
P: Pallium
p: Posterior radial unit
p1: Prosomere 1
p2: Prosomere 2
p3: Prosomere 3
Pa: Paraventricular nucleus
PaA: Para-anterior cell group
Pal: Pallidum
ped: Peduncle
PFx: Perifornical population
PM/PRM: Perimamillary/periretromamillary regions
PMCoRL: Posteromedial cortical nucleus, rostrolateral part
POA: Preoptic region
Poa: Preoptic region
post: Posterior radial unit
PPa: Paraventricular nucleus, peduncular part
PT: Pretectum
PTh: Prethalamus
PThE: Prethalamic eminence
PThEt: Prethalamic eminence, telencephalic part
r: Rostral
rep: Retroendopiriform radial unit
SchCx: Schizo-allocortex
Se: Septum
SON: Supraoptic nucleus
SP: Subpallium
St: Striatum
tel: Telencephalon
Th: Thalamus
TPa: Paraventricular nucleus, terminal part
ts: Terminal sulcus
os: Optic stalk
v: Ventral
VMHc: Ventromedial nucleus, core region
VMHs: Ventromedial nucleus, shell region
VPa: Ventral paraventricular nucleus complex
